# Usefulness of diffusion tensor imaging findings as biomarkers for amyotrophic lateral sclerosis

**DOI:** 10.1038/s41598-020-62049-0

**Published:** 2020-03-23

**Authors:** Seol-Hee Baek, Jinseok Park, Yoo Hwan Kim, Hung Youl Seok, Ki-Wook Oh, Hee-Jin Kim, Ye-Ji Kwon, Youngbo Sim, Woo-Suk Tae, Seung Hyun Kim, Byung-Jo Kim

**Affiliations:** 1 Department of Neurology, Korea University Anam Hospital, Korea University College of Medicine, Seoul, 02841 Republic of Korea; 2 Department of Neurology, Hanyang University College of Medicine, Seoul, 04763 Republic of Korea; 3 Department of Neurology, College of Medicine, Hallym University Sacred Heart Hospital, Anyang, 14068 Republic of Korea; 4 Department of Neurology, Keimyung University School of Medicine, Daegu, 42601 Republic of Korea; 5 Brain Convergence Research Center, Korea University Anam Hospital, Korea University Medical Center, Seoul, 02841 Republic of Korea

**Keywords:** Biomarkers, Amyotrophic lateral sclerosis

## Abstract

Amyotrophic lateral sclerosis (ALS) is a neurodegenerative disease. However, no reliable biomarkers have been identified to represent the clinical status. This study aimed to investigate whether diffusion tensor imaging (DTI) findings are useful imaging biomarkers to indicate the clinical status of ALS patients. Ninety-six probable or definite ALS cases and 47 age- and sex-matched, normal controls were enrolled. Demographic and clinical data were collected at the time of DTI. DTI data were acquired using a 3-Tesla magnetic resonance imaging scanner and analysed by voxel-wise statistical analyses for fractional anisotropy, axial diffusivity, radial diffusivity, mean diffusivity, and mode of anisotropy. Compared with the healthy control group, the ALS group had significant differences in DTI scalars in the diffuse tracts of the brain, which was predominant in the corticospinal tract at the brainstem and cerebellar peduncle area. Furthermore, the DTI values correlated with the ALS functional rating scale-revised (ALSFRS-R) scores and the delta ALSFRS-R score representing the rate of disease progression. The subgroup analysis revealed a more severe and widespread brain degeneration was observed in rapidly progressive ALS. Therefore, our results suggest that DTI findings are useful as imaging biomarkers for evaluating the clinical severity and rate of disease progression in ALS.

## Introduction

Amyotrophic lateral sclerosis (ALS) is a heterogeneous disease in many aspects, including clinical phenotypes and genetics^1^. The identification of genes linking ALS with frontotemporal dementia (FTD) and the emerging concept of multisystem proteinopathies have changed the widely held belief that ALS affects only the motor neuron system^2,3^. This heterogeneity has been recently emphasised, not only in the designing of clinical trials with stratification or enriched enrolment criteria but also in our understanding of the post-hoc analysis of responders and non-responders for the specific treatment. Therefore, stratification of patient groups according to clinical phenotypes (including diverse motor/psychological findings and genetic information) should be considered while designing clinical trials in heterogeneous ALS populations. In addition, objective biomarkers that can be used to reflect the disease status and predict prognosis could be valuable for the diagnosis and treatment of ALS and the design of clinical trials.

With advances in brain imaging techniques, functional and microstructural changes in the central nervous system (CNS) have been investigated. Specifically, diffusion tensor imaging (DTI) is one of the most useful magnetic resonance imaging (MRI) techniques for examining microstructural changes in the CNS^4^. Multiple DTI scalars extracted from raw DTI data reflect the various pathological changes. Fractional anisotropy (FA) is the most widely used scalar in DTI and indicates various characteristics of axon fibres, including the number and size of axon fibres, and the density of crossing fibres^5^. Axial diffusivity (AD) has been suggested to be sensitive to axonal pathologies; radial diffusivity (RD) has been shown to be sensitive to myelination; and mean diffusivity (MD) has been shown to be related to cellularity, oedema, and necrosis, which may reflect the white matter tissue microstructure that affects functional connectivity^6–9^. In addition, the mode of anisotropy (MO) has been shown to be sensitive to crossing fibres^10^.

Previous studies using DTI have demonstrated the widespread degeneration of the brain in ALS^11–15^. Notably, decreased FA in the corticospinal tract (CTS) is the main DTI finding in ALS. However, previous studies have mostly focused on FA. Some studies used AD, RD, and MD values along with FA values to evaluate ALS (Supplementary Table S1)^14,16–24^. However, most studies had a relatively small number of participants with wide diagnostic certainty according to the El Escorial criteria. To the best of our knowledge, two studies have been conducted with a large number of ALS patients: one with 253 patients with ALS, while the other with 67 ALS patients^25,26^. However, these studies used only the FA value. A study that used multiple DTI scalars (FA, AD, RD, and MD) with a large number of ALS participants (n = 87) investigated only the corticospinal tract from the primary motor cortex to the cerebral peduncle^16^.

This study aimed to investigate whether DTI findings can be reliable imaging biomarkers for evaluating the clinical phenotype and disease severity of ALS. We examined multiple DTI scalars in ALS cases compared with those in normal healthy subjects, as well as the correlation of DTI findings with clinical parameters. In addition, we examined differences in DTI findings according to clinical manifestations, such as symptom onset regions, cognitive status, and the rate of disease progression.

## Results

### Clinical characteristics of the subjects

**Figure 1:**
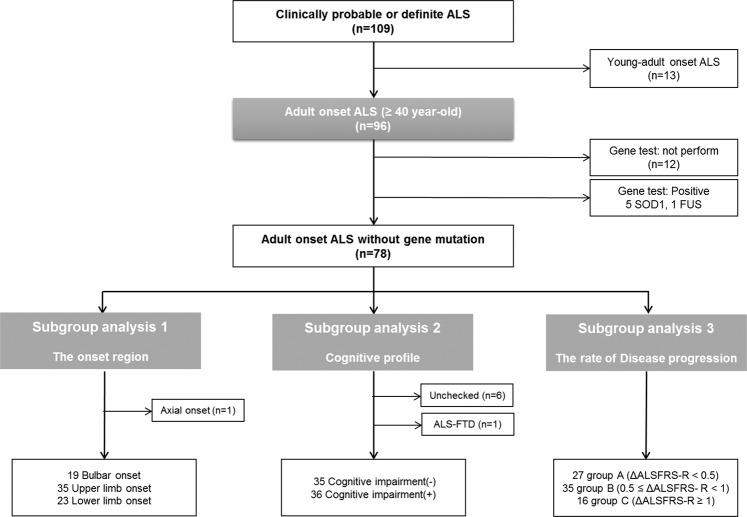
Flow chart of identification of patients with amyotrophic lateral sclerosis. We reviewed 109 patients with clinically probable or definite amyotrophic lateral sclerosis; and excluded 13 patients who manifested the first symptoms before 40 years of age. Subgroup analyses were performed according to the symptom onset regions, cognitive profiles, and the rate of disease progression. Only ALS patients without genetic mutations were included in subgroup analyses.

In total, 96 ALS patients and 47 NCs were included in this study (Fig. 1). The demographic and clinical characteristics of the subjects are summarised in Table 1. The mean age at time of DTI study entry and the proportion of men did not statistically differ between the ALS and NC groups. The mean age at disease onset was 55.4 years, and the mean disease duration was 20.68 months. The mean ALSFRS-R score was 37.57. Among the 84 genotyped patients, six showed pathogenic mutations (5 had *SOD1* mutations, and one had *FUS* mutation).

**Table 1:** Demographic and clinical characteristics of the study population.

Variables	Amyotrophic lateral sclerosis	Normal controls	p-value
Number of patients	96	47	
Males	58 (60.4)	28 (59.6)	0.532
Age at the time of DTI study, years	55.40 (8.39)	57.49 (10.15)	0.187
Age at onset, years	53.27 (8.44)	—	
Disease duration at the time of DTI study, months	20.68 (18.4)	—	
Symptom onset regions		—	
Bulbar	24 (25.0)	—	
Upper Limb	42 (41.7)	—	
Lower Limb	32 (33.3)	—	
ALSFRS-R at the time of DTI study	37.57 (5.61)	—	
ΔALSFRS-R*	0.70 (0.49)	—	
Cognitive function^†^			
Normal	40 (46.5)	—	
Impaired	45 (52.3)	—	
ALS-FTD	1 (1.2)	—	
Gene mutation^‡^	6 (7.1)	—	

Descriptive summaries are presented as numbers with percentages for categorical variables, and means are presented with standard deviation for continuous variables.

*ΔALSFRS-R was calculated using the following equation: (48-ALSFRS-R)/disease duration from onset (months).

^†^86 patients underwent a cognitive function test, Seoul neuropsychological screening battery.

^‡^84 patients underwent the gene test.

ALSFRS-R, amyotrophic lateral sclerosis functional rating scale-revised; ALS-FTD, amyotrophic lateral sclerosis-frontotemporal dementia.

### Comparison of DTI findings

**Figure 2:**
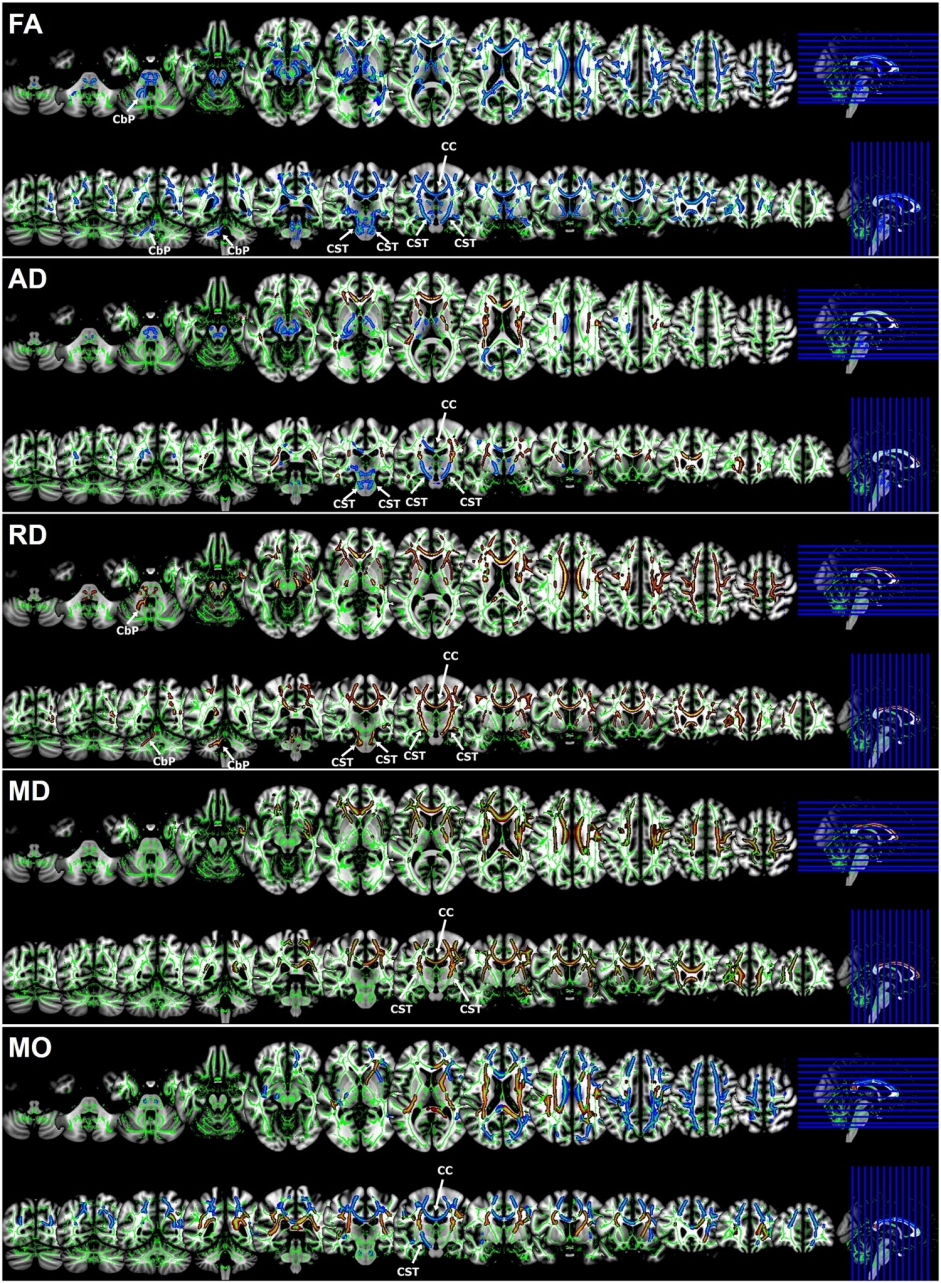
Tract-based spatial statistics: areas of significant differences in each diffusion tensor imaging parameter in amyotrophic lateral sclerosis patients compared with normal controls. Comparisons of diffusion tensor imaging between amyotrophic lateral sclerosis (ALS) and normal controls: fractional anisotropy (FA) maps showed the decreased FA value in the corpus callosum (CC), corona radiata (CR), cerebral peduncle (CP), corticospinal tract (CST), and cerebellar peduncle (CbP). On axial diffusivity (AD) maps, ALS had significantly decreased AD values in the bilateral CST at both the internal capsule and brainstem level, whereas there were significantly increased AD values in the CC and bilateral CR. Radial diffusivity (RD) and mean diffusivity (MD) values of ALS were increased in the CC and bilateral CR. In the mode of anisotropy (MO) image, ALS had significantly decreased MO values in the CC and left CST, whereas there were significantly increased MO values in the bilateral internal capsule.

The TBSS analysis revealed significantly decreased FA values, increased RD and MD values, and a mixed pattern of AD and MO values for patients with ALS (Fig. 2). The FA map revealed significantly decreased FA values in the corpus callosum (CC), bilateral corticospinal tract (CST), and bilateral cerebral peduncle (CP), and cerebellar peduncle (CbP) in ALS patients (*p* < 0.05). In particular, ALS patients exhibited significantly decreased FA values in the bilateral CST at the brainstem level (*p* < 0.05). The AD map revealed significantly decreased AD values in the bilateral CST at both the internal capsule and brainstem level, whereas significant increases were observed in the CC and bilateral corona radiata (CR) in ALS patients (*p* < 0.05). Both RD and MD maps revealed significantly increased values in the CC and bilateral CR in ALS patients (*p* < 0.05). In addition, ALS patients had significantly increased RD values in the bilateral CP and left CbP (*p* < 0.05). The MO map revealed significantly decreased MO values in the CC and left CST, whereas significantly increased MO values were observed in the bilateral internal capsule in ALS patients (*p* < 0.05).

In the VOI analysis (Supplementary Fig. S2 and Table S2), ALS patients exhibited significantly decreased FA and AD values in the bilateral CST at the brainstem level (*p* < 0.05), whereas no statistical differences were observed in RD, MD, and MO values. In addition, ALS patients exhibited significantly increased RD, AD, MD, and MO values in the CR (*p* < 0.05), but no statistical differences were found in FA values. Significantly decreased FA values along with increased AD, RD, and MD values were observed in the CC (*p* < 0.05) in ALS patients. In addition, ALS patients had significantly decreased FA and AD values, but increased RD values in the CP. In the CbP, ALS patients exhibited significantly decreased FA values but increased RD and MD values (*p* < 0.05).

### Correlations between DTI findings and clinical parameters

The ALSFRS-R score was positively correlated with the FA values of the CC (r = 0.297, p = 0.004), bilateral CST at the brainstem level (right: r = 0.366, p < 0.001 and left: r = 0.451, p < 0.001), bilateral CP (right: r = 0.384, p < 0.001 and left: r = 0.278, p = 0.007), and bilateral superior and inferior CbP (right: r = 0.305, p = 0.003 and r = 0.307, p = 0.003, respectively; left: r = 0.436, p < 0.001 and r = 0.273, p = 0.008, respectively). However, both the RD and MD values negatively correlated with the ALSFRS-R score at the initial evaluation in the CC (r = −0.331, p = 0.003 and r = −0.329, p = 0.001, respectively), bilateral CST at the brainstem level (right: r = −0.333, p = 0.001 and r = −0.208, p = 0.046 and left: r = −0.411, p < 0.001 and r = −0.243, p = 0.019), right superior CR (r = −0.297, p = 0.004 and r = −0.321, p = 0.002, respectively), left CP (r = −0.310, p = 0.002 and r = −0.239, p = 0.021 respectively), right medial lemniscus (r = −0.287, p = 0.005 and r = −0.291, p = 0.005, respectively), and bilateral inferior CbP (right: r = −0.311, p = 0.002 and r = −0.297, p = 0.004 and left: r = −0.288, p = 0.005 and r = −0.255, p = 0.014, respectively). Supplementary Table S3 summarises the correlation coefficient between the ALSFRS-R score at initial examination and DTI parameters. In addition, the rate of disease progression (ΔALSFRS-R) was negatively correlated with the FA values in the CC (r = −0.216, p = 0.004), left CST (r = −0.265, p = 0.010). However, both the RD and MD values positively correlated with the rates of disease progression in the CC (r = 0.244, p = 0.018 and r = 0.240, p = 0.020, respectively), bilateral inferior CbP (right: r = 0.215, p = 0.038 and r = 0.275, p = 0.008 and left: r = 0.213, p = 0.040 and r = 0.208, p = 0.045, respectively), and right superior longitudinal fasciculus (r = 0.280, p = 0.007 and r = 0.269, p = 0.009, respectively). Supplementary Table S4 summarises the correlation coefficient between ΔALSFRS-R and DTI parameters.

### Subgroup analyses

#### Analysis according to the symptom onset regions

A total of 78 ALS patients without known genetic mutations were classified into three subgroups according to the symptom onset regions. One patient who exhibited axial weakness as an initial symptom was excluded. The mean age at onset and study entry was older in the bulbar-onset group than in both the upper- and lower-limb-onset groups. The mean bulbar FRS scores were significantly lower in the bulbar-onset ALS subgroup than in the other subgroups. The mean upper and lower limb FRS scores were significantly lower in the upper- and lower-limb-onset ALS groups, respectively. No differences were found between the mean respiratory FRS scores of the three subgroups. The clinical characteristics of the subgroup are summarised in Table 2.

**Table 2:** Demographic and clinical information of subgroups.

	Subgroup analysis 1				Subgroup analysis 2			Subgroup analysis 3^*^			
	Bulbar	Upper Limb	Lower Limb	*p*	ALS without CI	ALS with CI	*p*	Group A	Group B	Group C	*p*
Number of patients	19	35	23		35	36		27	35	16	
Sex, male (%)	8 (42.1)	24 (68.8)	16 (69.6)	0.217	23 (65.7)	25 (69.4)	0.737	17 (63.0)	21 (60.0)	11 (68.8)	0.835
Age at DTI study, years	61.74 (8.91)^a,b^	53.74 (7.51)^a^	53.48 (6.68)^b^	0.001	52.91 (8.06)	58.11 (7.51)	0.006	56.89 (7.23)	55.29 (8.02)	54.81 (10.76)	0.670
Age at onset, years	59.84 (8.65)^a,b^	51.66 (7.89)^a^	51.19 (6.18)^b^	<0.001	51.23 (8.02)	55.78 (7.40)	0.017	53.85 (7.25)	53.63 (8.10)	53.38 (10.95)	0.984
Disease duration at the time of DTI study, months	18.99 (15.72)	19.12 (16.21)	24.27 (24.45)	0.547	15.99 (10.02)	22.67 (21.60)	0.101	31.68 (27.02)^a,b^	16.13 (8.54)^a^	10.89 (3.64)^b^	<0.001
ALSFRS-R at the time of DTI study	37.84 (5.43)	36.83 (5.94)	37.29 (4.91)	0.790	38.74 (4.51)	37.42 (5.38)	0.265	40.3 (4.65)^a,b^	36.60 (5.91)^a^	34.31 (3.83)^b^	0.001
ΔALSFRS-R^†^	0.74 (0.61)	0.78 (0.53)	0.66 (0.39)	0.695	0.73 (0.53)	0.69 (0.50)	0.714	0.29 (0.11)^a,b^	0.74 (0.12)^b,c^	1.44 (6.34)^a,c^	<0.001
ALSFRS-R, bulbar	8.59 (1.37)^a,b^	10.34 (1.40)^a^	10.58 (1.35)^b^	<0.001	10.71 (1.10)	9.52 (1.60)	0.001	10.176 (1.47)	9.903 (1.73)	9.85 (1.57)	0.781
ALSFRS-R, upper limb	9.76 (1.48)^a^	6.79 2.76)^a^	8.42 (1.92)	<0.001	8.52 2.13)	8.14 (2.55)	0.534	9.30 (2.29)^a^	7.57 (2.50)^a^	7.23 (2.59)	0.017
ALSFRS-R, lower limb	6.29 (1.80)^a^	5.21 (1.97)	3.26 (1.56)^a^	<0.001	5.00 (2.11)	5.28 (2.03)	0.609	5.57 (2.31)	4.93 (2.20)	4.00 (1.35)	0.108
ALSFRS-R, respiratory	10.71 (1.45)	11.31 (0.89)	11.42 (1.07)	0.120	11.39 (1.09)	11.03 (1.18)	0.233	11.70 (0.70)^a^	11.13 (0.97)	10.46 (1.61)^a^	0.005

Descriptive summaries are presented as numbers with percentages for categorical variables, and means are presented with standard deviation for continuous variables. Different superscript letters indicate significant differences between groups at α = 0.05 level by Bonferroni’s t-test.

^*^Subgroup analysis 3 subjects were classified into the following three groups according to the rates of disease progression: Group A (ΔALSFRS-R < 0.5), Group B (0.5 ≤ ΔALSFRS-R < 1), and Group C (ΔALSFRS-R ≥ 1).

^†^ΔALSFRS-R was calculated using the following equation: (48-ALSFRS-R)/disease duration from onset (months).

ALS, amyotrophic lateral sclerosis; CI, cognitive impairment; ALSFRS-R, amyotrophic lateral sclerosis functional rating scale-revised.

In the VOI analysis (Supplementary Table S5), significantly decreased FA values were observed in the CC, bilateral CST at the brainstem level, and bilateral CP in all ALS subgroups compared with those in NCs. In addition, significantly decreased AD values were found in the bilateral CST at the brainstem level and left CP in all ALS subgroups. Patients in both upper- and lower-limb-onset ALS groups exhibited significantly decreased FA values in the CbP and increased RD values in the CbP compared with those in the NC group. However, no significant differences in DTI data were found among the ALS subgroups.

#### Analysis according to the cognitive profiles

Among the 78 ALS patients without genetic mutations, 72 who underwent cognitive screening testing were subgrouped according to the presence of cognitive dysfunctions. One patient with FTD was excluded. The mean ages at the time of DTI study entry and disease onset were younger in the ALS patients with normal cognition than in those with cognitive impairment. The ALSFRS-R score of the bulbar section was lower in ALS patients with cognitive impairment than in those with normal cognition. The subgroup comparisons of clinical characteristics are summarised in Table 2.

In the VOI analysis (Supplementary Table S6), patients in both ALS subgroups had significantly decreased FA values in the CC, bilateral CST at the brainstem level, right CP, right posterior limb of internal capsule (PLIC), and left superior CbP compared with those in the NC group. In addition, a significantly decreased AD was observed in the bilateral CST at the brainstem level, and a significantly increased RD was found in the bilateral inferior CbP in both ALS subgroups. ALS patients with cognitive impairment had significantly increased AD values in the right medial lemniscus compared with those in ALS patients with normal cognitive function or NCs (p = 0.0003). However, there were no significant differences in FA, RD, MD, or MO between the ALS subgroups.

#### Analysis according to disease progression rates

A total of 78 ALS patients without genetic mutations were classified into three subgroups according to the disease progression rates. The mean ages at disease onset and study entry did not differ between the subgroups. Patients in Group A (ΔALSFRS-R < 0.5) had a longer disease duration than those in the other two groups. The subgroup comparisons of clinical characteristics are summarised in Table 2.

In the VOI analysis (Supplementary Table S7), significantly decreased FA values were found in the CC, the bilateral CST at the brainstem level, bilateral CP, right PLIC, and bilateral CbP in Group C compared with those in NCs. In addition, patients in Group C had significantly decreased FA and MO values in the left superior CbP compared with those in Group A (p = 0.0009 and p = 0.0010, respectively). Compared with NCs, patients in Group C had significantly increased RD values in the CC, bilateral CbP, right CP, and right PLIC. However, there were no significant differences in AD, RD, and MD among the ALS subgroups.

## Discussion

Our study demonstrated that patients with ALS had widespread changes in DTI values, especially in the bilateral CST of the brainstem and CbP areas. The DTI values in these areas were correlated with the functional status (ALSFRS-r scores) and the rate of disease progression (ΔALSFRS-R). In the subgroup analysis based on the rate of disease progression, further changes in DTI values representing more severe brain degeneration were found in the rapidly progressive ALS group (ΔALSFRS-R ≥ 1) compared with the other subgroups. Therefore, our findings suggest that DTI findings have potential as imaging biomarkers for representing the clinical and functional status in ALS.

Our study found significant differences in the DTI values between patients with ALS and NCs in the CST at the brainstem level and CbP area using multiple DTI parameters. Anatomically, the CST is the motor pathway extending from the cerebral cortex to the spinal cord, and the CbP is the connecting pathway between the brainstem and cerebellum. In our study, significantly decreased FA and AD values in the CST at the brainstem level were observed. Since the tract fibres are tightly packed in the brainstem, the DTI values at the brainstem level could be more sensitive to structural changes. Another finding of our study is the significantly decreased FA value along with increased RD and MD values at the CbP. Little is known regarding the involvement of the cerebellum and cerebrocerebellar connectivity in ALS. Few studies have demonstrated the degeneration of the cerebellum in ALS^27,28^. Moreover, recent studies have reported the alteration of cerebrocerebellar connectivity in motor neuron disease^29,30^. These findings could suggest the involvement of the cerebellum and its connectivity in ALS, although further studies are needed to elucidate the role of the cerebellum in ALS. Taken together, ALS could manifest as widespread degeneration of the CNS, including the lower brainstem and cerebellum. Thus, a whole-brain DTI protocol (which covers the entire brain, including the brainstem and cerebellum) would be needed when performing DTI in the study of ALS.

Our study showed a weak yet significant correlation between DTI values and the clinical data (the ALSFRS-R score and ΔALSFRS-R from the onset). These correlations were observed in not only FA values but also MD and RD values. Specifically, the correlations between the clinical data and the values of FA, RD, and MD were predominant in the motor tracts, especially CC, CST, and CbP areas. We also demonstrated that the rapidly progressive ALS group had more severe neurodegeneration in CST, CP, and CbP in the subgroup analysis. The ALSFRS-R score and rate of disease progression (ΔALSFRS-R) are promising predictors of prognosis in ALS^31–33^. Therefore, our findings could imply that DTI may be used to investigate the clinical severity and the rate of disease progression in ALS. Several studies have previously reported the correlation between DTI values and clinical data. Previous studies have also reported that FA values were related to the ALSFRS score, upper motor neuron score, and rate of disease progression^11,14,19,21,34^, and the MD value was correlated with the disease duration^11^. Furthermore, longitudinal studies have shown significantly decreased FA values^23,26,28^ and increased RD values^20^. The changes in DTI values correlated with those of the ALSFRS-R score^26^. Taken together, DTI has an application potential as imaging markers representing the rate of disease progression and functional status in ALS.

ALS has heterogeneous phenotypes in terms of the symptom onset regions and cognitive function. However, what determines the ALS phenotypes remains unclear. In our study, no significant differences in DTI were observed between bulbar- and limb-onset ALS. We also could not identify differences in DTI findings between ALS patients with and without cognitive impairment. In previous studies investigating topographical distributions of brain degeneration according to the symptom onset regions, some demonstrated that bulbar-onset ALS had significantly decreased FA in the corticospinal tract^11,20^, whereas others found no significant differences between bulbar- and limb-onset ALS^35,36^. In addition, previous studies investigating distinct differences in DTI findings according to the cognitive functional status demonstrated that ALS patients with cognitive impairment had more widespread degeneration in the white matter, including not only the motor tract, CST, and CC but also the extra-motor regions, particularly the frontal lobe^18,37^. These findings may suggest that DTI seems to be a less effective tool for distinguishing the phenotypes of ALS; nonetheless, it may postulate that ALS manifests as widespread white matter degeneration in the brain, regardless of the phenotypes. Further investigations are warranted to determine the correlation between DTI and ALS phenotypes.

Although our study was conducted retrospectively, a large number of ALS patients were enrolled from the prospectively registered patient database at two university-affiliated hospitals. In addition, only patients with clinically probable or definite ALS according to the El Escorial criteria were included in the analysis to enhance the reliability of this study.

In conclusion, DTI parameters have demonstrated widespread degeneration of the brain in ALS, and the changes in DTI findings exhibited significant correlations with the ALSFRS-R score at the initial evaluation and the rate of disease progression in ALS. Our results indicate that DTI findings could be reliable imaging biomarkers for assessing the clinical severity and rate of disease progression using ΔALSFRS-R in ALS.

## Methods

### Study population and clinical data collection

We reviewed the medical records of all ALS patients who were prospectively enrolled in the ALS Registry of two university-affiliated hospitals since 2008. Patients with definite or probable ALS (according to the revised El Escorial criteria) were included in this study^38^. Patients who did not undergo DTI and whose first symptoms manifested before 40 years of age^39^ were excluded from this study. Age- and sex-matched healthy volunteers were recruited as normal controls (NCs).

Data on demographic and clinical characteristics were collected. The functional status at the time of DTI scanning was examined using the Amyotrophic Lateral Sclerosis Functional Rating Scale-Revised (ALSFRS-R). The rate of disease progression at the time of DTI scanning was evaluated using the delta ALSFRS-R score (ΔALSFRS-R), which was calculated using the following equation: (48-ALSFRS-R at initial evaluation)/disease duration from onset (in months). The ALSFRS-R scores were divided into four parts, i.e., bulbar, upper limb, lower limb, and respiratory scores.

To exclude the genetic influence on DTI analysis, we screened six genes, including *SOD1*, *FUS*, *TARDBP*, *OPTN*, *AND*, and *C9orf72*, which were regarded as ALS-FTD spectrum-related genes in all participants^40,41^. In addition, neuropsychological assessment was performed using the Seoul neuropsychological screening battery^42^. We defined cognitive impairment as the 16^th^ percentile (one standard deviation) below the healthy controls. The participants were categorised into two groups according to the revised ALS-FTD diagnostic criteria^43^: ALS without cognitive impairment and ALS with cognitive impairment, including patients with ALS-FTD or patients with cognitive or behavioural impairment.

This study was approved by the Institutional Review Board (IRB) of Korea University Anam Hospital (IRB number: 2015AN0334). Our ethics committee waived the requirement for written informed consent by participants because this study was conducted by reviewing retrospective data. In addition, this study was performed in accordance with the Declaration of Helsinki and the relevant institutional guidelines and regulations.

### MRI protocol

Brain DTI were acquired using a 3.0-T Achieva MRI system (Philips Medical Systems, Best, the Netherlands) with a 32-channel sensitivity encoding (SENSE) head and neck coil. DTI images were acquired with transverse orientation using a pulse sequence with a single shot spin-echo diffusion-sensitised echo-planar imaging sequence (15 gradient directions plus B_0_ image with b-value = 800 s/mm^2^, repetition time/echo time = 13898 ms/55 ms, field of view = 224 × 224, matrix = 112 × 112, number of average = 2, slice thickness = 2.0 mm, and flip angle = 90°). The SENSE acceleration factor was not applied to increase the signal to noise ratio.

### DTI analysis

The Functional MRI of the Brain (FMRIB) Software Library (FSL version, 5.0.9; Oxford, U.K.; http://www.fmrib.ox.ac.uk/fsl/) was used for pre-processing raw DTI data. The voxel-wise statistical analyses of the fractional anisotropy (FA), axial diffusivity (AD), radial diffusivity (RD), mean diffusivity (MD), and mode (MO) data were performed using tract-based spatial statistics (TBSS)^44^. A binary brain mask image was segmented from each DTI image using the brain extraction tool. The eddy current distortions and head movements of the DTI images were corrected using FSL’s ‘eddy’ tool^45^, and then, a binary brain mask image was segmented from each DTI image using the brain extraction tool. Images of FA, Eigenvalue (L1, L2, and L3), MD, and MO were generated using FSL’s ‘DTIFIT’ tool. All FA images were nonlinearly transformed to the FMRIB58 _FA template image and were resampled into a 1 × 1 × 1 mm^3^ standard space (Montreal Neurological Institute 152 standard) using the FMRIB Nonlinear Image Registration Tool. Subsequently, the transformed FA maps were averaged to produce a mean FA image, which was then used to generate FA skeleton white matter tracts of all subjects. The skeletonised FA maps were fed into a voxel-wise group-level analysis. Using the FA map’s transformation matrix, the AD, RD, MD, and MO images were transformed to a standard space, and skeletonised images of AD, RD, MD, and MO were then generated. The FA skeleton image was set at a threshold of FA ≥ 0.2 to exclude tracts, which could have high inter-subject variability or partial volume effects. Volumes of interest (VOIs) were defined on the basis of Johns Hopkins University International Consortium of Brain Mapping-DTI-81 White-Matter Labels in FSL (Supplementary Fig. S1). Individual means of the FA, AD, RD, MD, and MO values for each anatomical VOI were extracted for further statistical analyses. Using FSL’s ‘randomize’ command^46^, which tests the univariate permutation at each voxel, group differences in FA, AD, RD, MD, and MO were examined using analysis of covariance with 5,000 permutations, with age and sex as covariates, and the confounders were demeaned. The multiple comparison problem was corrected at the cluster level using the threshold-free cluster enhancement^47^ to a family-wise error rate of *p* < 0.05.

### Statistical analysis

Comparisons of DTI findings between patients with ALS and NCs were performed using a generalised linear model with sex and age at the time of DTI as covariates. The resulting data were corrected for multiple comparisons using a false discovery rate method, with *p* = 0.05 as the threshold for significance^48^. For clinical data, categorical and continuous variables were compared using the Chi-squared test and Student’s *t*-test, respectively. The correlation analysis was performed using partial correlation analysis (adjusting for sex, age at the time of DTI scanning, and disease duration from onset) between clinical data and DTI parameters.

In addition, three subgroup analyses were performed. In the first subgroup analysis, ALS patients were divided into three subgroups based on the symptom onset regions: bulbar, upper limb, and lower limb. In the second subgroup analysis, ALS patients were divided into two subgroups based on the cognitive profiles: ALS with or without cognitive impairment. In the third subgroup analysis, ALS patients were classified into three subgroups according to the rate of disease progression: Group A (ΔALSFRS-R < 0.5), Group B (0.5 ≤ ΔALSFRS-R < 1), and Group C (ΔALSFRS-R ≥ 1). In the subgroup analyses, only subjects without genetic mutations were included. Repeated measures ANOVA with within-subject factor (VOIs) and between-subject factors (age at DTI and sex) was applied for comparisons between ALS subgroups and NCs. A *p-*value of <0.05 was considered statistically significant in this study. Statistical analyses were performed using SPSS software version 20.0 (IBM Corp., Armonk, NY, USA).

## Supplementary information

Supplementary data.
