# Supplementary material for: Usefulness of diffusion tensor imaging findings as biomarkers for amyotrophic lateral sclerosis
Source: Sci Rep. 2020 Mar 23;10:5199. doi: 10.1038/s41598-020-62049-0 (PMC7090054; doi:10.1038/s41598-020-62049-0)
Supplement: Supplementary file 1 — Supplementary data. [file 41598_2020_62049_MOESM1_ESM.docx]

**Title:**

Usefulness of diffusion tensor imaging findings as biomarkers for amyotrophic lateral sclerosis

**Authors:**

Seol-Hee Baek^1,†^, Jinseok Park^2, †^, Yoo Hwan Kim^3^, Hung Youl Seok^4^, Ki-Wook Oh^2^, Heejin Kim^2^, Ye-Ji Kwon^1^, Youngbo Sim^5^, Woo-Suk Tae^5^, Seung Hyun Kim^2,*^, and Byung-Jo Kim^1, 5,*^

**Authors affiliations:**

^1^ Department of Neurology, Korea University Anam Hospital, Korea University College of Medicine, Seoul, 02841, Republic of Korea.

^2^ Department of Neurology, Hanyang University College of Medicine, Seoul, 04763, Republic of Korea.

^3^ Department of Neurology, Hangang Sacred Heart Hospital, Hallym University Medical Center, Seoul, 07247, Republic of Korea.

^4^ Department of Neurology, Keimyung University Dongsan Medical Center, Daegu, 42602, Republic of Korea.

^5^ Brain Convergence Research Center, Korea University Anam Hospital, Korea University Medical Center, Seoul, 02841, Republic of Korea.

**Supplementary Table 1. Previous studies using various DTI scalars (FA, AD, RD, and MD scalars) in ALS**

| Authors | No. of patients | Diagnostic certainty of enrolled patients | Results | Limitations |
| --- | --- | --- | --- | --- |
| F. Agosta et al.^1^ | 24 | Definite and probable | Significantly increased MD and RD values, significantly decreased FA of the CST bilaterally, and increased AD of the right uncinate fasciculus  FA values of CST was significantly correlated with the rate of disease progression | Small sample size  Only evaluation of CST level  1.5-T MRI |
| L. Sarro et al.^2^ | 11 | Definite, probable, and lab-supported probable | WM tract degeneration is associated with neuropsychologic deficits in patients with ALS. | Small sample size  1.5-T MRI |
| M. Cirillo et al.^3^ | 19 | Definite and probable | Correlations between reduction in FA and increased RD in the body of the CC with the UMN score  Correlation between FA and ALSFRS-R in the associative tracts underneath the left premotor cortex | Small sample size |
| V. Rajagopalan^4^ et al. | 87 | Definite, probable, and possible | Significantly reduced FA and elevated MD values were observed in ALS patients compared with those in controls at the subcortical motor cortex level. Significant differences in AD values were not only seen between control and ALS patients but also between the ALS subgroups, which suggests divergent pathologies in these ALS patients. | Only evaluation of CST level  Inhomogeneous diagnostic certainty  1.5-T MRI |
| Cardenas-Blanco et al.^5^ | 28 | not defined | ALS-B is associated with greater central WM degeneration than ALS-L, possibly contributing to the known worsened prognosis of ALS-B. However, no evidence was found that the spatial distribution of white matter degeneration differs between these groups. | Small sample size  No defined diagnosis certainty |
| Cardenas-Blanco et al.^6^ | 34 | Definite and probable | FA of the CST showed a significant decrease that was driven by a significant increase in RD combined with a trend to decrease in AD. The results indicate that DTI can act as a central nervous biomarker in longitudinal studies. | Small sample size |
| F. Borsodi et al.^7^ | 27 | Probable and possible | A bilateral increase in RD and MD A reduction in FA of the CST Diffusion changes in the parietal and temporal superior longitudinal fasciculus | Small sample size |
| AR Alruwaili et al.^8^ | 30 | Definite and probable | There were greater DTI changes present in ALS with cognitive impairment than ALS with non-cognitively impaired subjects. | Small sample size |
| AF Geraldo et al.^9^ | 14 | Definite, probable, and possible | MD, AD, and RD, besides FA, are able to further detect and characterize neurodegeneration in ALS. | Small sample size  Inhomogeneous diagnostic certainty |
| Y. Bao et al.^10^ | 33 | Definite and probable | Significantly increased RD, MD, and reduced FA, mainly along the CST and the body of the CC  A significant positive correlation between ALSFRS-R scores and FA and a negative correlation between ALSFRS-R and RD was found in left CST. | Small sample size |

DTI, diffusion tensor imaging; ALS, amyotrophic lateral sclerosis; FA, fractional anisotropy; AD, axial diffusivity; RD, radial diffusivity; MD, mean diffusivity; CST, corticospinal tract; CC, corpus callosum; UMN, upper motor neuron; ALSFRS-R, amyotrophic lateral sclerosis functional rating scale-revised; ALS-B, amyotrophic lateral sclerosis with bulbar onset; ALS-L, amyotrophic lateral sclerosis with limb onset.

**References**

1. Agosta, F. *et al.* Assessment of white matter tract damage in patients with amyotrophic lateral sclerosis: a diffusion tensor MR imaging tractography study. *American Journal of Neuroradiology* **31**, 1457-1461 (2010).

2. Sarro, L. *et al.* Cognitive functions and white matter tract damage in amyotrophic lateral sclerosis: a diffusion tensor tractography study. *American Journal of Neuroradiology* **32**, 1866-1872 (2011).

3. Cirillo, M. *et al.* Widespread microstructural white matter involvement in amyotrophic lateral sclerosis: a whole-brain DTI study. *American Journal of Neuroradiology* (2012).

4. Rajagopalan, V., Yue, G. H. & Pioro, E. P. Brain white matter diffusion tensor metrics from clinical 1.5 T MRI distinguish between ALS phenotypes. *Journal of neurology* **260**, 2532-2540 (2013).

5. Cardenas-Blanco, A. *et al.* Central white matter degeneration in bulbar-and limb-onset amyotrophic lateral sclerosis. *Journal of neurology* **261**, 1961-1967 (2014).

6. Cardenas-Blanco, A. *et al.* Structural and diffusion imaging versus clinical assessment to monitor amyotrophic lateral sclerosis. *NeuroImage: Clinical* **11**, 408-414 (2016).

7. Borsodi, F. *et al.* Multimodal assessment of white matter tracts in amyotrophic lateral sclerosis. *PloS one* **12**, e0178371 (2017).

8. Alruwaili, A. *et al.* A combined tract-based spatial statistics and voxel-based morphometry study of the first MRI scan after diagnosis of amyotrophic lateral sclerosis with subgroup analysis. *Journal of Neuroradiology* **45**, 41-48 (2018).

9. Geraldo, A. F. *et al.* Beyond fractional anisotropy in amyotrophic lateral sclerosis: the value of mean, axial, and radial diffusivity and its correlation with electrophysiological conductivity changes. *Neuroradiology* **60**, 505-515, <https://doi.org/10.1007/s00234-018-2012-6> (2018).

10. Bao, Y. *et al.* Radial diffusivity as an imaging biomarker for early diagnosis of non-demented amyotrophic lateral sclerosis. *European radiology* **28**, 4940-4948 (2018).

**Supplementary Figure 1. Indexes of the volumes of interest in our study**
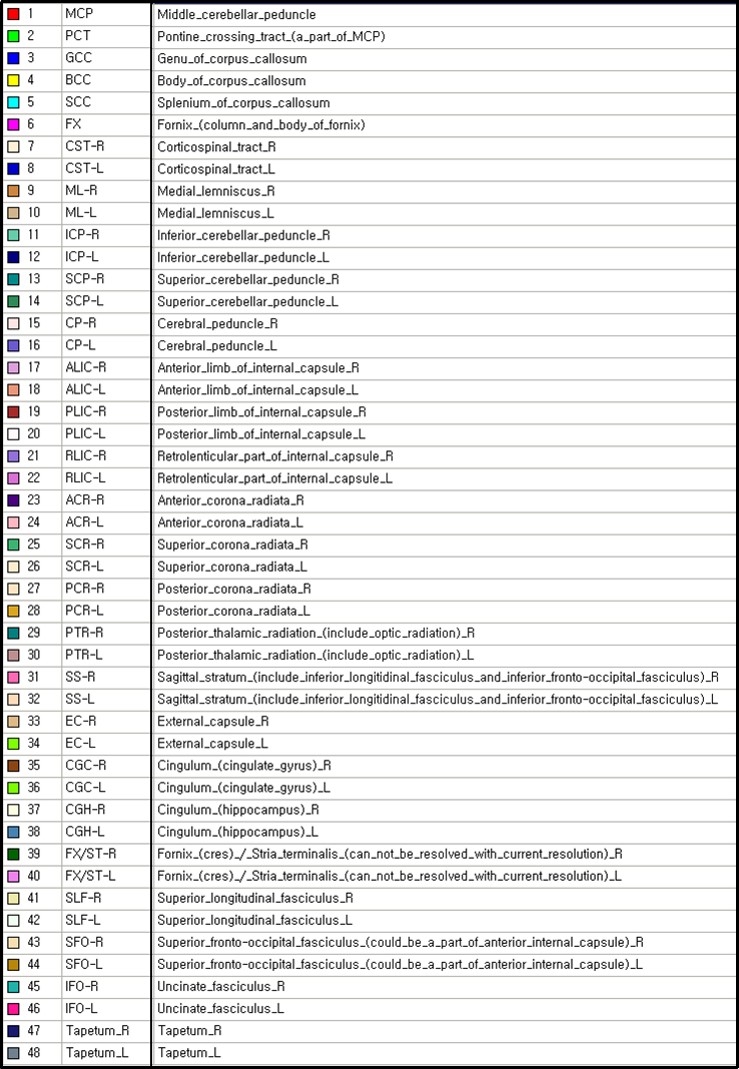


**Supplementary Figure 2. Volumes of interest analysis: areas of significant differences in each diffusion tensor imaging parameter in amyotrophic lateral sclerosis patients (n = 96) compared with normal controls (n = 47)**

Abbreviation: FA, fractional anisotropy; RD, radial diffusivity; AD, axial diffusivity; MD, mean diffusivity; Mo, Mode; GCC, genu of corpus callosum; BCC, body of corpus callosum; SCC, splenium of corpus callosum; ACR, anterior corona radiata; SCR, superior corona radiata; FX, Fornix; CST, corticospinal tract; CP, cerebral peduncle; SCP, superior cerebellar peduncle; ICP, inferior cerebellar peduncle; UF, uncinate fasciculus; R, right; L, left; S, superior; I, inferior; A, anterior; P, posterior.


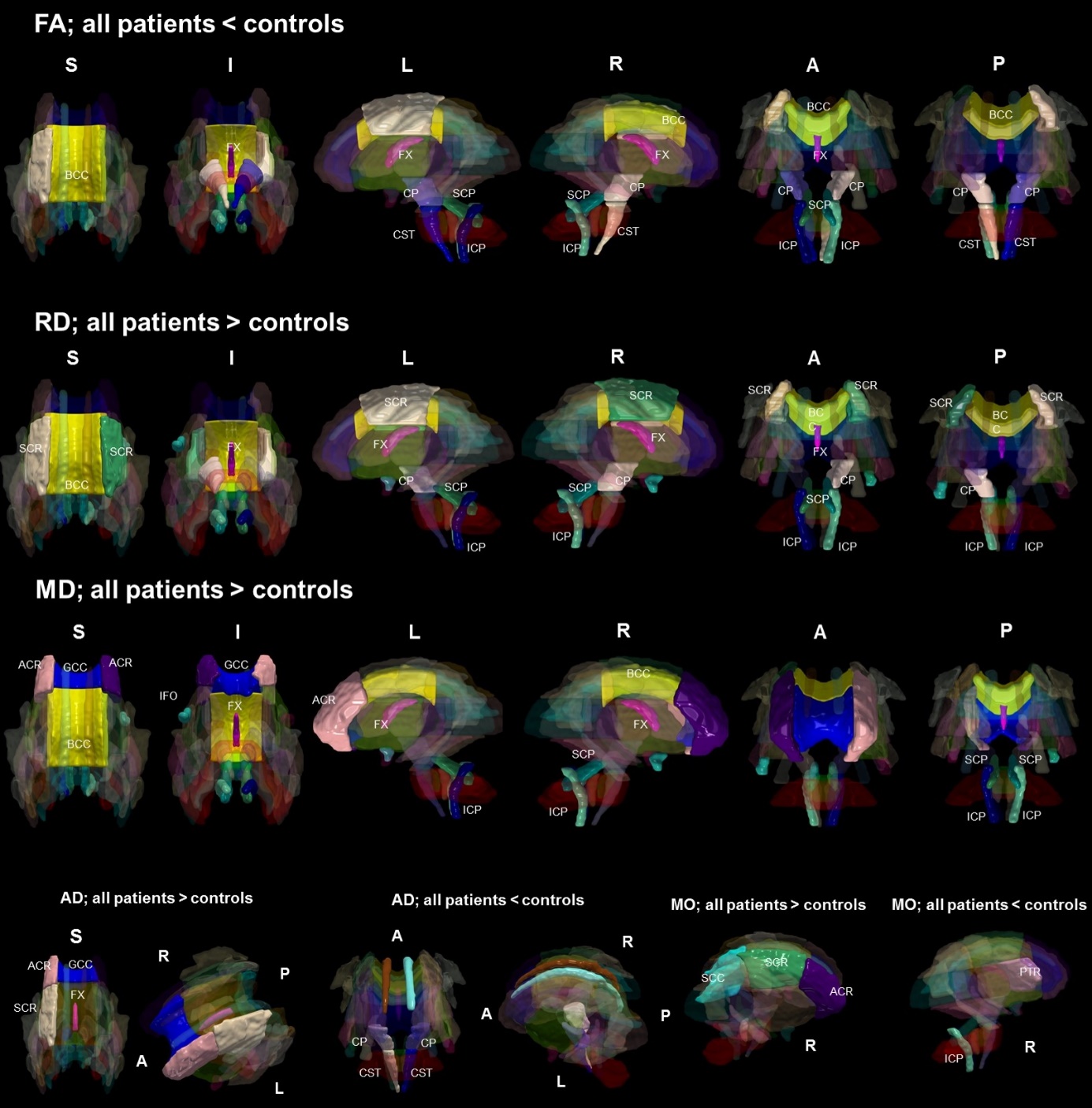


**Supplementary table 2. The result of the volume of interest analysis: the comparison between amyotrophic lateral sclerosis (ALS) and normal controls (NCs)**

| FA | | | | | | | |
| --- | --- | --- | --- | --- | --- | --- | --- |
|  | **ALS (n=96)** | | **NCs (N=47)** | |  |  |  |
|  | **Mean** | **SD** | **Mean** | **SD** | ***p*-value** | ***q*-value** |  |
| **FA-AVR** | **0.45993** | **0.01930** | **0.46754** | **0.01676** | **0.00100** | **0.00851** | **TRUE** |
| FA-GCC | 0.58554 | 0.04241 | 0.58878 | 0.05875 | 0.22500 | 0.02872 | FALSE |
| **FA-BCC** | **0.64515** | **0.04123** | **0.67074** | **0.04151** | **0.00000** | **0.00213** | **TRUE** |
| FA-SCC | 0.75303 | 0.01992 | 0.75561 | 0.01804 | 0.29600 | 0.03404 | FALSE |
| **FA-FX** | **0.41519** | **0.08518** | **0.45405** | **0.10842** | **0.00000** | **0.00319** | **TRUE** |
| **FA-CST-R** | **0.51294** | **0.03856** | **0.54660** | **0.02990** | **0.00000** | **0.00426** | **TRUE** |
| FA-ML-R | 0.57221 | 0.02787 | 0.57630 | 0.02893 | 0.34300 | 0.03617 | FALSE |
| **FA-ICP-R** | **0.49649** | **0.02821** | **0.50961** | **0.02513** | **0.00500** | **0.01383** | **TRUE** |
| **FA-SCP-R** | **0.64408** | **0.03643** | **0.66294** | **0.02355** | **0.00300** | **0.01170** | **TRUE** |
| **FA-CP-R** | **0.62715** | **0.03773** | **0.65745** | **0.02912** | **0.00000** | **0.00106** | **TRUE** |
| FA-ALIC-R | 0.57967 | 0.03307 | 0.58414 | 0.02829 | 0.14800 | 0.02340 | FALSE |
| FA-PLIC-R | 0.64839 | 0.03347 | 0.65790 | 0.02091 | 0.07400 | 0.02021 | FALSE |
| FA-RLIC-R | 0.56883 | 0.03587 | 0.56750 | 0.03798 | 0.80900 | 0.04681 | FALSE |
| FA-ACR-R | 0.45471 | 0.03680 | 0.45722 | 0.03470 | 0.26100 | 0.03298 | FALSE |
| FA-SCR-R | 0.49415 | 0.02749 | 0.50194 | 0.02668 | 0.04500 | 0.01596 | FALSE |
| FA-PCR-R | 0.51734 | 0.03471 | 0.51739 | 0.02802 | 0.86600 | 0.04894 | FALSE |
| FA-PTR-R | 0.62281 | 0.03884 | 0.62826 | 0.03830 | 0.21300 | 0.02766 | FALSE |
| FA-SS-R | 0.53276 | 0.02900 | 0.53823 | 0.03169 | 0.22500 | 0.02979 | FALSE |
| FA-EC-R | 0.42279 | 0.02421 | 0.42763 | 0.02639 | 0.04300 | 0.01489 | FALSE |
| FA-CGC-R | 0.57041 | 0.04155 | 0.57046 | 0.03584 | 0.68200 | 0.04362 | FALSE |
| FA-CGH-R | 0.55975 | 0.05627 | 0.57432 | 0.05156 | 0.12000 | 0.02234 | FALSE |
| **FA-FX/ST-R** | **0.54963** | **0.03961** | **0.56728** | **0.04621** | **0.00300** | **0.01277** | **TRUE** |
| FA-SLF-R | 0.47285 | 0.03056 | 0.46794 | 0.02112 | 0.37500 | 0.03723 | FALSE |
| FA-SFO-R | 0.50246 | 0.03980 | 0.50558 | 0.03297 | 0.29900 | 0.03511 | FALSE |
| FA-IFO-R | 0.47829 | 0.04547 | 0.48753 | 0.04263 | 0.16200 | 0.02553 | FALSE |
| FA-UNC-R | 0.57338 | 0.06153 | 0.58159 | 0.06690 | 0.24700 | 0.03085 | FALSE |
| **FA-CST-L** | **0.52462** | **0.04040** | **0.56130** | **0.03874** | **0.00000** | **0.00532** | **TRUE** |
| FA-ML-L | 0.57998 | 0.02996 | 0.58692 | 0.03123 | 0.25100 | 0.03191 | FALSE |
| **FA-ICP-L** | **0.50145** | **0.02461** | **0.51494** | **0.02589** | **0.00200** | **0.00957** | **TRUE** |
| **FA-SCP-L** | **0.63821** | **0.02980** | **0.66074** | **0.02611** | **0.00000** | **0.00638** | **TRUE** |
| **FA-CP-L** | **0.57380** | **0.02846** | **0.59318** | **0.02125** | **0.00000** | **0.00745** | **TRUE** |
| FA-ALIC-L | 0.58710 | 0.02899 | 0.59180 | 0.02210 | 0.15200 | 0.02447 | FALSE |
| FA-PLIC-L | 0.65161 | 0.02858 | 0.65428 | 0.02668 | 0.66100 | 0.04255 | FALSE |
| FA-RLIC-L | 0.55472 | 0.02756 | 0.55694 | 0.02482 | 0.47600 | 0.04043 | FALSE |
| FA-ACR-L | 0.45362 | 0.03359 | 0.45751 | 0.02658 | 0.17100 | 0.02660 | FALSE |
| **FA-SCR-L** | **0.49867** | **0.02930** | **0.51152** | **0.02479** | **0.00200** | **0.01064** | **TRUE** |
| FA-PCR-L | 0.44955 | 0.03242 | 0.45754 | 0.02887 | 0.06900 | 0.01915 | FALSE |
| FA-PTR-L | 0.60281 | 0.04214 | 0.60201 | 0.03372 | 0.80300 | 0.04574 | FALSE |
| FA-SS-L | 0.53316 | 0.03343 | 0.53290 | 0.03131 | 0.99800 | 0.05000 | FALSE |
| FA-EC-L | 0.45255 | 0.02664 | 0.45438 | 0.02196 | 0.37500 | 0.03830 | FALSE |
| FA-CGC-L | 0.61645 | 0.04215 | 0.61472 | 0.03137 | 0.85300 | 0.04787 | FALSE |
| FA-CGH-L | 0.54621 | 0.04786 | 0.52971 | 0.04491 | 0.06800 | 0.01809 | FALSE |
| FA-FX/ST-L | 0.54689 | 0.03544 | 0.55499 | 0.04019 | 0.04800 | 0.01702 | FALSE |
| FA-SLF-L | 0.45755 | 0.03228 | 0.45957 | 0.02270 | 0.73300 | 0.04468 | FALSE |
| FA-SFO-L | 0.47508 | 0.04168 | 0.47782 | 0.02978 | 0.41400 | 0.03936 | FALSE |
| FA-IFO-L | 0.45600 | 0.04899 | 0.45949 | 0.05094 | 0.54000 | 0.04149 | FALSE |
| FA-UNC-L | 0.57979 | 0.06136 | 0.59191 | 0.06005 | 0.09800 | 0.02128 | FALSE |

| AD | | | | | | | |
| --- | --- | --- | --- | --- | --- | --- | --- |
|  | **ALS (n=96)** | | **NCs (N=47)** | |  |  |  |
|  | **Mean** | **SD** | **Mean** | **SD** | ***p*-value** | ***q*-value** |  |
| AD-AVR | 0.00119 | 0.00002 | 0.00120 | 0.00002 | 0.40000 | 0.03404 | FALSE |
| **AD-GCC** | **0.00148** | **0.00007** | **0.00146** | **0.00006** | **0.00400** | **0.01064** | **TRUE** |
| AD-BCC | 0.00158 | 0.00004 | 0.00159 | 0.00004 | 0.08800 | 0.02234 | FALSE |
| AD-SCC | 0.00165 | 0.00005 | 0.00164 | 0.00007 | 0.51800 | 0.03723 | FALSE |
| **AD-FX** | **0.00242** | **0.00025** | **0.00232** | **0.00026** | **0.00100** | **0.00426** | **TRUE** |
| **AD-CST-R** | **0.00113** | **0.00006** | **0.00121** | **0.00006** | **0.00000** | **0.00106** | **TRUE** |
| AD-ML-R | 0.00128 | 0.00005 | 0.00127 | 0.00005 | 0.27900 | 0.02979 | FALSE |
| AD-ICP-R | 0.00119 | 0.00004 | 0.00119 | 0.00004 | 0.56500 | 0.04149 | FALSE |
| AD-SCP-R | 0.00158 | 0.00007 | 0.00158 | 0.00007 | 0.92700 | 0.04894 | FALSE |
| **AD-CP-R** | **0.00136** | **0.00008** | **0.00141** | **0.00005** | **0.00100** | **0.00532** | **TRUE** |
| AD-ALIC-R | 0.00127 | 0.00005 | 0.00126 | 0.00005 | 0.04900 | 0.01596 | FALSE |
| AD-PLIC-R | 0.00133 | 0.00005 | 0.00133 | 0.00004 | 0.45800 | 0.03617 | FALSE |
| AD-RLIC-R | 0.00128 | 0.00007 | 0.00128 | 0.00006 | 0.54600 | 0.03936 | FALSE |
| AD-ACR-R | 0.00121 | 0.00004 | 0.00120 | 0.00005 | 0.25100 | 0.02872 | FALSE |
| **AD-SCR-R** | **0.00119** | **0.00005** | **0.00117** | **0.00005** | **0.00300** | **0.00745** | **TRUE** |
| AD-PCR-R | 0.00127 | 0.00007 | 0.00127 | 0.00007 | 0.16300 | 0.02447 | FALSE |
| AD-PTR-R | 0.00140 | 0.00005 | 0.00142 | 0.00005 | 0.04600 | 0.01489 | FALSE |
| AD-SS-R | 0.00131 | 0.00006 | 0.00132 | 0.00006 | 0.54800 | 0.04043 | FALSE |
| AD-EC-R | 0.00115 | 0.00006 | 0.00115 | 0.00004 | 0.68300 | 0.04362 | FALSE |
| AD-CGC-R | 0.00119 | 0.00006 | 0.00120 | 0.00006 | 0.30200 | 0.03085 | FALSE |
| **AD-CGH-R** | **0.00105** | **0.00009** | **0.00110** | **0.00009** | **0.00300** | **0.00851** | **TRUE** |
| **AD-FX/ST-R** | **0.00125** | **0.00008** | **0.00129** | **0.00007** | **0.00300** | **0.00957** | **TRUE** |
| AD-SLF-R | 0.00117 | 0.00005 | 0.00116 | 0.00004 | 0.07100 | 0.01915 | FALSE |
| AD-SFO-R | 0.00116 | 0.00007 | 0.00114 | 0.00007 | 0.02400 | 0.01277 | FALSE |
| AD-IFO-R | 0.00121 | 0.00007 | 0.00119 | 0.00006 | 0.06000 | 0.01809 | FALSE |
| AD-UNC-R | 0.00154 | 0.00010 | 0.00153 | 0.00008 | 0.18700 | 0.02660 | FALSE |
| **AD-CST-L** | **0.00116** | **0.00007** | **0.00124** | **0.00007** | **0.00000** | **0.00213** | **TRUE** |
| AD-ML-L | 0.00131 | 0.00005 | 0.00129 | 0.00004 | 0.08200 | 0.02021 | FALSE |
| AD-ICP-L | 0.00122 | 0.00004 | 0.00120 | 0.00004 | 0.03600 | 0.01383 | FALSE |
| AD-SCP-L | 0.00157 | 0.00006 | 0.00157 | 0.00006 | 0.68300 | 0.04468 | FALSE |
| **AD-CP-L** | **0.00133** | **0.00006** | **0.00138** | **0.00005** | **0.00000** | **0.00319** | **TRUE** |
| AD-ALIC-L | 0.00127 | 0.00004 | 0.00126 | 0.00005 | 0.05900 | 0.01702 | FALSE |
| AD-PLIC-L | 0.00135 | 0.00004 | 0.00136 | 0.00005 | 0.66700 | 0.04255 | FALSE |
| AD-RLIC-L | 0.00132 | 0.00006 | 0.00133 | 0.00005 | 0.89900 | 0.04787 | FALSE |
| **AD-ACR-L** | **0.00120** | **0.00004** | **0.00118** | **0.00004** | **0.00200** | **0.00638** | **TRUE** |
| AD-SCR-L | 0.00123 | 0.00004 | 0.00123 | 0.00004 | 0.30600 | 0.03191 | FALSE |
| AD-PCR-L | 0.00123 | 0.00005 | 0.00123 | 0.00005 | 0.52700 | 0.03830 | FALSE |
| AD-PTR-L | 0.00142 | 0.00005 | 0.00142 | 0.00005 | 0.77600 | 0.04681 | FALSE |
| AD-SS-L | 0.00135 | 0.00006 | 0.00134 | 0.00005 | 0.11900 | 0.02340 | FALSE |
| AD-EC-L | 0.00122 | 0.00005 | 0.00121 | 0.00005 | 0.32200 | 0.03298 | FALSE |
| AD-CGC-L | 0.00125 | 0.00007 | 0.00126 | 0.00007 | 0.44900 | 0.03511 | FALSE |
| AD-CGH-L | 0.00111 | 0.00008 | 0.00113 | 0.00008 | 0.08600 | 0.02128 | FALSE |
| **AD-FX/ST-L** | **0.00129** | **0.00007** | **0.00132** | **0.00006** | **0.00400** | **0.01170** | **TRUE** |
| AD-SLF-L | 0.00114 | 0.00005 | 0.00114 | 0.00005 | 1.00000 | 0.05000 | FALSE |
| AD-SFO-L | 0.00111 | 0.00007 | 0.00111 | 0.00007 | 0.20700 | 0.02766 | FALSE |
| AD-IFO-L | 0.00121 | 0.00009 | 0.00121 | 0.00007 | 0.75500 | 0.04574 | FALSE |
| AD-UNC-L | 0.00155 | 0.00013 | 0.00159 | 0.00013 | 0.17700 | 0.02553 | FALSE |

| RD | | | | | | | |
| --- | --- | --- | --- | --- | --- | --- | --- |
|  | **ALS (n=96)** | | **NCs (N=47)** | |  |  |  |
|  | **Mean** | **SD** | **Mean** | **SD** | ***p*-value** | ***q*-value** |  |
| **RD-AVR** | **0.00056** | **0.00003** | **0.00055** | **0.00003** | **0.00800** | **0.01064** | **TRUE** |
| RD-GCC | 0.00051 | 0.00007 | 0.00050 | 0.00008 | 0.03000 | 0.01702 | FALSE |
| **RD-BCC** | **0.00046** | **0.00006** | **0.00043** | **0.00005** | **0.00000** | **0.00106** | **TRUE** |
| RD-SCC | 0.00035 | 0.00003 | 0.00035 | 0.00003 | 0.24300 | 0.03298 | FALSE |
| **RD-FX** | **0.00134** | **0.00033** | **0.00121** | **0.00038** | **0.00100** | **0.00532** | **TRUE** |
| RD-CST-R | 0.00051 | 0.00004 | 0.00050 | 0.00004 | 0.07600 | 0.02660 | FALSE |
| RD-ML-R | 0.00048 | 0.00003 | 0.00047 | 0.00003 | 0.01900 | 0.01277 | FALSE |
| **RD-ICP-R** | **0.00053** | **0.00003** | **0.00051** | **0.00002** | **0.00000** | **0.00213** | **TRUE** |
| **RD-SCP-R** | **0.00050** | **0.00005** | **0.00047** | **0.00004** | **0.00200** | **0.00745** | **TRUE** |
| **RD-CP-R** | **0.00045** | **0.00005** | **0.00042** | **0.00003** | **0.00100** | **0.00638** | **TRUE** |
| RD-ALIC-R | 0.00046 | 0.00004 | 0.00045 | 0.00003 | 0.05400 | 0.02340 | FALSE |
| RD-PLIC-R | 0.00041 | 0.00004 | 0.00039 | 0.00002 | 0.02600 | 0.01596 | FALSE |
| RD-RLIC-R | 0.00049 | 0.00005 | 0.00049 | 0.00004 | 0.87600 | 0.04894 | FALSE |
| RD-ACR-R | 0.00057 | 0.00005 | 0.00056 | 0.00005 | 0.16700 | 0.03085 | FALSE |
| **RD-SCR-R** | **0.00052** | **0.00003** | **0.00051** | **0.00003** | **0.00600** | **0.00851** | **TRUE** |
| RD-PCR-R | 0.00054 | 0.00005 | 0.00054 | 0.00004 | 0.52000 | 0.04149 | FALSE |
| RD-PTR-R | 0.00047 | 0.00005 | 0.00046 | 0.00005 | 0.26700 | 0.03511 | FALSE |
| RD-SS-R | 0.00055 | 0.00004 | 0.00054 | 0.00004 | 0.46100 | 0.03723 | FALSE |
| RD-EC-R | 0.00059 | 0.00005 | 0.00059 | 0.00004 | 0.11800 | 0.02872 | FALSE |
| RD-CGC-R | 0.00044 | 0.00004 | 0.00045 | 0.00003 | 0.79200 | 0.04574 | FALSE |
| RD-CGH-R | 0.00042 | 0.00006 | 0.00043 | 0.00005 | 0.83400 | 0.04681 | FALSE |
| RD-FX/ST-R | 0.00050 | 0.00005 | 0.00050 | 0.00005 | 0.08400 | 0.02766 | FALSE |
| RD-SLF-R | 0.00054 | 0.00004 | 0.00054 | 0.00003 | 1.00000 | 0.05000 | FALSE |
| RD-SFO-R | 0.00049 | 0.00006 | 0.00048 | 0.00004 | 0.05200 | 0.02021 | FALSE |
| **RD-IFO-R** | **0.00055** | **0.00006** | **0.00053** | **0.00005** | **0.01000** | **0.01170** | **TRUE** |
| RD-UNC-R | 0.00058 | 0.00010 | 0.00056 | 0.00010 | 0.07300 | 0.02553 | FALSE |
| RD-CST-L | 0.00050 | 0.00005 | 0.00049 | 0.00004 | 0.02400 | 0.01489 | FALSE |
| RD-ML-L | 0.00047 | 0.00003 | 0.00046 | 0.00003 | 0.05300 | 0.02234 | FALSE |
| **RD-ICP-L** | **0.00054** | **0.00003** | **0.00051** | **0.00002** | **0.00000** | **0.00319** | **TRUE** |
| **RD-SCP-L** | **0.00050** | **0.00004** | **0.00047** | **0.00004** | **0.00000** | **0.00426** | **TRUE** |
| RD-CP-L | 0.00049 | 0.00004 | 0.00048 | 0.00003 | 0.03500 | 0.01809 | FALSE |
| RD-ALIC-L | 0.00045 | 0.00004 | 0.00044 | 0.00003 | 0.03500 | 0.01915 | FALSE |
| RD-PLIC-L | 0.00041 | 0.00003 | 0.00041 | 0.00003 | 0.70300 | 0.04468 | FALSE |
| RD-RLIC-L | 0.00052 | 0.00003 | 0.00051 | 0.00003 | 0.44500 | 0.03617 | FALSE |
| RD-ACR-L | 0.00057 | 0.00005 | 0.00056 | 0.00004 | 0.02000 | 0.01383 | FALSE |
| **RD-SCR-L** | **0.00053** | **0.00004** | **0.00052** | **0.00003** | **0.00600** | **0.00957** | **TRUE** |
| RD-PCR-L | 0.00059 | 0.00005 | 0.00059 | 0.00004 | 0.16200 | 0.02979 | FALSE |
| RD-PTR-L | 0.00050 | 0.00005 | 0.00050 | 0.00004 | 0.49100 | 0.04043 | FALSE |
| RD-SS-L | 0.00056 | 0.00004 | 0.00055 | 0.00004 | 0.25900 | 0.03404 | FALSE |
| RD-EC-L | 0.00059 | 0.00004 | 0.00058 | 0.00003 | 0.05800 | 0.02447 | FALSE |
| RD-CGC-L | 0.00042 | 0.00004 | 0.00042 | 0.00003 | 0.69600 | 0.04362 | FALSE |
| RD-CGH-L | 0.00046 | 0.00005 | 0.00047 | 0.00004 | 0.05200 | 0.02128 | FALSE |
| RD-FX/ST-L | 0.00052 | 0.00004 | 0.00052 | 0.00005 | 0.85000 | 0.04787 | FALSE |
| RD-SLF-L | 0.00055 | 0.00004 | 0.00055 | 0.00003 | 0.65100 | 0.04255 | FALSE |
| RD-SFO-L | 0.00050 | 0.00006 | 0.00050 | 0.00004 | 0.19000 | 0.03191 | FALSE |
| RD-IFO-L | 0.00057 | 0.00006 | 0.00056 | 0.00006 | 0.48800 | 0.03936 | FALSE |
| RD-UNC-L | 0.00057 | 0.00010 | 0.00057 | 0.00011 | 0.47600 | 0.03830 | FALSE |

| MD | | | | | | | |
| --- | --- | --- | --- | --- | --- | --- | --- |
|  | **ALS (n=96)** | | **NCs (N=47)** | |  |  |  |
|  | **Mean** | **SD** | **Mean** | **SD** | ***p*-value** | ***q*-value** |  |
| MD-AVR | 0.00077 | 0.00003 | 0.00077 | 0.00002 | 0.08300 | 0.02234 | FALSE |
| **MD-GCC** | **0.00084** | **0.00006** | **0.00082** | **0.00006** | **0.00300** | **0.00745** | **TRUE** |
| **MD-BCC** | **0.00083** | **0.00004** | **0.00082** | **0.00004** | **0.00100** | **0.00319** | **TRUE** |
| MD-SCC | 0.00078 | 0.00003 | 0.00078 | 0.00004 | 0.27300 | 0.03404 | FALSE |
| **MD-FX** | **0.00170** | **0.00030** | **0.00158** | **0.00034** | **0.00100** | **0.00426** | **TRUE** |
| MD-CST-R | 0.00072 | 0.00004 | 0.00073 | 0.00004 | 0.03000 | 0.01702 | FALSE |
| MD-ML-R | 0.00074 | 0.00003 | 0.00073 | 0.00003 | 0.07900 | 0.02128 | FALSE |
| **MD-ICP-R** | **0.00075** | **0.00002** | **0.00074** | **0.00002** | **0.00200** | **0.00638** | **TRUE** |
| **MD-SCP-R** | **0.00086** | **0.00004** | **0.00084** | **0.00004** | **0.01000** | **0.01064** | **TRUE** |
| MD-CP-R | 0.00075 | 0.00005 | 0.00075 | 0.00003 | 0.78100 | 0.04574 | FALSE |
| MD-ALIC-R | 0.00073 | 0.00004 | 0.00072 | 0.00003 | 0.02100 | 0.01596 | FALSE |
| MD-PLIC-R | 0.00072 | 0.00003 | 0.00071 | 0.00002 | 0.03900 | 0.01915 | FALSE |
| MD-RLIC-R | 0.00075 | 0.00005 | 0.00075 | 0.00004 | 0.68600 | 0.04255 | FALSE |
| MD-ACR-R | 0.00078 | 0.00005 | 0.00078 | 0.00004 | 0.12900 | 0.02766 | FALSE |
| **MD-SCR-R** | **0.00075** | **0.00003** | **0.00073** | **0.00003** | **0.00100** | **0.00532** | **TRUE** |
| MD-PCR-R | 0.00078 | 0.00005 | 0.00078 | 0.00005 | 0.26500 | 0.03298 | FALSE |
| MD-PTR-R | 0.00078 | 0.00004 | 0.00078 | 0.00004 | 1.00000 | 0.04894 | FALSE |
| MD-SS-R | 0.00080 | 0.00004 | 0.00080 | 0.00004 | 0.83800 | 0.04681 | FALSE |
| MD-EC-R | 0.00078 | 0.00005 | 0.00077 | 0.00004 | 0.24500 | 0.03191 | FALSE |
| MD-CGC-R | 0.00069 | 0.00003 | 0.00070 | 0.00003 | 0.32700 | 0.03511 | FALSE |
| MD-CGH-R | 0.00063 | 0.00007 | 0.00065 | 0.00006 | 0.12700 | 0.02660 | FALSE |
| MD-FX/ST-R | 0.00075 | 0.00005 | 0.00076 | 0.00005 | 0.76800 | 0.04468 | FALSE |
| MD-SLF-R | 0.00075 | 0.00003 | 0.00075 | 0.00003 | 0.37000 | 0.03723 | FALSE |
| MD-SFO-R | 0.00072 | 0.00006 | 0.00070 | 0.00005 | 0.02000 | 0.01489 | FALSE |
| **MD-IFO-R** | **0.00077** | **0.00005** | **0.00075** | **0.00005** | **0.00600** | **0.00957** | **TRUE** |
| MD-UNC-R | 0.00090 | 0.00009 | 0.00088 | 0.00008 | 0.06100 | 0.02021 | FALSE |
| MD-CST-L | 0.00072 | 0.00005 | 0.00074 | 0.00003 | 0.09600 | 0.02447 | FALSE |
| MD-ML-L | 0.00075 | 0.00003 | 0.00074 | 0.00003 | 0.01900 | 0.01383 | FALSE |
| **MD-ICP-L** | **0.00076** | **0.00003** | **0.00074** | **0.00002** | **0.00000** | **0.00106** | **TRUE** |
| **MD-SCP-L** | **0.00086** | **0.00004** | **0.00083** | **0.00004** | **0.00000** | **0.00213** | **TRUE** |
| MD-CP-L | 0.00077 | 0.00004 | 0.00078 | 0.00003 | 0.34600 | 0.03617 | FALSE |
| **MD-ALIC-L** | **0.00072** | **0.00004** | **0.00072** | **0.00003** | **0.01300** | **0.01170** | **TRUE** |
| MD-PLIC-L | 0.00072 | 0.00002 | 0.00073 | 0.00002 | 1.00000 | 0.05000 | FALSE |
| MD-RLIC-L | 0.00079 | 0.00003 | 0.00078 | 0.00003 | 0.66900 | 0.04149 | FALSE |
| **MD-ACR-L** | **0.00078** | **0.00004** | **0.00077** | **0.00003** | **0.00400** | **0.00851** | **TRUE** |
| MD-SCR-L | 0.00076 | 0.00003 | 0.00075 | 0.00003 | 0.01300 | 0.01277 | FALSE |
| MD-PCR-L | 0.00081 | 0.00004 | 0.00080 | 0.00004 | 0.19100 | 0.03085 | FALSE |
| MD-PTR-L | 0.00081 | 0.00005 | 0.00080 | 0.00004 | 0.52600 | 0.04043 | FALSE |
| MD-SS-L | 0.00082 | 0.00004 | 0.00081 | 0.00003 | 0.14000 | 0.02872 | FALSE |
| MD-EC-L | 0.00080 | 0.00004 | 0.00079 | 0.00004 | 0.08600 | 0.02340 | FALSE |
| MD-CGC-L | 0.00069 | 0.00003 | 0.00070 | 0.00003 | 0.37500 | 0.03830 | FALSE |
| MD-CGH-L | 0.00067 | 0.00006 | 0.00069 | 0.00004 | 0.03300 | 0.01809 | FALSE |
| MD-FX/ST-L | 0.00077 | 0.00004 | 0.00078 | 0.00004 | 0.12300 | 0.02553 | FALSE |
| MD-SLF-L | 0.00074 | 0.00003 | 0.00075 | 0.00003 | 0.76700 | 0.04362 | FALSE |
| MD-SFO-L | 0.00070 | 0.00006 | 0.00070 | 0.00005 | 0.15400 | 0.02979 | FALSE |
| MD-IFO-L | 0.00078 | 0.00006 | 0.00078 | 0.00005 | 0.52100 | 0.03936 | FALSE |
| MD-UNC-L | 0.00090 | 0.00010 | 0.00091 | 0.00011 | 0.89700 | 0.04787 | FALSE |

| MO | | | | | | | |
| --- | --- | --- | --- | --- | --- | --- | --- |
|  | **ALS (n=96)** | | **NCs (N=47)** | |  |  |  |
|  | **Mean** | **SD** | **Mean** | **SD** | ***p*-value** | ***q*-value** |  |
| MO-AVR | 0.62652 | 0.00951 | 0.62420 | 0.00966 | 0.22600 | 0.02660 | FALSE |
| MO-GCC | 0.88575 | 0.04699 | 0.86969 | 0.07485 | 0.14000 | 0.02447 | FALSE |
| MO-BCC | 0.88495 | 0.03609 | 0.89156 | 0.03512 | 0.05000 | 0.01915 | FALSE |
| **MO-SCC** | **0.90665** | **0.02414** | **0.89652** | **0.03426** | **0.00700** | **0.00851** | **TRUE** |
| MO-FX | 0.81448 | 0.10829 | 0.80712 | 0.11069 | 0.92000 | 0.04894 | FALSE |
| MO-CST-R | 0.51876 | 0.08240 | 0.55506 | 0.08596 | 0.03300 | 0.01596 | FALSE |
| **MO-ML-R** | **0.73453** | **0.06659** | **0.70279** | **0.05903** | **0.00900** | **0.01064** | **TRUE** |
| **MO-ICP-R** | **0.54156** | **0.05496** | **0.56903** | **0.05575** | **0.00100** | **0.00319** | **TRUE** |
| MO-SCP-R | 0.80182 | 0.03930 | 0.80831 | 0.04042 | 0.53000 | 0.03723 | FALSE |
| MO-CP-R | 0.79136 | 0.03727 | 0.79431 | 0.03957 | 0.69000 | 0.04362 | FALSE |
| **MO-ALIC-R** | **0.79702** | **0.06269** | **0.75704** | **0.07192** | **0.00000** | **0.00106** | **TRUE** |
| MO-PLIC-R | 0.80843 | 0.05116 | 0.80038 | 0.04817 | 0.09500 | 0.02340 | FALSE |
| MO-RLIC-R | 0.66052 | 0.07530 | 0.64866 | 0.09107 | 0.26000 | 0.02979 | FALSE |
| **MO-ACR-R** | **0.75450** | **0.05189** | **0.72661** | **0.06471** | **0.00800** | **0.00957** | **TRUE** |
| **MO-SCR-R** | **0.65578** | **0.08665** | **0.61582** | **0.08591** | **0.00000** | **0.00213** | **TRUE** |
| MO-PCR-R | 0.61932 | 0.10533 | 0.59633 | 0.11321 | 0.06300 | 0.02021 | FALSE |
| **MO-PTR-R** | **0.79877** | **0.04994** | **0.82062** | **0.05656** | **0.00600** | **0.00638** | **TRUE** |
| MO-SS-R | 0.64927 | 0.06844 | 0.63885 | 0.08882 | 0.33100 | 0.03298 | FALSE |
| MO-EC-R | 0.53405 | 0.06832 | 0.53113 | 0.06578 | 0.91900 | 0.04787 | FALSE |
| MO-CGC-R | 0.65818 | 0.08142 | 0.62550 | 0.09169 | 0.07100 | 0.02128 | FALSE |
| MO-CGH-R | 0.71699 | 0.09913 | 0.70700 | 0.09676 | 0.58100 | 0.04149 | FALSE |
| MO-FX/ST-R | 0.59398 | 0.09251 | 0.62293 | 0.08249 | 0.02100 | 0.01277 | FALSE |
| MO-SLF-R | 0.59236 | 0.05633 | 0.58485 | 0.03882 | 0.47100 | 0.03404 | FALSE |
| **MO-SFO-R** | **0.74494** | **0.09749** | **0.69646** | **0.11675** | **0.00200** | **0.00532** | **TRUE** |
| MO-IFO-R | 0.67478 | 0.12415 | 0.69187 | 0.12569 | 0.31700 | 0.03191 | FALSE |
| MO-UNC-R | 0.85044 | 0.07801 | 0.84470 | 0.08795 | 0.50100 | 0.03617 | FALSE |
| **MO-CST-L** | **0.53798** | **0.09362** | **0.59685** | **0.09593** | **0.00100** | **0.00426** | **TRUE** |
| **MO-ML-L** | **0.81982** | **0.05325** | **0.79031** | **0.06299** | **0.00600** | **0.00745** | **TRUE** |
| MO-ICP-L | 0.58680 | 0.05673 | 0.58793 | 0.04909 | 0.63700 | 0.04255 | FALSE |
| MO-SCP-L | 0.79927 | 0.04907 | 0.80573 | 0.03981 | 0.57000 | 0.03936 | FALSE |
| MO-CP-L | 0.75433 | 0.04394 | 0.75381 | 0.04454 | 0.90700 | 0.04681 | FALSE |
| MO-ALIC-L | 0.79542 | 0.06454 | 0.78970 | 0.07745 | 0.24000 | 0.02872 | FALSE |
| MO-PLIC-L | 0.79616 | 0.06061 | 0.79484 | 0.06326 | 0.55400 | 0.03830 | FALSE |
| MO-RLIC-L | 0.65397 | 0.06529 | 0.64912 | 0.06192 | 0.77600 | 0.04468 | FALSE |
| MO-ACR-L | 0.71967 | 0.05350 | 0.70941 | 0.05394 | 0.29200 | 0.03085 | FALSE |
| MO-SCR-L | 0.66687 | 0.07458 | 0.64521 | 0.07430 | 0.02900 | 0.01489 | FALSE |
| MO-PCR-L | 0.59668 | 0.07884 | 0.58169 | 0.07336 | 0.18600 | 0.02553 | FALSE |
| MO-PTR-L | 0.79410 | 0.04963 | 0.81051 | 0.05049 | 0.03300 | 0.01702 | FALSE |
| MO-SS-L | 0.67761 | 0.07046 | 0.68285 | 0.06585 | 0.95000 | 0.05000 | FALSE |
| MO-EC-L | 0.61068 | 0.05035 | 0.61780 | 0.05250 | 0.23800 | 0.02766 | FALSE |
| MO-CGC-L | 0.67104 | 0.09079 | 0.65533 | 0.08557 | 0.49700 | 0.03511 | FALSE |
| MO-CGH-L | 0.75540 | 0.07677 | 0.72096 | 0.08770 | 0.01300 | 0.01170 | FALSE |
| MO-FX/ST-L | 0.58815 | 0.08913 | 0.60577 | 0.10078 | 0.08300 | 0.02234 | FALSE |
| MO-SLF-L | 0.54898 | 0.06240 | 0.52997 | 0.05443 | 0.02800 | 0.01383 | FALSE |
| MO-SFO-L | 0.80554 | 0.08877 | 0.77982 | 0.11256 | 0.03300 | 0.01809 | FALSE |
| MO-IFO-L | 0.75194 | 0.12068 | 0.74375 | 0.11907 | 0.81000 | 0.04574 | FALSE |
| MO-UNC-L | 0.83021 | 0.11464 | 0.84339 | 0.09035 | 0.57200 | 0.04043 | FALSE |

**Supplementary Table 3. Partial correlation coefficient between ALSFRS-R score and DTI each region**

| Region | Side | FA | AD | RD | MD | MO |
| --- | --- | --- | --- | --- | --- | --- |
| GCC | - | .082 | -0.194 | -0.157 | -0.196 | -0.164 |
| BCC | - | **.297**** | -0.105 | **-0.331**** | **-0.329**** | 0.129 |
| SCC | - | .203 | -0.050 | **-0.237*** | -0.192 | 0.024 |
| FX | - | **.221*** | -0.076 | -0.175 | -0.148 | **0.216*** |
| CST | R | **.366***** | 0.068 | **-0.333**** | **-0.208*** | **0.257*** |
| ML | R | .196 | -0.156 | **-0.287**** | **-0.291**** | -0.110 |
| ICP | R | **.307**** | -0.080 | **-0.311**** | **-0.297**** | -0.015 |
| SCP | R | **.305**** | 0.072 | **-0.284**** | -0.199 | 0.172 |
| CP | R | **.384***** | 0.064 | **-0.324**** | -0.174 | 0.192 |
| ALIC | R | .019 | -0.126 | -0.047 | -0.091 | -0.132 |
| PLIC | R | .169 | 0.108 | -0.147 | -0.060 | 0.029 |
| RLIC | R | -.160 | 0.129 | 0.150 | 0.161 | 0.008 |
| ACR | R | .100 | -0.155 | -0.163 | -0.182 | 0.036 |
| SCR | R | .200 | **-0.228*** | **-0.297**** | **-0.321**** | -0.099 |
| PCR | R | .057 | -0.202 | -0.154 | **-0.206*** | -0.061 |
| PTR | R | .053 | 0.016 | -0.056 | -0.035 | 0.042 |
| SS | R | .167 | 0.016 | -0.130 | -0.073 | -0.124 |
| EC | R | **.261*** | -0.137 | **-0.259**** | **-0.220*** | -0.061 |
| CGC | R | .095 | 0.056 | -0.072 | -0.020 | -0.018 |
| CGH | R | .002 | -0.016 | -0.007 | -0.018 | 0.008 |
| FX/ST | R | .056 | 0.147 | -0.022 | 0.074 | 0.054 |
| SLF | R | .076 | -0.173 | -0.173 | **-0.215*** | -0.033 |
| SFO | R | -.007 | -0.041 | 0.005 | -0.013 | -0.123 |
| IFO | R | .032 | -0.159 | -0.109 | -0.153 | -0.155 |
| UNC | R | -.022 | -0.008 | 0.005 | 0.001 | -0.002 |
| CST | L | **.451***** | 0.092 | **-0.411***** | **-0.243**** | **0.268**** |
| ML | L | .081 | -0.085 | -0.149 | -0.150 | -0.004 |
| ICP | L | **.273**** | -0.096 | **-0.288**** | **-0.255**** | -0.119 |
| SCP | L | **.436***** | 0.138 | **-0.365***** | -0.187 | **0.251*** |
| CP | L | **.278**** | -0.058 | **-0.310**** | **-0.239*** | -0.022 |
| ALIC | L | .060 | -0.120 | -0.087 | -0.119 | -0.169 |
| PLIC | L | .075 | -0.130 | -0.105 | -0.162 | -0.179 |
| RLIC | L | .131 | 0.075 | -0.061 | 0.000 | -0.088 |
| ACR | L | .031 | **-0.224*** | -0.126 | -0.175 | -0.085 |
| SCR | L | .086 | **-0.257*** | -0.171 | **-0.243*** | **-0.265*** |
| PCR | L | .080 | -0.187 | -0.118 | -0.166 | -0.135 |
| PTR | L | .073 | 0.033 | -0.084 | -0.054 | 0.183 |
| SS | L | .077 | -0.006 | -0.102 | -0.078 | 0.047 |
| EC | L | .113 | -0.083 | -0.130 | -0.126 | 0.009 |
| CGC | L | .056 | -0.020 | -0.057 | -0.060 | -0.060 |
| CGH | L | .031 | -0.029 | -0.050 | -0.052 | 0.090 |
| FX/ST | L | **.324**** | **0.267*** | -0.179 | 0.035 | **0.280**** |
| SLF | L | .126 | -0.037 | -0.115 | -0.110 | 0.010 |
| SFO | L | -.017 | -0.021 | 0.023 | 0.007 | -0.122 |
| IFO | L | .046 | -0.192 | -0.162 | **-0.209*** | -0.034 |
| UNC | L | .018 | -0.038 | -0.057 | -0.055 | 0.068 |

Adjusted for Age at DTI, gender, disease duration

* p<0.05, ** p<0.01, *** p<0.001

Abbreviation: FA, fractional anisotropy; AD, axial diffusivity; MD, mean diffusivity; RD, radial diffusivity; Mo, Mode; R, right; L, left.

†DTI regions were summarized in supplementary figure 1

**Supplementary Table 4. Partial correlation coefficient between delta ALSFRS-R from onset and DTI each region**

| Region | Side | FA | AD | RD | MD | MO |
| --- | --- | --- | --- | --- | --- | --- |
| GCC | - | 0.043 | 0.113 | 0.022 | 0.065 | 0.042 |
| BCC | - | **-0.216*** | 0.072 | **0.244*** | **0.240*** | -0.096 |
| SCC | - | -0.081 | -0.041 | 0.085 | 0.034 | -0.152 |
| FX | - | -0.158 | 0.010 | 0.091 | 0.069 | -0.093 |
| CST | R | -0.132 | -0.111 | 0.112 | 0.023 | -0.211 |
| ML | R | -0.120 | -0.058 | 0.111 | 0.049 | **0.036*** |
| ICP | R | -0.169 | **0.209*** | **0.215*** | **0.275**** | 0.113 |
| SCP | R | -0.074 | -0.183 | 0.014 | -0.080 | -0.124 |
| CP | R | -0.150 | -0.174 | 0.067 | -0.048 | -0.198 |
| ALIC | R | -0.029 | -0.003 | 0.024 | 0.016 | 0.033 |
| PLIC | R | -0.132 | -0.037 | 0.125 | 0.079 | -0.036 |
| RLIC | R | 0.136 | -0.021 | -0.110 | -0.080 | 0.035 |
| ACR | R | 0.057 | 0.149 | 0.039 | 0.085 | -0.100 |
| SCR | R | -0.080 | 0.201 | 0.183 | **0.226*** | 0.036 |
| PCR | R | 0.001 | 0.147 | 0.084 | 0.129 | -0.004 |
| PTR | R | 0.016 | -0.046 | -0.027 | -0.036 | -0.114 |
| SS | R | -0.079 | -0.037 | 0.038 | 0.005 | -0.063 |
| EC | R | -0.086 | -0.015 | 0.037 | 0.016 | 0.154 |
| CGC | R | -0.055 | -0.060 | 0.037 | -0.013 | 0.057 |
| CGH | R | -0.066 | -0.098 | -0.024 | -0.049 | -0.041 |
| FX/ST | R | 0.081 | -0.001 | -0.068 | -0.047 | 0.075 |
| SLF | R | -0.181 | 0.122 | **0.280**** | **0.269**** | -0.017 |
| SFO | R | 0.029 | -0.068 | -0.041 | -0.057 | 0.010 |
| IFO | R | -0.105 | 0.079 | 0.153 | 0.148 | -0.027 |
| UNC | R | 0.138 | -0.126 | -0.159 | -0.166 | 0.044 |
| CST | L | **-0.265*** | -0.143 | **0.230*** | 0.090 | -0.196 |
| ML | L | -0.045 | -0.108 | -0.011 | -0.067 | -0.038 |
| ICP | L | -0.160 | 0.113 | **0.213*** | **0.208*** | 0.000 |
| SCP | L | -0.172 | -0.111 | 0.106 | 0.013 | -0.093 |
| CP | L | -0.039 | -0.133 | -0.007 | -0.078 | 0.042 |
| ALIC | L | -0.076 | -0.068 | 0.053 | 0.008 | 0.047 |
| PLIC | L | -0.095 | -0.085 | 0.048 | -0.012 | 0.113 |
| RLIC | L | -0.026 | 0.056 | 0.065 | 0.073 | 0.010 |
| ACR | L | 0.037 | **0.229*** | 0.077 | 0.140 | 0.056 |
| SCR | L | -0.120 | 0.197 | 0.183 | **0.225*** | 0.115 |
| PCR | L | -0.004 | 0.096 | 0.039 | 0.069 | 0.086 |
| PTR | L | -0.036 | -0.027 | 0.017 | 0.005 | -0.011 |
| SS | L | -0.031 | 0.156 | 0.109 | 0.160 | -0.034 |
| EC | L | -0.030 | 0.161 | 0.086 | 0.129 | 0.184 |
| CGC | L | -0.047 | -0.034 | 0.030 | -0.003 | -0.108 |
| CGH | L | -0.071 | 0.039 | 0.057 | 0.062 | -0.001 |
| FX/ST | L | -0.120 | 0.004 | 0.113 | 0.079 | -0.079 |
| SLF | L | -0.167 | -0.037 | 0.152 | 0.099 | -0.043 |
| SFO | L | 0.003 | -0.076 | -0.037 | -0.057 | -0.029 |
| IFO | L | -0.071 | 0.005 | 0.055 | 0.040 | 0.044 |
| UNC | L | 0.127 | **-0.221*** | -0.171 | **-0.216*** | -0.097 |

Adjusted for Age at DTI, gender, disease duration

* p<0.05, ** p<0.01, *** p<0.001

Abbreviation: ALSFRS-R, amyotrophic lateral sclerosis functional rating scale-revised; FA, fractional anisotropy; AD, axial diffusivity; MD, mean diffusivity; RD, radial diffusivity; Mo, Mode; R, right; L, left.

†DTI regions were summarized in supplementary figure 2

**Supplementary Table 5. Subgroup analysis according to the symptom onset regions.**

| FA | | | | | | | | | | | | |
| --- | --- | --- | --- | --- | --- | --- | --- | --- | --- | --- | --- | --- |
|  | **Bulbar-onset**  **(n=19)** | | **Upper- limb-onset**  **(n=35)** | | **Lower-limb-onset**  **(n=23)** | | **Normal Controls (NCs)**  **(n=47)** | | ***Bulbar vs NCs*** | ***Upper-limb vs NCs*** | ***Lower-limb vs NCs*** |  |
|  | **Mean** | **SD** | **Mean** | **SD** | **Mean** | **SD** | **Mean** | **SD** | ***p*** | ***p*** | ***p*** |  |
| GCC | 0.56465 | 0.04602 | 0.59631 | 0.04452 | 0.58856 | 0.02929 | 0.58878 | 0.05875 | 0.2581 | 0.4552 | 0.3734 |  |
| BCC | **0.62557** | **0.04897** | **0.65023** | **0.03868** | **0.65282** | **0.03209** | **0.67074** | **0.04151** | **0.0002** | **0.0001** | **0.0038** |  |
| SCC | 0.74899 | 0.02387 | 0.75467 | 0.01840 | 0.75539 | 0.01853 | 0.75561 | 0.01804 | 0.4436 | 0.3935 | 0.8531 |  |
| FX | **0.38075** | **0.07287** | 0.43416 | 0.07407 | **0.41514** | **0.08317** | **0.45405** | **0.10842** | **0.0059** | 0.0140 | **0.0034** |  |
| CST-R | **0.51370** | **0.04867** | **0.51288** | **0.03432** | **0.50569** | **0.03725** | **0.54660** | **0.02990** | **0.0007** | **0.0001** | **0.0000** |  |
| CST-L | **0.51950** | **0.04127** | **0.52770** | **0.04202** | **0.52112** | **0.04216** | **0.56130** | **0.03874** | **0.0001** | **0.0000** | **0.0002** |  |
| ML-R | 0.56385 | 0.03284 | 0.57518 | 0.02906 | 0.57147 | 0.02442 | 0.57630 | 0.02893 | 0.1621 | 0.5750 | 0.4908 |  |
| ML-L | 0.57431 | 0.03090 | 0.57895 | 0.03046 | 0.58455 | 0.03015 | 0.58692 | 0.03123 | 0.1155 | 0.4282 | 0.8038 |  |
| ICP-R | 0.49540 | 0.02806 | 0.49838 | 0.02830 | **0.49073** | **0.02757** | **0.50961** | **0.02513** | 0.0512 | 0.0318 | **0.0040** |  |
| ICP-L | 0.50090 | 0.02917 | 0.50495 | 0.02839 | **0.49487** | **0.01836** | **0.51494** | **0.02589** | 0.0768 | 0.0464 | **0.0026** |  |
| SCP-R | 0.65293 | 0.02607 | 0.64622 | 0.03797 | 0.64527 | 0.04486 | 0.66294 | 0.02355 | 0.1941 | 0.0756 | 0.0321 |  |
| SCP-L | 0.65119 | 0.01789 | **0.63583** | **0.03386** | **0.63531** | **0.03407** | **0.66074** | **0.02611** | 0.1691 | **0.0017** | **0.0010** |  |
| CP-R | **0.62172** | **0.03090** | **0.62392** | **0.03944** | **0.63075** | **0.04224** | **0.65745** | **0.02912** | **0.0009** | **0.0000** | **0.0018** |  |
| CP-L | **0.56944** | **0.03269** | **0.57213** | **0.02841** | **0.57532** | **0.02759** | **0.59318** | **0.02125** | **0.0025** | **0.0007** | **0.0046** |  |
| ALIC-R | 0.57035 | 0.03452 | 0.58377 | 0.03517 | 0.58088 | 0.03169 | 0.58414 | 0.02829 | 0.4032 | 0.2448 | 0.3662 |  |
| ALIC-L | 0.57812 | 0.02983 | 0.59135 | 0.03079 | 0.58625 | 0.02724 | 0.59180 | 0.02210 | 0.2198 | 0.2324 | 0.1435 |  |
| PLIC-R | 0.65123 | 0.03128 | 0.64347 | 0.03720 | 0.64479 | 0.03099 | 0.65790 | 0.02091 | 0.6307 | 0.0160 | 0.0976 |  |
| PLIC-L | 0.64778 | 0.02842 | 0.64849 | 0.02830 | 0.65442 | 0.02670 | 0.65428 | 0.02668 | 0.4781 | 0.3826 | 0.8496 |  |
| RLIC-R | 0.56748 | 0.03263 | 0.57013 | 0.03999 | 0.57154 | 0.03623 | 0.56750 | 0.03798 | 0.8229 | 0.8569 | 0.7627 |  |
| RLIC-L | 0.55743 | 0.03064 | 0.55265 | 0.02370 | 0.55712 | 0.02944 | 0.55694 | 0.02482 | 0.8231 | 0.1998 | 0.7248 |  |
| ACR-R | 0.43827 | 0.04231 | 0.46537 | 0.03813 | 0.45773 | 0.02440 | 0.45722 | 0.03470 | 0.2313 | 0.9584 | 0.5898 |  |
| ACR-L | 0.43880 | 0.04012 | 0.46231 | 0.03092 | 0.46130 | 0.02700 | 0.45751 | 0.02658 | 0.1011 | 0.9110 | 0.9896 |  |
| SCR-R | 0.49101 | 0.02972 | 0.49146 | 0.02960 | 0.49633 | 0.02381 | 0.50194 | 0.02668 | 0.3221 | 0.0168 | 0.2146 |  |
| SCR-L | 0.49172 | 0.03050 | **0.49823** | **0.02982** | 0.50103 | 0.02590 | **0.51152** | **0.02479** | 0.0365 | **0.0047** | 0.0388 |  |
| PCR-R | 0.51601 | 0.04416 | 0.51548 | 0.03125 | 0.52118 | 0.02913 | 0.51739 | 0.02802 | 0.8930 | 0.4864 | 0.7803 |  |
| PCR-L | 0.44816 | 0.03690 | 0.44616 | 0.02814 | 0.45439 | 0.02467 | 0.45754 | 0.02887 | 0.3583 | 0.0116 | 0.5579 |  |
| PTR-R | 0.61962 | 0.03500 | 0.62676 | 0.04094 | 0.62124 | 0.04091 | 0.62826 | 0.03830 | 0.8946 | 0.5225 | 0.2488 |  |
| PTR-L | 0.60383 | 0.05017 | 0.60446 | 0.04155 | 0.60459 | 0.04102 | 0.60201 | 0.03372 | 0.4915 | 0.7651 | 0.8245 |  |
| SS-R | 0.52959 | 0.02662 | 0.53327 | 0.02890 | 0.53368 | 0.03264 | 0.53823 | 0.03169 | 0.3639 | 0.3185 | 0.6490 |  |
| SS-L | 0.54462 | 0.03134 | 0.53244 | 0.03506 | 0.52776 | 0.03381 | 0.53290 | 0.03131 | 0.1777 | 0.8516 | 0.5234 |  |
| EC-R | 0.41570 | 0.02956 | 0.42797 | 0.02428 | 0.42368 | 0.01900 | 0.42763 | 0.02639 | 0.2873 | 0.1753 | 0.0805 |  |
| EC-L | 0.44552 | 0.03257 | 0.45313 | 0.02422 | 0.45831 | 0.02644 | 0.45438 | 0.02196 | 0.4772 | 0.3444 | 0.8555 |  |
| CGC-R | 0.55699 | 0.04425 | 0.57046 | 0.04794 | 0.57717 | 0.03272 | 0.57046 | 0.03584 | 0.5325 | 0.3395 | 0.7385 |  |
| CGC-L | 0.61271 | 0.04500 | 0.62429 | 0.04551 | 0.61193 | 0.03296 | 0.61472 | 0.03137 | 0.6004 | 0.9816 | 0.2599 |  |
| CGH-R | 0.56809 | 0.06799 | **0.54013** | **0.05831** | 0.57901 | 0.04999 | **0.57432** | **0.05156** | 0.7275 | **0.0025** | 0.8480 |  |
| CGH-L | 0.55566 | 0.07014 | 0.54725 | 0.04709 | 0.54620 | 0.03682 | 0.52971 | 0.04491 | 0.0541 | 0.2549 | 0.2260 |  |
| FX/ST-R | 0.53423 | 0.03303 | 0.55435 | 0.03470 | 0.56001 | 0.04806 | 0.56728 | 0.04621 | 0.0248 | 0.0376 | 0.0405 |  |
| FX/ST-L | 0.54849 | 0.03778 | 0.54556 | 0.03455 | 0.55439 | 0.02589 | 0.55499 | 0.04019 | 0.6463 | 0.0124 | 0.1515 |  |
| SLF-R | 0.46940 | 0.02892 | 0.46915 | 0.03240 | 0.47845 | 0.02690 | 0.46794 | 0.02112 | 0.7029 | 0.9436 | 0.1715 |  |
| SLF-L | 0.45724 | 0.03163 | 0.44751 | 0.03031 | 0.46620 | 0.03199 | 0.45957 | 0.02270 | 0.7763 | 0.1080 | 0.3820 |  |
| SFO-R | 0.48239 | 0.03787 | 0.51336 | 0.04262 | 0.49997 | 0.03812 | 0.50558 | 0.03297 | 0.0967 | 0.8142 | 0.2997 |  |
| SFO-L | 0.46209 | 0.03828 | 0.48346 | 0.04684 | 0.47894 | 0.04496 | 0.47782 | 0.02978 | 0.3485 | 0.5393 | 0.5503 |  |
| IFO-R | 0.47985 | 0.05198 | 0.48033 | 0.05169 | 0.47449 | 0.03850 | 0.48753 | 0.04263 | 0.9736 | 0.2452 | 0.1603 |  |
| IFO-L | 0.43577 | 0.05800 | 0.45611 | 0.04940 | 0.46960 | 0.04281 | 0.45949 | 0.05094 | 0.2485 | 0.5522 | 0.7051 |  |
| UNC-R | 0.56975 | 0.06339 | 0.57935 | 0.06520 | 0.57884 | 0.06282 | 0.58159 | 0.06690 | 0.8376 | 0.4005 | 0.7152 |  |
| UNC-L | 0.56775 | 0.07047 | 0.58961 | 0.05662 | 0.58416 | 0.06989 | 0.59191 | 0.06005 | 0.4563 | 0.4509 | 0.7595 |  |

| AD | | | | | | | | | | | | |
| --- | --- | --- | --- | --- | --- | --- | --- | --- | --- | --- | --- | --- |
|  | **Bulbar-onset**  **(n=19)** | | **Upper- limb-onset**  **(n=35)** | | **Lower-limb-onset**  **(n=23)** | | **Normal Controls (NCs)**  **(n=47)** | | ***Bulbar vs NCs*** | ***Upper-limb vs NCs*** | ***Lower-limb vs NCs*** |  |
|  | **Mean** | **SD** | **Mean** | **SD** | **Mean** | **SD** | **Mean** | **SD** | ***p*** | ***p*** | ***p*** |  |
| GCC | 0.00150 | 0.00007 | 0.00148 | 0.00007 | 0.00146 | 0.00006 | 0.00146 | 0.00006 | 0.0886 | 0.0191 | 0.8667 |  |
| BCC | 0.00157 | 0.00004 | 0.00157 | 0.00004 | 0.00158 | 0.00006 | 0.00159 | 0.00004 | 0.0776 | 0.0997 | 0.1497 |  |
| SCC | 0.00164 | 0.00005 | 0.00164 | 0.00006 | 0.00165 | 0.00004 | 0.00164 | 0.00007 | 0.8965 | 0.9205 | 0.3762 |  |
| FX | 0.00249 | 0.00023 | 0.00237 | 0.00019 | 0.00241 | 0.00023 | 0.00232 | 0.00026 | 0.0306 | 0.0296 | 0.0230 |  |
| CST-R | **0.00115** | **0.00006** | **0.00113** | **0.00006** | **0.00112** | **0.00004** | **0.00121** | **0.00006** | **0.0000** | **0.0000** | **0.0000** |  |
| CST-L | **0.00117** | **0.00008** | **0.00116** | **0.00007** | **0.00115** | **0.00006** | **0.00124** | **0.00007** | **0.0000** | **0.0000** | **0.0000** |  |
| ML-R | 0.00127 | 0.00005 | 0.00129 | 0.00005 | 0.00128 | 0.00004 | 0.00127 | 0.00005 | 0.6317 | 0.1145 | 0.7558 |  |
| ML-L | 0.00129 | 0.00005 | 0.00132 | 0.00005 | 0.00130 | 0.00005 | 0.00129 | 0.00004 | 0.7328 | 0.0126 | 0.3238 |  |
| ICP-R | 0.00118 | 0.00004 | 0.00121 | 0.00003 | 0.00119 | 0.00003 | 0.00119 | 0.00004 | 0.4462 | 0.5277 | 0.1783 |  |
| ICP-L | 0.00120 | 0.00004 | **0.00124** | **0.00003** | 0.00122 | 0.00003 | **0.00120** | **0.00004** | 0.4304 | **0.0054** | 0.6194 |  |
| SCP-R | 0.00159 | 0.00009 | 0.00158 | 0.00006 | 0.00157 | 0.00005 | 0.00158 | 0.00007 | 0.7209 | 0.4654 | 0.9782 |  |
| SCP-L | 0.00158 | 0.00007 | 0.00156 | 0.00007 | 0.00156 | 0.00005 | 0.00157 | 0.00006 | 0.8049 | 0.7619 | 0.7910 |  |
| CP-R | 0.00137 | 0.00006 | 0.00137 | 0.00008 | **0.00135** | **0.00009** | **0.00141** | **0.00005** | 0.0171 | 0.0102 | **0.0030** |  |
| CP-L | **0.00132** | **0.00005** | **0.00134** | **0.00007** | **0.00133** | **0.00005** | **0.00138** | **0.00005** | **0.0006** | **0.0042** | **0.0039** |  |
| ALIC-R | 0.00129 | 0.00006 | 0.00127 | 0.00004 | 0.00126 | 0.00005 | 0.00126 | 0.00005 | 0.2865 | 0.1059 | 0.8330 |  |
| ALIC-L | 0.00129 | 0.00007 | 0.00126 | 0.00003 | 0.00127 | 0.00003 | 0.00126 | 0.00005 | 0.6520 | 0.7678 | 0.1696 |  |
| PLIC-R | 0.00134 | 0.00007 | 0.00133 | 0.00005 | 0.00133 | 0.00004 | 0.00133 | 0.00004 | 0.8594 | 0.5751 | 0.8503 |  |
| PLIC-L | 0.00135 | 0.00005 | 0.00134 | 0.00005 | 0.00137 | 0.00004 | 0.00136 | 0.00005 | 0.1043 | 0.2135 | 0.2342 |  |
| RLIC-R | 0.00129 | 0.00007 | 0.00126 | 0.00009 | 0.00129 | 0.00007 | 0.00128 | 0.00006 | 0.8068 | 0.7859 | 0.3677 |  |
| RLIC-L | 0.00132 | 0.00004 | 0.00132 | 0.00006 | 0.00133 | 0.00006 | 0.00133 | 0.00005 | 0.6456 | 0.9066 | 0.9248 |  |
| ACR-R | 0.00122 | 0.00005 | 0.00120 | 0.00004 | 0.00119 | 0.00004 | 0.00120 | 0.00005 | 0.3249 | 0.4578 | 0.7497 |  |
| ACR-L | 0.00121 | 0.00004 | 0.00120 | 0.00004 | 0.00120 | 0.00003 | 0.00118 | 0.00004 | 0.1989 | 0.0173 | 0.1110 |  |
| SCR-R | 0.00120 | 0.00005 | 0.00119 | 0.00004 | 0.00119 | 0.00005 | 0.00117 | 0.00005 | 0.1077 | 0.0237 | 0.1667 |  |
| SCR-L | 0.00123 | 0.00005 | 0.00122 | 0.00003 | 0.00123 | 0.00004 | 0.00123 | 0.00004 | 0.9059 | 0.9360 | 0.3962 |  |
| PCR-R | 0.00129 | 0.00007 | 0.00125 | 0.00006 | 0.00128 | 0.00007 | 0.00127 | 0.00007 | 0.5290 | 0.8609 | 0.0857 |  |
| PCR-L | 0.00123 | 0.00006 | 0.00121 | 0.00004 | 0.00124 | 0.00004 | 0.00123 | 0.00005 | 0.9727 | 0.1838 | 0.1379 |  |
| PTR-R | 0.00141 | 0.00005 | 0.00140 | 0.00005 | 0.00140 | 0.00005 | 0.00142 | 0.00005 | 0.2899 | 0.0415 | 0.0809 |  |
| PTR-L | 0.00142 | 0.00005 | 0.00141 | 0.00004 | 0.00143 | 0.00005 | 0.00142 | 0.00005 | 0.6895 | 0.6096 | 0.8969 |  |
| SS-R | 0.00130 | 0.00007 | 0.00131 | 0.00007 | 0.00131 | 0.00005 | 0.00132 | 0.00006 | 0.1283 | 0.6815 | 0.3790 |  |
| SS-L | 0.00136 | 0.00005 | 0.00135 | 0.00006 | 0.00136 | 0.00006 | 0.00134 | 0.00005 | 0.4999 | 0.4960 | 0.6383 |  |
| EC-R | 0.00117 | 0.00008 | 0.00115 | 0.00006 | 0.00115 | 0.00006 | 0.00115 | 0.00004 | 0.8985 | 0.5654 | 0.5143 |  |
| EC-L | 0.00123 | 0.00006 | 0.00121 | 0.00005 | 0.00122 | 0.00005 | 0.00121 | 0.00005 | 0.6533 | 0.9468 | 0.5668 |  |
| CGC-R | 0.00119 | 0.00007 | 0.00119 | 0.00006 | 0.00119 | 0.00007 | 0.00120 | 0.00006 | 0.6110 | 0.2155 | 0.2695 |  |
| CGC-L | 0.00126 | 0.00007 | 0.00126 | 0.00007 | 0.00124 | 0.00008 | 0.00126 | 0.00007 | 0.9841 | 0.6419 | 0.1095 |  |
| CGH-R | 0.00104 | 0.00012 | 0.00105 | 0.00009 | 0.00104 | 0.00010 | 0.00110 | 0.00009 | 0.0091 | 0.0229 | 0.0174 |  |
| CGH-L | 0.00110 | 0.00007 | 0.00111 | 0.00008 | 0.00111 | 0.00009 | 0.00113 | 0.00008 | 0.3254 | 0.0849 | 0.1263 |  |
| FX/ST-R | 0.00126 | 0.00008 | 0.00123 | 0.00009 | 0.00126 | 0.00007 | 0.00129 | 0.00007 | 0.0580 | 0.0021 | 0.0464 |  |
| FX/ST-L | 0.00128 | 0.00005 | 0.00128 | 0.00008 | 0.00130 | 0.00006 | 0.00132 | 0.00006 | 0.0550 | 0.0004 | 0.0736 |  |
| SLF-R | 0.00117 | 0.00005 | 0.00117 | 0.00005 | 0.00118 | 0.00005 | 0.00116 | 0.00004 | 0.4259 | 0.3132 | 0.1397 |  |
| SLF-L | 0.00114 | 0.00005 | 0.00113 | 0.00005 | 0.00115 | 0.00004 | 0.00114 | 0.00005 | 0.5404 | 0.3416 | 0.5104 |  |
| SFO-R | 0.00117 | 0.00007 | 0.00116 | 0.00007 | 0.00115 | 0.00007 | 0.00114 | 0.00007 | 0.7605 | 0.0414 | 0.2843 |  |
| SFO-L | 0.00113 | 0.00009 | 0.00111 | 0.00005 | 0.00110 | 0.00006 | 0.00111 | 0.00007 | 0.7785 | 0.4480 | 0.8438 |  |
| IFO-R | 0.00120 | 0.00007 | 0.00122 | 0.00006 | 0.00121 | 0.00007 | 0.00119 | 0.00006 | 0.7009 | 0.0116 | 0.3648 |  |
| IFO-L | 0.00120 | 0.00011 | 0.00121 | 0.00009 | 0.00122 | 0.00008 | 0.00121 | 0.00007 | 0.3828 | 0.6746 | 0.7075 |  |
| UNC-R | 0.00154 | 0.00012 | 0.00154 | 0.00010 | 0.00155 | 0.00011 | 0.00153 | 0.00008 | 0.9556 | 0.1482 | 0.2769 |  |
| UNC-L | 0.00154 | 0.00019 | 0.00153 | 0.00012 | 0.00157 | 0.00011 | 0.00159 | 0.00013 | 0.0449 | 0.0468 | 0.4285 |  |

| RD | | | | | | | | | | | | |
| --- | --- | --- | --- | --- | --- | --- | --- | --- | --- | --- | --- | --- |
|  | **Bulbar-onset**  **(n=19)** | | **Upper- limb-onset**  **(n=35)** | | **Lower-limb-onset**  **(n=23)** | | **Normal Controls (NCs)**  **(n=47)** | | ***Bulbar vs NCs*** | ***Upper-limb vs NCs*** | ***Lower-limb vs NCs*** |  |
|  | **Mean** | **SD** | **Mean** | **SD** | **Mean** | **SD** | **Mean** | **SD** | ***p*** | ***p*** | ***p*** |  |
| GCC | 0.00055 | 0.00008 | 0.00050 | 0.00007 | 0.00050 | 0.00004 | 0.00050 | 0.00008 | 0.0835 | 0.1159 | 0.3749 |  |
| BCC | **0.00049** | **0.00007** | **0.00045** | **0.00005** | 0.00045 | 0.00004 | **0.00043** | **0.00005** | **0.0009** | **0.0007** | 0.0197 |  |
| SCC | 0.00035 | 0.00003 | 0.00035 | 0.00003 | 0.00035 | 0.00003 | 0.00035 | 0.00003 | 0.5205 | 0.4533 | 0.7199 |  |
| FX | 0.00146 | 0.00030 | 0.00126 | 0.00025 | 0.00133 | 0.00032 | 0.00121 | 0.00038 | 0.0125 | 0.0579 | 0.0186 |  |
| CST-R | 0.00051 | 0.00005 | 0.00051 | 0.00004 | 0.00051 | 0.00004 | 0.00050 | 0.00004 | 0.1892 | 0.1142 | 0.0881 |  |
| CST-L | 0.00051 | 0.00007 | 0.00050 | 0.00005 | 0.00049 | 0.00004 | 0.00049 | 0.00004 | 0.0666 | 0.0228 | 0.2275 |  |
| ML-R | 0.00048 | 0.00003 | 0.00048 | 0.00003 | 0.00047 | 0.00003 | 0.00047 | 0.00003 | 0.2703 | 0.1306 | 0.3964 |  |
| ML-L | 0.00047 | 0.00003 | 0.00048 | 0.00003 | 0.00046 | 0.00004 | 0.00046 | 0.00003 | 0.1593 | 0.0491 | 0.8951 |  |
| ICP-R | 0.00053 | 0.00003 | **0.00053** | **0.00003** | **0.00054** | **0.00003** | **0.00051** | **0.00002** | 0.0165 | **0.0004** | **0.0006** |  |
| ICP-L | 0.00053 | 0.00003 | **0.00054** | **0.00003** | **0.00054** | **0.00003** | **0.00051** | **0.00002** | 0.0054 | **0.0002** | **0.0001** |  |
| SCP-R | 0.00049 | 0.00003 | 0.00050 | 0.00005 | 0.00050 | 0.00006 | 0.00047 | 0.00004 | 0.2373 | 0.0278 | 0.0220 |  |
| SCP-L | 0.00049 | 0.00003 | **0.00050** | **0.00004** | **0.00050** | **0.00004** | **0.00047** | **0.00004** | 0.0941 | **0.0012** | **0.0004** |  |
| CP-R | 0.00045 | 0.00003 | **0.00045** | **0.00005** | 0.00044 | 0.00006 | **0.00042** | **0.00003** | 0.0255 | **0.0014** | 0.0575 |  |
| CP-L | 0.00050 | 0.00004 | 0.00050 | 0.00003 | 0.00049 | 0.00004 | 0.00048 | 0.00003 | 0.1186 | 0.0896 | 0.1287 |  |
| ALIC-R | 0.00047 | 0.00005 | 0.00045 | 0.00005 | 0.00045 | 0.00004 | 0.00045 | 0.00003 | 0.2673 | 0.1165 | 0.4872 |  |
| ALIC-L | 0.00046 | 0.00005 | 0.00044 | 0.00004 | 0.00045 | 0.00003 | 0.00044 | 0.00003 | 0.1512 | 0.2052 | 0.0695 |  |
| PLIC-R | 0.00041 | 0.00004 | **0.00041** | **0.00004** | 0.00041 | 0.00003 | **0.00039** | **0.00002** | 0.4883 | **0.0063** | 0.0801 |  |
| PLIC-L | 0.00041 | 0.00003 | 0.00041 | 0.00003 | 0.00041 | 0.00003 | 0.00041 | 0.00003 | 0.8379 | 0.6328 | 0.7714 |  |
| RLIC-R | 0.00049 | 0.00005 | 0.00048 | 0.00005 | 0.00049 | 0.00004 | 0.00049 | 0.00004 | 0.8214 | 0.8668 | 0.9504 |  |
| RLIC-L | 0.00051 | 0.00003 | 0.00052 | 0.00003 | 0.00051 | 0.00004 | 0.00051 | 0.00003 | 0.7157 | 0.2522 | 0.7986 |  |
| ACR-R | 0.00059 | 0.00007 | 0.00055 | 0.00005 | 0.00056 | 0.00003 | 0.00056 | 0.00005 | 0.1492 | 0.8680 | 0.7895 |  |
| ACR-L | 0.00059 | 0.00006 | 0.00056 | 0.00004 | 0.00056 | 0.00003 | 0.00056 | 0.00004 | 0.0714 | 0.2653 | 0.5232 |  |
| SCR-R | 0.00053 | 0.00004 | **0.00052** | **0.00004** | 0.00052 | 0.00003 | **0.00051** | **0.00003** | 0.1648 | **0.0071** | 0.1914 |  |
| SCR-L | 0.00054 | 0.00005 | 0.00053 | 0.00004 | 0.00053 | 0.00003 | 0.00052 | 0.00003 | 0.1439 | 0.0413 | 0.0882 |  |
| PCR-R | 0.00055 | 0.00006 | 0.00054 | 0.00004 | 0.00054 | 0.00004 | 0.00054 | 0.00004 | 0.9155 | 0.6343 | 0.6967 |  |
| PCR-L | 0.00060 | 0.00007 | 0.00059 | 0.00004 | 0.00059 | 0.00003 | 0.00059 | 0.00004 | 0.5983 | 0.2746 | 0.5357 |  |
| PTR-R | 0.00047 | 0.00005 | 0.00046 | 0.00005 | 0.00047 | 0.00005 | 0.00046 | 0.00005 | 0.8861 | 0.6982 | 0.3501 |  |
| PTR-L | 0.00050 | 0.00007 | 0.00049 | 0.00005 | 0.00050 | 0.00005 | 0.00050 | 0.00004 | 0.7130 | 0.7667 | 0.6546 |  |
| SS-R | 0.00055 | 0.00004 | 0.00054 | 0.00004 | 0.00054 | 0.00004 | 0.00054 | 0.00004 | 0.9332 | 0.5176 | 0.8886 |  |
| SS-L | 0.00055 | 0.00004 | 0.00055 | 0.00004 | 0.00056 | 0.00004 | 0.00055 | 0.00004 | 0.8623 | 0.4732 | 0.1882 |  |
| EC-R | 0.00061 | 0.00007 | 0.00058 | 0.00004 | 0.00059 | 0.00004 | 0.00059 | 0.00004 | 0.4697 | 0.1509 | 0.4727 |  |
| EC-L | 0.00060 | 0.00006 | 0.00058 | 0.00003 | 0.00058 | 0.00004 | 0.00058 | 0.00003 | 0.1700 | 0.3751 | 0.4204 |  |
| CGC-R | 0.00046 | 0.00004 | 0.00044 | 0.00004 | 0.00044 | 0.00003 | 0.00045 | 0.00003 | 0.7126 | 0.8333 | 0.2398 |  |
| CGC-L | 0.00042 | 0.00004 | 0.00041 | 0.00004 | 0.00042 | 0.00003 | 0.00042 | 0.00003 | 0.3935 | 0.7324 | 0.7672 |  |
| CGH-R | 0.00041 | 0.00007 | 0.00044 | 0.00006 | 0.00041 | 0.00005 | 0.00043 | 0.00005 | 0.2075 | 0.1628 | 0.1877 |  |
| CGH-L | 0.00045 | 0.00007 | 0.00046 | 0.00005 | 0.00045 | 0.00005 | 0.00047 | 0.00004 | 0.0678 | 0.1853 | 0.0815 |  |
| FX/ST-R | 0.00052 | 0.00005 | 0.00049 | 0.00005 | 0.00050 | 0.00005 | 0.00050 | 0.00005 | 0.2339 | 0.4828 | 0.1962 |  |
| FX/ST-L | 0.00052 | 0.00004 | 0.00051 | 0.00004 | 0.00051 | 0.00004 | 0.00052 | 0.00005 | 0.5960 | 0.9293 | 0.7819 |  |
| SLF-R | 0.00054 | 0.00004 | 0.00054 | 0.00004 | 0.00054 | 0.00003 | 0.00054 | 0.00003 | 0.9527 | 0.6997 | 0.5269 |  |
| SLF-L | 0.00055 | 0.00004 | 0.00055 | 0.00003 | 0.00054 | 0.00004 | 0.00055 | 0.00003 | 0.4746 | 0.8400 | 0.2914 |  |
| SFO-R | 0.00052 | 0.00006 | 0.00048 | 0.00007 | 0.00049 | 0.00005 | 0.00048 | 0.00004 | 0.1427 | 0.2345 | 0.2832 |  |
| SFO-L | 0.00052 | 0.00007 | 0.00049 | 0.00006 | 0.00049 | 0.00005 | 0.00050 | 0.00004 | 0.4948 | 0.3678 | 0.5941 |  |
| IFO-R | 0.00055 | 0.00006 | **0.00055** | **0.00006** | 0.00055 | 0.00005 | **0.00053** | **0.00005** | 0.6746 | **0.0080** | 0.0397 |  |
| IFO-L | 0.00058 | 0.00008 | 0.00057 | 0.00006 | 0.00055 | 0.00005 | 0.00056 | 0.00006 | 0.6817 | 0.3134 | 0.8190 |  |
| UNC-R | 0.00059 | 0.00012 | 0.00057 | 0.00010 | 0.00057 | 0.00011 | 0.00056 | 0.00010 | 0.7404 | 0.1511 | 0.3576 |  |
| UNC-L | 0.00059 | 0.00014 | 0.00055 | 0.00007 | 0.00058 | 0.00012 | 0.00057 | 0.00011 | 0.7426 | 0.7506 | 0.9432 |  |

| MD | | | | | | | | | | | | |
| --- | --- | --- | --- | --- | --- | --- | --- | --- | --- | --- | --- | --- |
|  | **Bulbar-onset**  **(n=19)** | | **Upper- limb-onset**  **(n=35)** | | **Lower-limb-onset**  **(n=23)** | | **Normal Controls (NCs)**  **(n=47)** | | ***Bulbar vs NCs*** | ***Upper-limb vs NCs*** | ***Lower-limb vs NCs*** |  |
|  | **Mean** | **SD** | **Mean** | **SD** | **Mean** | **SD** | **Mean** | **SD** | ***p*** | ***p*** | ***p*** |  |
| GCC | 0.00086 | 0.00007 | 0.00082 | 0.00006 | 0.00082 | 0.00004 | 0.00082 | 0.00006 | 0.0348 | 0.0235 | 0.4364 |  |
| BCC | 0.00085 | 0.00006 | 0.00083 | 0.00004 | 0.00083 | 0.00003 | 0.00082 | 0.00004 | 0.0258 | 0.0191 | 0.1394 |  |
| SCC | 0.00078 | 0.00003 | 0.00078 | 0.00003 | 0.00078 | 0.00002 | 0.00078 | 0.00004 | 0.7683 | 0.6223 | 0.4488 |  |
| FX | 0.00180 | 0.00027 | 0.00163 | 0.00022 | 0.00169 | 0.00029 | 0.00158 | 0.00034 | 0.0147 | 0.0464 | 0.0181 |  |
| CST-R | 0.00073 | 0.00004 | 0.00072 | 0.00004 | 0.00071 | 0.00003 | 0.00073 | 0.00004 | 0.1769 | 0.1080 | 0.2160 |  |
| CST-L | 0.00073 | 0.00007 | 0.00072 | 0.00005 | 0.00071 | 0.00003 | 0.00074 | 0.00003 | 0.2950 | 0.3678 | 0.1533 |  |
| ML-R | 0.00074 | 0.00003 | 0.00075 | 0.00003 | 0.00074 | 0.00003 | 0.00073 | 0.00003 | 0.5867 | 0.0456 | 0.4233 |  |
| ML-L | 0.00074 | 0.00003 | **0.00076** | **0.00003** | 0.00074 | 0.00004 | **0.00074** | **0.00003** | 0.3907 | **0.0043** | 0.5118 |  |
| ICP-R | 0.00074 | 0.00002 | **0.00076** | **0.00003** | 0.00075 | 0.00003 | **0.00074** | **0.00002** | 0.1118 | **0.0012** | 0.0284 |  |
| ICP-L | 0.00076 | 0.00002 | **0.00077** | **0.00003** | 0.00077 | 0.00003 | **0.00074** | **0.00002** | 0.0103 | **0.0000** | 0.0013 |  |
| SCP-R | 0.00086 | 0.00004 | 0.00086 | 0.00004 | 0.00085 | 0.00004 | 0.00084 | 0.00004 | 0.4621 | 0.0333 | 0.0662 |  |
| SCP-L | 0.00085 | 0.00004 | 0.00086 | 0.00004 | 0.00086 | 0.00003 | 0.00083 | 0.00004 | 0.1733 | 0.0099 | 0.0051 |  |
| CP-R | 0.00076 | 0.00003 | 0.00076 | 0.00005 | 0.00074 | 0.00006 | 0.00075 | 0.00003 | 0.7768 | 0.3867 | 0.7216 |  |
| CP-L | 0.00077 | 0.00003 | 0.00078 | 0.00004 | 0.00077 | 0.00004 | 0.00078 | 0.00003 | 0.3393 | 0.6006 | 0.5117 |  |
| ALIC-R | 0.00075 | 0.00005 | 0.00073 | 0.00004 | 0.00072 | 0.00003 | 0.00072 | 0.00003 | 0.1949 | 0.0599 | 0.5457 |  |
| ALIC-L | 0.00074 | 0.00005 | 0.00072 | 0.00003 | 0.00073 | 0.00002 | 0.00072 | 0.00003 | 0.2003 | 0.2814 | 0.0461 |  |
| PLIC-R | 0.00072 | 0.00004 | 0.00072 | 0.00004 | 0.00072 | 0.00002 | 0.00071 | 0.00002 | 0.5357 | 0.0205 | 0.1600 |  |
| PLIC-L | 0.00072 | 0.00003 | 0.00072 | 0.00002 | 0.00073 | 0.00002 | 0.00073 | 0.00002 | 0.3859 | 0.7036 | 0.3275 |  |
| RLIC-R | 0.00076 | 0.00005 | 0.00074 | 0.00006 | 0.00075 | 0.00004 | 0.00075 | 0.00004 | 0.8075 | 0.8050 | 0.6265 |  |
| RLIC-L | 0.00078 | 0.00002 | 0.00079 | 0.00004 | 0.00078 | 0.00004 | 0.00078 | 0.00003 | 0.6208 | 0.4983 | 0.8271 |  |
| ACR-R | 0.00080 | 0.00006 | 0.00077 | 0.00004 | 0.00077 | 0.00003 | 0.00078 | 0.00004 | 0.1327 | 0.6712 | 0.9372 |  |
| ACR-L | 0.00079 | 0.00005 | 0.00077 | 0.00004 | 0.00077 | 0.00003 | 0.00077 | 0.00003 | 0.0675 | 0.0856 | 0.2848 |  |
| SCR-R | 0.00075 | 0.00004 | **0.00074** | **0.00003** | 0.00074 | 0.00003 | **0.00073** | **0.00003** | 0.0770 | **0.0026** | 0.1091 |  |
| SCR-L | 0.00077 | 0.00004 | 0.00076 | 0.00003 | 0.00076 | 0.00003 | 0.00075 | 0.00003 | 0.3078 | 0.1428 | 0.1018 |  |
| PCR-R | 0.00079 | 0.00006 | 0.00077 | 0.00004 | 0.00079 | 0.00004 | 0.00078 | 0.00005 | 0.7144 | 0.8063 | 0.2860 |  |
| PCR-L | 0.00081 | 0.00006 | 0.00080 | 0.00004 | 0.00081 | 0.00003 | 0.00080 | 0.00004 | 0.7127 | 0.8022 | 0.2918 |  |
| PTR-R | 0.00078 | 0.00004 | 0.00077 | 0.00004 | 0.00078 | 0.00004 | 0.00078 | 0.00004 | 0.7508 | 0.5544 | 0.9927 |  |
| PTR-L | 0.00081 | 0.00006 | 0.00080 | 0.00004 | 0.00081 | 0.00005 | 0.00080 | 0.00004 | 0.6737 | 0.9686 | 0.6881 |  |
| SS-R | 0.00080 | 0.00005 | 0.00080 | 0.00005 | 0.00080 | 0.00004 | 0.00080 | 0.00004 | 0.5231 | 0.8166 | 0.7493 |  |
| SS-L | 0.00082 | 0.00003 | 0.00082 | 0.00003 | 0.00083 | 0.00004 | 0.00081 | 0.00003 | 0.8417 | 0.3784 | 0.2208 |  |
| EC-R | 0.00079 | 0.00007 | 0.00077 | 0.00005 | 0.00077 | 0.00004 | 0.00077 | 0.00004 | 0.6045 | 0.2437 | 0.8538 |  |
| EC-L | 0.00081 | 0.00006 | 0.00079 | 0.00003 | 0.00079 | 0.00004 | 0.00079 | 0.00004 | 0.2635 | 0.5754 | 0.4256 |  |
| CGC-R | 0.00070 | 0.00003 | 0.00069 | 0.00003 | 0.00069 | 0.00003 | 0.00070 | 0.00003 | 0.9295 | 0.4348 | 0.0710 |  |
| CGC-L | 0.00070 | 0.00003 | 0.00069 | 0.00003 | 0.00069 | 0.00003 | 0.00070 | 0.00003 | 0.5267 | 0.5351 | 0.2821 |  |
| CGH-R | 0.00062 | 0.00008 | 0.00064 | 0.00006 | 0.00061 | 0.00007 | 0.00065 | 0.00006 | 0.0534 | 0.7776 | 0.0555 |  |
| CGH-L | 0.00066 | 0.00006 | 0.00067 | 0.00006 | 0.00067 | 0.00006 | 0.00069 | 0.00004 | 0.0926 | 0.0765 | 0.0611 |  |
| FX/ST-R | 0.00077 | 0.00006 | 0.00074 | 0.00005 | 0.00075 | 0.00005 | 0.00076 | 0.00005 | 0.9126 | 0.2694 | 0.8688 |  |
| FX/ST-L | 0.00077 | 0.00003 | 0.00077 | 0.00004 | 0.00077 | 0.00004 | 0.00078 | 0.00004 | 0.1528 | 0.0552 | 0.4226 |  |
| SLF-R | 0.00075 | 0.00004 | 0.00075 | 0.00003 | 0.00075 | 0.00003 | 0.00075 | 0.00003 | 0.7326 | 0.4504 | 0.7870 |  |
| SLF-L | 0.00074 | 0.00004 | 0.00074 | 0.00003 | 0.00074 | 0.00003 | 0.00075 | 0.00003 | 0.3930 | 0.7137 | 0.6753 |  |
| SFO-R | 0.00074 | 0.00006 | 0.00071 | 0.00006 | 0.00071 | 0.00005 | 0.00070 | 0.00005 | 0.2607 | 0.0917 | 0.2347 |  |
| SFO-L | 0.00072 | 0.00007 | 0.00069 | 0.00005 | 0.00069 | 0.00004 | 0.00070 | 0.00005 | 0.7278 | 0.3455 | 0.6525 |  |
| IFO-R | 0.00077 | 0.00006 | **0.00078** | **0.00005** | 0.00077 | 0.00005 | **0.00075** | **0.00005** | 0.6294 | **0.0021** | 0.0532 |  |
| IFO-L | 0.00079 | 0.00008 | 0.00078 | 0.00006 | 0.00078 | 0.00005 | 0.00078 | 0.00005 | 0.8782 | 0.3584 | 0.9770 |  |
| UNC-R | 0.00090 | 0.00011 | 0.00089 | 0.00009 | 0.00090 | 0.00010 | 0.00088 | 0.00008 | 0.8124 | 0.1003 | 0.2658 |  |
| UNC-L | 0.00090 | 0.00014 | 0.00088 | 0.00007 | 0.00091 | 0.00011 | 0.00091 | 0.00011 | 0.2545 | 0.2608 | 0.7565 |  |

| MO | | | | | | | | | | | | |
| --- | --- | --- | --- | --- | --- | --- | --- | --- | --- | --- | --- | --- |
|  | **Bulbar-onset**  **(n=19)** | | **Upper- limb-onset**  **(n=35)** | | **Lower-limb-onset**  **(n=23)** | | **Normal Controls (NCs)**  **(n=47)** | | ***Bulbar vs NCs*** | ***Upper-limb vs NCs*** | ***Lower-limb vs NCs*** |  |
|  | **Mean** | **SD** | **Mean** | **SD** | **Mean** | **SD** | **Mean** | **SD** | ***p*** | ***p*** | ***p*** |  |
| GCC | 0.87613 | 0.06287 | 0.88979 | 0.04084 | 0.87246 | 0.05113 | 0.86969 | 0.07485 | 0.6890 | 0.2715 | 0.9634 |  |
| BCC | 0.86643 | 0.05281 | 0.89323 | 0.02392 | 0.89355 | 0.03101 | 0.89156 | 0.03512 | 0.0350 | 0.4153 | 0.3096 |  |
| SCC | 0.90849 | 0.01796 | 0.90121 | 0.02803 | 0.91044 | 0.02224 | 0.89652 | 0.03426 | 0.0912 | 0.1409 | 0.0083 |  |
| FX | 0.77227 | 0.13292 | 0.84847 | 0.07349 | 0.82122 | 0.09270 | 0.80712 | 0.11069 | 0.2574 | 0.3310 | 0.9134 |  |
| CST-R | 0.56321 | 0.09924 | **0.49133** | **0.07413** | 0.51188 | 0.06204 | **0.55506** | **0.08596** | 0.9616 | **0.0029** | 0.1169 |  |
| CST-L | 0.54069 | 0.10065 | **0.52439** | **0.08505** | 0.53148 | 0.08891 | **0.59685** | **0.09593** | 0.0184 | **0.0004** | 0.0322 |  |
| ML-R | 0.73218 | 0.07167 | 0.73887 | 0.06617 | 0.73328 | 0.07050 | 0.70279 | 0.05903 | 0.0817 | 0.0164 | 0.0546 |  |
| ML-L | 0.80242 | 0.05044 | 0.81656 | 0.05379 | **0.83155** | **0.04827** | **0.79031** | **0.06299** | 0.3303 | 0.0335 | **0.0013** |  |
| ICP-R | 0.54197 | 0.04942 | 0.54633 | 0.05108 | **0.52045** | **0.05289** | **0.56903** | **0.05575** | 0.1936 | 0.0104 | **0.0001** |  |
| ICP-L | 0.58104 | 0.06216 | 0.60168 | 0.04270 | 0.56896 | 0.05892 | 0.58793 | 0.04909 | 0.8189 | 0.7263 | 0.1146 |  |
| SCP-R | 0.81630 | 0.04107 | 0.79994 | 0.03918 | 0.80085 | 0.03918 | 0.80831 | 0.04042 | 0.6388 | 0.8192 | 0.5692 |  |
| SCP-L | 0.81836 | 0.03803 | 0.79681 | 0.04739 | 0.79275 | 0.05264 | 0.80573 | 0.03981 | 0.4397 | 0.8067 | 0.2959 |  |
| CP-R | 0.79961 | 0.03767 | 0.78900 | 0.03453 | 0.79301 | 0.03979 | 0.79431 | 0.03957 | 0.7677 | 0.6825 | 0.5715 |  |
| CP-L | 0.73965 | 0.04929 | 0.75544 | 0.04679 | 0.75854 | 0.03708 | 0.75381 | 0.04454 | 0.3082 | 0.9041 | 0.5492 |  |
| ALIC-R | 0.81595 | 0.05756 | **0.78655** | **0.06153** | 0.79173 | 0.07467 | **0.75704** | **0.07192** | 0.0053 | **0.0043** | 0.0069 |  |
| ALIC-L | 0.82329 | 0.05706 | 0.78317 | 0.06673 | 0.78394 | 0.07050 | 0.78970 | 0.07745 | 0.3087 | 0.8394 | 0.8241 |  |
| PLIC-R | 0.82445 | 0.05602 | 0.79992 | 0.05297 | 0.80778 | 0.04811 | 0.80038 | 0.04817 | 0.2608 | 0.1107 | 0.0825 |  |
| PLIC-L | 0.80402 | 0.05768 | 0.79129 | 0.05766 | 0.79737 | 0.06695 | 0.79484 | 0.06326 | 0.9490 | 0.8199 | 0.5388 |  |
| RLIC-R | 0.66575 | 0.06259 | 0.65190 | 0.07035 | 0.66951 | 0.09400 | 0.64866 | 0.09107 | 0.8309 | 0.3839 | 0.1110 |  |
| RLIC-L | 0.66013 | 0.06078 | 0.65664 | 0.06587 | 0.66085 | 0.07204 | 0.64912 | 0.06192 | 0.3818 | 0.4249 | 0.5667 |  |
| ACR-R | 0.75161 | 0.05346 | 0.76408 | 0.05366 | 0.74598 | 0.05416 | 0.72661 | 0.06471 | 0.0997 | 0.0071 | 0.2754 |  |
| ACR-L | 0.72112 | 0.05227 | 0.72259 | 0.05452 | 0.71648 | 0.04654 | 0.70941 | 0.05394 | 0.4909 | 0.3801 | 0.6858 |  |
| SCR-R | 0.66791 | 0.07440 | 0.64476 | 0.07996 | 0.64098 | 0.09527 | 0.61582 | 0.08591 | 0.0625 | 0.0099 | 0.0483 |  |
| SCR-L | 0.67283 | 0.07294 | 0.65730 | 0.07306 | 0.66332 | 0.07198 | 0.64521 | 0.07430 | 0.3840 | 0.1253 | 0.2267 |  |
| PCR-R | 0.64019 | 0.10906 | 0.59738 | 0.08860 | 0.62559 | 0.12023 | 0.59633 | 0.11321 | 0.2750 | 0.4317 | 0.1105 |  |
| PCR-L | 0.59389 | 0.08730 | 0.59684 | 0.06924 | 0.60125 | 0.07561 | 0.58169 | 0.07336 | 0.6668 | 0.5008 | 0.2014 |  |
| PTR-R | 0.78856 | 0.05153 | 0.79630 | 0.04947 | 0.81077 | 0.03908 | 0.82062 | 0.05656 | 0.0707 | 0.0131 | 0.1012 |  |
| PTR-L | 0.77391 | 0.06808 | 0.79259 | 0.03716 | 0.81196 | 0.05212 | 0.81051 | 0.05049 | 0.0298 | 0.1040 | 0.5862 |  |
| SS-R | 0.64575 | 0.06478 | 0.63948 | 0.07059 | 0.65599 | 0.05824 | 0.63885 | 0.08882 | 0.9111 | 0.7084 | 0.5170 |  |
| SS-L | 0.68409 | 0.07092 | 0.66197 | 0.07971 | 0.68086 | 0.05620 | 0.68285 | 0.06585 | 0.8224 | 0.9299 | 0.7210 |  |
| EC-R | 0.54927 | 0.05001 | 0.52248 | 0.07796 | 0.53404 | 0.05919 | 0.53113 | 0.06578 | 0.1847 | 0.1810 | 0.9833 |  |
| EC-L | 0.59655 | 0.03773 | 0.62095 | 0.05084 | 0.61653 | 0.05862 | 0.61780 | 0.05250 | 0.2554 | 0.9859 | 0.4728 |  |
| CGC-R | 0.61969 | 0.09978 | 0.66158 | 0.07544 | 0.66709 | 0.07848 | 0.62550 | 0.09169 | 0.8002 | 0.3163 | 0.1304 |  |
| CGC-L | 0.67338 | 0.10159 | 0.67626 | 0.09812 | 0.66755 | 0.07989 | 0.65533 | 0.08557 | 0.2322 | 0.7766 | 0.7292 |  |
| CGH-R | 0.72086 | 0.11388 | 0.70067 | 0.11176 | 0.72660 | 0.08698 | 0.70700 | 0.09676 | 0.8426 | 0.7622 | 0.5570 |  |
| CGH-L | 0.76385 | 0.06515 | 0.75562 | 0.06294 | 0.76271 | 0.07631 | 0.72096 | 0.08770 | 0.0357 | 0.0276 | 0.0230 |  |
| FX/ST-R | 0.60528 | 0.09230 | 0.60387 | 0.08944 | 0.58647 | 0.08781 | 0.62293 | 0.08249 | 0.8582 | 0.1253 | 0.0046 |  |
| FX/ST-L | 0.55792 | 0.08478 | 0.60289 | 0.07439 | 0.60554 | 0.08247 | 0.60577 | 0.10078 | 0.0900 | 0.2197 | 0.3619 |  |
| SLF-R | 0.58187 | 0.05751 | 0.58676 | 0.04995 | 0.61007 | 0.05380 | 0.58485 | 0.03882 | 0.7460 | 0.9528 | 0.0948 |  |
| SLF-L | 0.55464 | 0.06551 | 0.53706 | 0.05945 | 0.55894 | 0.06080 | 0.52997 | 0.05443 | 0.3884 | 0.1367 | 0.0155 |  |
| SFO-R | 0.72184 | 0.09898 | **0.75593** | **0.09102** | 0.72427 | 0.07951 | **0.69646** | **0.11675** | 0.5604 | **0.0005** | 0.1162 |  |
| SFO-L | 0.82583 | 0.08212 | 0.80581 | 0.09793 | 0.79702 | 0.07850 | 0.77982 | 0.11256 | 0.2824 | 0.1265 | 0.1655 |  |
| IFO-R | 0.64356 | 0.12882 | 0.69094 | 0.08805 | 0.65859 | 0.16658 | 0.69187 | 0.12569 | 0.3688 | 0.7929 | 0.0507 |  |
| IFO-L | 0.72539 | 0.13022 | 0.73693 | 0.11957 | 0.76739 | 0.12699 | 0.74375 | 0.11907 | 0.6928 | 0.3727 | 0.7587 |  |
| UNC-R | 0.84024 | 0.08198 | 0.86566 | 0.07106 | 0.84742 | 0.06844 | 0.84470 | 0.08795 | 0.6221 | 0.1174 | 0.6071 |  |
| UNC-L | 0.80666 | 0.12820 | 0.81426 | 0.13458 | 0.85694 | 0.08145 | 0.84339 | 0.09035 | 0.1648 | 0.1453 | 0.6191 |  |

**Supplementary Table 6. Subgroup analysis according to the cognitive profiles**

| FA | | | | | | | | |
| --- | --- | --- | --- | --- | --- | --- | --- | --- |
|  | **ALS without CI**  **(n=35)** | | **ALS with CI**  **(n=36)** | | **Normal Controls (NCs)**  **(n=47)** | | **ALS without CI *vs NCs*** | **ALS with CI *vs NCs*** |
|  | **Mean** | **SD** | **Mean** | **SD** | **Mean** | **SD** | ***p*** | ***p*** |
| GCC | 0.59483 | 0.03525 | 0.58406 | 0.04263 | 0.58878 | 0.05875 | 0.4660 | 0.3820 |
| BCC | **0.65273** | **0.03417** | **0.64557** | **0.03788** | **0.67074** | **0.04151** | **0.0001** | **0.0009** |
| SCC | 0.75306 | 0.01747 | 0.75572 | 0.02095 | 0.75561 | 0.01804 | 0.3096 | 0.7001 |
| FX | **0.43319** | **0.08098** | 0.40284 | 0.07595 | **0.45405** | **0.10842** | **0.0035** | 0.0122 |
| CST-R | **0.50362** | **0.03554** | **0.52107** | **0.03997** | **0.54660** | **0.02990** | **0.0000** | **0.0005** |
| CST-L | **0.52475** | **0.03975** | **0.52955** | **0.04183** | **0.56130** | **0.03874** | **0.0001** | **0.0000** |
| ML-R | 0.56684 | 0.02663 | 0.57750 | 0.02991 | 0.57630 | 0.02893 | 0.0714 | 0.9809 |
| ML-L | 0.57263 | 0.02864 | 0.58673 | 0.03153 | 0.58692 | 0.03123 | 0.0752 | 0.9722 |
| ICP-R | **0.49290** | **0.02469** | 0.49954 | 0.03114 | **0.50961** | **0.02513** | **0.0063** | 0.0486 |
| ICP-L | 0.50015 | 0.02283 | 0.50423 | 0.02749 | 0.51494 | 0.02589 | 0.0171 | 0.0451 |
| SCP-R | 0.64208 | 0.03294 | 0.65405 | 0.03887 | 0.66294 | 0.02355 | 0.0200 | 0.1588 |
| SCP-L | **0.63713** | **0.02870** | **0.64301** | **0.03228** | **0.66074** | **0.02611** | **0.0042** | **0.0061** |
| CP-R | **0.62538** | **0.03990** | **0.62999** | **0.03453** | **0.65745** | **0.02912** | **0.0000** | **0.0011** |
| CP-L | **0.56917** | **0.02739** | 0.57717 | 0.02875 | **0.59318** | **0.02125** | **0.0000** | 0.0116 |
| ALIC-R | 0.58041 | 0.03755 | 0.57766 | 0.03497 | 0.58414 | 0.02829 | 0.1885 | 0.1547 |
| ALIC-L | 0.58919 | 0.02036 | 0.58677 | 0.03522 | 0.59180 | 0.02210 | 0.3982 | 0.0299 |
| PLIC-R | 0.64984 | 0.03383 | **0.64177** | **0.03524** | **0.65790** | **0.02091** | 0.2508 | **0.0065** |
| PLIC-L | 0.64883 | 0.02550 | 0.65077 | 0.02925 | 0.65428 | 0.02668 | 0.6887 | 0.2294 |
| RLIC-R | 0.56831 | 0.03053 | 0.56745 | 0.04051 | 0.56750 | 0.03798 | 0.8935 | 0.6192 |
| RLIC-L | 0.55850 | 0.02691 | 0.55622 | 0.02732 | 0.55694 | 0.02482 | 0.8180 | 0.4593 |
| ACR-R | 0.46065 | 0.03509 | 0.45553 | 0.03599 | 0.45722 | 0.03470 | 0.5510 | 0.6236 |
| ACR-L | 0.46015 | 0.02805 | 0.45536 | 0.03217 | 0.45751 | 0.02658 | 0.7972 | 0.3419 |
| SCR-R | 0.49506 | 0.02740 | 0.49098 | 0.02797 | 0.50194 | 0.02668 | 0.0963 | 0.0219 |
| SCR-L | **0.49879** | **0.02903** | **0.49738** | **0.02813** | **0.51152** | **0.02479** | **0.0091** | **0.0042** |
| PCR-R | 0.51764 | 0.03545 | 0.51914 | 0.03292 | 0.51739 | 0.02802 | 0.6893 | 0.9012 |
| PCR-L | 0.45095 | 0.02984 | 0.44999 | 0.02833 | 0.45754 | 0.02887 | 0.1549 | 0.1001 |
| PTR-R | 0.62457 | 0.04223 | 0.62029 | 0.03748 | 0.62826 | 0.03830 | 0.3465 | 0.3341 |
| PTR-L | 0.60746 | 0.04510 | 0.60114 | 0.04205 | 0.60201 | 0.03372 | 0.8060 | 0.7505 |
| SS-R | 0.53129 | 0.03175 | 0.53358 | 0.02747 | 0.53823 | 0.03169 | 0.2272 | 0.4082 |
| SS-L | 0.53306 | 0.02914 | 0.53553 | 0.03884 | 0.53290 | 0.03131 | 0.8200 | 0.9166 |
| EC-R | 0.42612 | 0.02307 | 0.42335 | 0.02329 | 0.42763 | 0.02639 | 0.0984 | 0.2133 |
| EC-L | 0.45577 | 0.02638 | 0.45313 | 0.02505 | 0.45438 | 0.02196 | 0.8644 | 0.3942 |
| CGC-R | 0.57647 | 0.03870 | 0.56771 | 0.04287 | 0.57046 | 0.03584 | 0.7145 | 0.5812 |
| CGC-L | 0.62501 | 0.03625 | 0.61778 | 0.04173 | 0.61472 | 0.03137 | 0.5177 | 0.7928 |
| CGH-R | 0.55443 | 0.05764 | 0.56399 | 0.06379 | 0.57432 | 0.05156 | 0.0612 | 0.7061 |
| CGH-L | 0.54279 | 0.04769 | 0.55494 | 0.05294 | 0.52971 | 0.04491 | 0.3122 | 0.0247 |
| FX/ST-R | **0.55076** | **0.04159** | 0.54924 | 0.03432 | **0.56728** | **0.04621** | **0.0029** | 0.0309 |
| FX/ST-L | 0.55042 | 0.03002 | 0.55088 | 0.03391 | 0.55499 | 0.04019 | 0.1446 | 0.2277 |
| SLF-R | 0.47698 | 0.03068 | 0.46893 | 0.02975 | 0.46794 | 0.02112 | 0.2050 | 0.6826 |
| SLF-L | 0.45855 | 0.03073 | 0.45531 | 0.03402 | 0.45957 | 0.02270 | 0.9904 | 0.5617 |
| SFO-R | 0.50499 | 0.04725 | 0.50136 | 0.03926 | 0.50558 | 0.03297 | 0.2906 | 0.3319 |
| SFO-L | 0.47871 | 0.03736 | 0.48094 | 0.04942 | 0.47782 | 0.02978 | 0.4394 | 0.6025 |
| IFO-R | 0.47760 | 0.04385 | 0.48284 | 0.05041 | 0.48753 | 0.04263 | 0.2624 | 0.4753 |
| IFO-L | 0.46129 | 0.04085 | 0.44882 | 0.05426 | 0.45949 | 0.05094 | 0.6468 | 0.3895 |
| UNC-R | 0.58480 | 0.07104 | 0.56703 | 0.06083 | 0.58159 | 0.06690 | 0.6549 | 0.3684 |
| UNC-L | 0.58093 | 0.06920 | 0.58331 | 0.06438 | 0.59191 | 0.06005 | 0.1594 | 0.7731 |

| AD | | | | | | | | |
| --- | --- | --- | --- | --- | --- | --- | --- | --- |
|  | **ALS without CI**  **(n=35)** | | **ALS with CI**  **(n=36)** | | **Normal Controls (NCs)**  **(n=47)** | | **ALS without CI *vs NCs*** | **ALS with CI *vs NCs*** |
|  | **Mean** | **SD** | **Mean** | **SD** | **Mean** | **SD** | ***p*** | ***p*** |
| GCC | 0.00147 | 0.00007 | 0.00148 | 0.00006 | 0.00146 | 0.00006 | 0.1195 | 0.0671 |
| BCC | 0.00158 | 0.00005 | 0.00157 | 0.00004 | 0.00159 | 0.00004 | 0.0858 | 0.1620 |
| SCC | 0.00164 | 0.00006 | 0.00165 | 0.00004 | 0.00164 | 0.00007 | 0.9405 | 0.5094 |
| FX | **0.00239** | **0.00021** | 0.00243 | 0.00023 | **0.00232** | **0.00026** | **0.0037** | 0.0552 |
| CST-R | **0.00112** | **0.00007** | **0.00115** | **0.00005** | **0.00121** | **0.00006** | **0.0000** | **0.0000** |
| CST-L | **0.00116** | **0.00007** | **0.00117** | **0.00007** | **0.00124** | **0.00007** | **0.0000** | **0.0000** |
| ML-R | 0.00127 | 0.00004 | **0.00130** | **0.00005** | **0.00127** | **0.00005** | 0.2017 | **0.0049** |
| ML-L | 0.00129 | 0.00004 | **0.00132** | **0.00006** | **0.00129** | **0.00004** | 0.8503 | **0.0024** |
| ICP-R | 0.00120 | 0.00003 | 0.00120 | 0.00004 | 0.00119 | 0.00004 | 0.4083 | 0.7503 |
| ICP-L | 0.00122 | 0.00003 | **0.00123** | **0.00004** | **0.00120** | **0.00004** | 0.4363 | **0.0007** |
| SCP-R | 0.00156 | 0.00005 | 0.00160 | 0.00007 | 0.00158 | 0.00007 | 0.4004 | 0.1660 |
| SCP-L | 0.00155 | 0.00006 | 0.00158 | 0.00007 | 0.00157 | 0.00006 | 0.5650 | 0.1626 |
| CP-R | 0.00137 | 0.00007 | **0.00136** | **0.00009** | **0.00141** | **0.00005** | 0.0052 | **0.0043** |
| CP-L | **0.00134** | **0.00006** | **0.00133** | **0.00007** | **0.00138** | **0.00005** | **0.0003** | **0.0035** |
| ALIC-R | 0.00126 | 0.00004 | 0.00128 | 0.00005 | 0.00126 | 0.00005 | 0.2918 | 0.1883 |
| ALIC-L | 0.00126 | 0.00004 | 0.00127 | 0.00004 | 0.00126 | 0.00005 | 0.3695 | 0.8753 |
| PLIC-R | 0.00133 | 0.00005 | 0.00133 | 0.00005 | 0.00133 | 0.00004 | 0.2502 | 0.9583 |
| PLIC-L | 0.00136 | 0.00004 | 0.00135 | 0.00005 | 0.00136 | 0.00005 | 0.9654 | 0.1711 |
| RLIC-R | 0.00128 | 0.00008 | 0.00128 | 0.00008 | 0.00128 | 0.00006 | 0.5542 | 0.5928 |
| RLIC-L | 0.00133 | 0.00006 | 0.00132 | 0.00005 | 0.00133 | 0.00005 | 0.9135 | 0.9178 |
| ACR-R | 0.00120 | 0.00005 | 0.00121 | 0.00003 | 0.00120 | 0.00005 | 0.2899 | 0.8111 |
| ACR-L | 0.00120 | 0.00004 | 0.00120 | 0.00004 | 0.00118 | 0.00004 | 0.8758 | 0.8758 |
| SCR-R | 0.00119 | 0.00005 | 0.00119 | 0.00004 | 0.00117 | 0.00005 | 0.0110 | 0.1432 |
| SCR-L | 0.00123 | 0.00004 | 0.00122 | 0.00003 | 0.00123 | 0.00004 | 0.5895 | 0.9890 |
| PCR-R | 0.00127 | 0.00007 | 0.00128 | 0.00006 | 0.00127 | 0.00007 | 0.2935 | 0.5494 |
| PCR-L | 0.00123 | 0.00006 | 0.00122 | 0.00005 | 0.00123 | 0.00005 | 0.4847 | 0.3710 |
| PTR-R | 0.00141 | 0.00005 | 0.00141 | 0.00005 | 0.00142 | 0.00005 | 0.1874 | 0.1231 |
| PTR-L | 0.00143 | 0.00005 | 0.00142 | 0.00005 | 0.00142 | 0.00005 | 0.4699 | 0.7039 |
| SS-R | 0.00131 | 0.00007 | 0.00130 | 0.00006 | 0.00132 | 0.00006 | 0.8226 | 0.2377 |
| SS-L | 0.00135 | 0.00006 | 0.00135 | 0.00006 | 0.00134 | 0.00005 | 0.3129 | 0.4796 |
| EC-R | 0.00115 | 0.00007 | 0.00115 | 0.00005 | 0.00115 | 0.00004 | 0.5537 | 0.8937 |
| EC-L | 0.00122 | 0.00005 | 0.00121 | 0.00005 | 0.00121 | 0.00005 | 0.3164 | 0.9976 |
| CGC-R | 0.00121 | 0.00006 | 0.00118 | 0.00006 | 0.00120 | 0.00006 | 0.7539 | 0.1307 |
| CGC-L | 0.00127 | 0.00006 | 0.00125 | 0.00007 | 0.00126 | 0.00007 | 0.7579 | 0.4167 |
| CGH-R | 0.00106 | 0.00009 | **0.00104** | **0.00011** | **0.00110** | **0.00009** | 0.0747 | **0.0054** |
| CGH-L | 0.00112 | 0.00008 | 0.00111 | 0.00009 | 0.00113 | 0.00008 | 0.3292 | 0.1628 |
| FX/ST-R | **0.00124** | **0.00008** | 0.00125 | 0.00007 | **0.00129** | **0.00007** | **0.0037** | 0.0637 |
| FX/ST-L | **0.00128** | **0.00006** | 0.00129 | 0.00007 | **0.00132** | **0.00006** | **0.0014** | 0.0498 |
| SLF-R | 0.00118 | 0.00005 | 0.00117 | 0.00005 | 0.00116 | 0.00004 | 0.0245 | 0.2996 |
| SLF-L | 0.00114 | 0.00005 | 0.00113 | 0.00005 | 0.00114 | 0.00005 | 0.6445 | 0.7217 |
| SFO-R | 0.00116 | 0.00008 | 0.00116 | 0.00005 | 0.00114 | 0.00007 | 0.0335 | 0.3236 |
| SFO-L | 0.00110 | 0.00006 | 0.00112 | 0.00006 | 0.00111 | 0.00007 | 0.6350 | 0.5045 |
| IFO-R | 0.00122 | 0.00006 | 0.00122 | 0.00006 | 0.00119 | 0.00006 | 0.0555 | 0.0355 |
| IFO-L | 0.00121 | 0.00009 | 0.00121 | 0.00009 | 0.00121 | 0.00007 | 0.9361 | 0.7708 |
| UNC-R | 0.00154 | 0.00010 | 0.00155 | 0.00011 | 0.00153 | 0.00008 | 0.0653 | 0.3973 |
| UNC-L | 0.00155 | 0.00015 | 0.00155 | 0.00013 | 0.00159 | 0.00013 | 0.2686 | 0.1097 |

| RD | | | | | | | | |
| --- | --- | --- | --- | --- | --- | --- | --- | --- |
|  | **ALS without CI**  **(n=35)** | | **ALS with CI**  **(n=36)** | | **Normal Controls (NCs)**  **(n=47)** | | **ALS without CI *vs NCs*** | **ALS with CI *vs NCs*** |
|  | **Mean** | **SD** | **Mean** | **SD** | **Mean** | **SD** | ***p*** | ***p*** |
| GCC | 0.00050 | 0.00005 | 0.00051 | 0.00006 | 0.00050 | 0.00008 | 0.2121 | 0.1260 |
| BCC | **0.00045** | **0.00005** | **0.00046** | **0.00005** | **0.00043** | **0.00005** | **0.0005** | **0.0042** |
| SCC | 0.00035 | 0.00003 | 0.00034 | 0.00003 | 0.00035 | 0.00003 | 0.2882 | 0.7046 |
| FX | **0.00129** | **0.00031** | 0.00136 | 0.00029 | **0.00121** | **0.00038** | **0.0040** | 0.0719 |
| CST-R | 0.00051 | 0.00004 | 0.00051 | 0.00005 | 0.00050 | 0.00004 | 0.0598 | 0.1323 |
| CST-L | 0.00049 | 0.00004 | 0.00051 | 0.00006 | 0.00049 | 0.00004 | 0.1824 | 0.0162 |
| ML-R | 0.00047 | 0.00003 | 0.00048 | 0.00004 | 0.00047 | 0.00003 | 0.2781 | 0.1328 |
| ML-L | 0.00047 | 0.00003 | 0.00047 | 0.00004 | 0.00046 | 0.00003 | 0.1740 | 0.1112 |
| ICP-R | **0.00054** | **0.00003** | **0.00053** | **0.00003** | **0.00051** | **0.00002** | **0.0002** | **0.0011** |
| ICP-L | **0.00054** | **0.00003** | **0.00054** | **0.00003** | **0.00051** | **0.00002** | **0.0007** | **0.0000** |
| SCP-R | 0.00050 | 0.00005 | 0.00049 | 0.00005 | 0.00047 | 0.00004 | 0.0561 | 0.0368 |
| SCP-L | 0.00050 | 0.00004 | **0.00050** | **0.00004** | **0.00047** | **0.00004** | 0.0080 | **0.0008** |
| CP-R | **0.00045** | **0.00005** | 0.00044 | 0.00004 | **0.00042** | **0.00003** | **0.0021** | 0.0450 |
| CP-L | 0.00050 | 0.00004 | 0.00049 | 0.00003 | 0.00048 | 0.00003 | 0.0364 | 0.3047 |
| ALIC-R | 0.00046 | 0.00005 | 0.00046 | 0.00004 | 0.00045 | 0.00003 | 0.1096 | 0.1171 |
| ALIC-L | 0.00045 | 0.00003 | 0.00045 | 0.00004 | 0.00044 | 0.00003 | 0.1951 | 0.0238 |
| PLIC-R | 0.00040 | 0.00004 | **0.00041** | **0.00004** | **0.00039** | **0.00002** | 0.1007 | **0.0052** |
| PLIC-L | 0.00041 | 0.00003 | 0.00041 | 0.00003 | 0.00041 | 0.00003 | 0.6212 | 0.3567 |
| RLIC-R | 0.00049 | 0.00004 | 0.00049 | 0.00005 | 0.00049 | 0.00004 | 0.8349 | 0.4674 |
| RLIC-L | 0.00051 | 0.00003 | 0.00051 | 0.00003 | 0.00051 | 0.00003 | 0.8240 | 0.4662 |
| ACR-R | 0.00056 | 0.00005 | 0.00056 | 0.00004 | 0.00056 | 0.00005 | 0.3420 | 0.6875 |
| ACR-L | 0.00056 | 0.00004 | 0.00056 | 0.00004 | 0.00056 | 0.00004 | 0.2737 | 0.1023 |
| SCR-R | 0.00052 | 0.00004 | 0.00052 | 0.00003 | 0.00051 | 0.00003 | 0.0217 | 0.0275 |
| SCR-L | 0.00053 | 0.00003 | 0.00053 | 0.00004 | 0.00052 | 0.00003 | 0.0276 | 0.0380 |
| PCR-R | 0.00054 | 0.00005 | 0.00054 | 0.00004 | 0.00054 | 0.00004 | 0.3484 | 0.9630 |
| PCR-L | 0.00060 | 0.00006 | 0.00059 | 0.00003 | 0.00059 | 0.00004 | 0.1166 | 0.7539 |
| PTR-R | 0.00046 | 0.00005 | 0.00047 | 0.00005 | 0.00046 | 0.00005 | 0.3365 | 0.4375 |
| PTR-L | 0.00049 | 0.00006 | 0.00050 | 0.00005 | 0.00050 | 0.00004 | 0.7055 | 0.7474 |
| SS-R | 0.00055 | 0.00004 | 0.00054 | 0.00004 | 0.00054 | 0.00004 | 0.3072 | 0.9124 |
| SS-L | 0.00056 | 0.00004 | 0.00055 | 0.00004 | 0.00055 | 0.00004 | 0.4545 | 0.5699 |
| EC-R | 0.00059 | 0.00005 | 0.00059 | 0.00004 | 0.00059 | 0.00004 | 0.1367 | 0.3418 |
| EC-L | 0.00058 | 0.00004 | 0.00058 | 0.00004 | 0.00058 | 0.00003 | 0.2725 | 0.2378 |
| CGC-R | 0.00044 | 0.00003 | 0.00044 | 0.00004 | 0.00045 | 0.00003 | 0.6821 | 0.6417 |
| CGC-L | 0.00041 | 0.00003 | 0.00041 | 0.00004 | 0.00042 | 0.00003 | 0.4316 | 0.7870 |
| CGH-R | 0.00043 | 0.00006 | 0.00042 | 0.00007 | 0.00043 | 0.00005 | 0.6099 | 0.3477 |
| CGH-L | 0.00046 | 0.00005 | 0.00045 | 0.00006 | 0.00047 | 0.00004 | 0.1840 | 0.0355 |
| FX/ST-R | 0.00050 | 0.00005 | 0.00051 | 0.00004 | 0.00050 | 0.00005 | 0.0843 | 0.1942 |
| FX/ST-L | 0.00051 | 0.00004 | 0.00051 | 0.00004 | 0.00052 | 0.00005 | 0.5369 | 0.9006 |
| SLF-R | 0.00054 | 0.00004 | 0.00054 | 0.00004 | 0.00054 | 0.00003 | 0.9671 | 0.9506 |
| SLF-L | 0.00055 | 0.00004 | 0.00055 | 0.00004 | 0.00055 | 0.00003 | 0.5913 | 0.6374 |
| SFO-R | 0.00049 | 0.00007 | 0.00050 | 0.00005 | 0.00048 | 0.00004 | 0.0375 | 0.2913 |
| SFO-L | 0.00049 | 0.00004 | 0.00050 | 0.00006 | 0.00050 | 0.00004 | 0.4043 | 0.3526 |
| IFO-R | 0.00055 | 0.00005 | 0.00055 | 0.00006 | 0.00053 | 0.00005 | 0.0199 | 0.0442 |
| IFO-L | 0.00056 | 0.00005 | 0.00057 | 0.00006 | 0.00056 | 0.00006 | 0.5590 | 0.6022 |
| UNC-R | 0.00057 | 0.00012 | 0.00059 | 0.00010 | 0.00056 | 0.00010 | 0.1469 | 0.2585 |
| UNC-L | 0.00058 | 0.00012 | 0.00056 | 0.00010 | 0.00057 | 0.00011 | 0.2864 | 0.4329 |

| MD | | | | | | | | |
| --- | --- | --- | --- | --- | --- | --- | --- | --- |
|  | **ALS without CI**  **(n=35)** | | **ALS with CI**  **(n=36)** | | **Normal Controls (NCs)**  **(n=47)** | | **ALS without CI *vs NCs*** | **ALS with CI *vs NCs*** |
|  | **Mean** | **SD** | **Mean** | **SD** | **Mean** | **SD** | ***p*** | ***p*** |
| GCC | 0.00082 | 0.00005 | 0.00084 | 0.00005 | 0.00082 | 0.00006 | 0.0887 | 0.0404 |
| BCC | 0.00083 | 0.00004 | 0.00083 | 0.00003 | 0.00082 | 0.00004 | 0.0209 | 0.0567 |
| SCC | 0.00078 | 0.00003 | 0.00078 | 0.00002 | 0.00078 | 0.00004 | 0.5052 | 0.5253 |
| FX | 0.00166 | 0.00028 | 0.00172 | 0.00027 | 0.00158 | 0.00034 | 0.0035 | 0.0636 |
| CST-R | 0.00072 | 0.00004 | 0.00072 | 0.00004 | 0.00073 | 0.00004 | 0.1802 | 0.1676 |
| CST-L | 0.00072 | 0.00004 | 0.00073 | 0.00006 | 0.00074 | 0.00003 | 0.1336 | 0.5294 |
| ML-R | 0.00074 | 0.00002 | 0.00075 | 0.00003 | 0.00073 | 0.00003 | 0.8914 | 0.0076 |
| ML-L | 0.00075 | 0.00003 | 0.00075 | 0.00004 | 0.00074 | 0.00003 | 0.3653 | 0.0043 |
| ICP-R | 0.00076 | 0.00002 | 0.00075 | 0.00003 | 0.00074 | 0.00002 | 0.0085 | 0.0046 |
| ICP-L | 0.00076 | 0.00002 | **0.00077** | **0.00003** | **0.00074** | **0.00002** | 0.0023 | **0.0000** |
| SCP-R | 0.00085 | 0.00004 | 0.00086 | 0.00004 | 0.00084 | 0.00004 | 0.2895 | 0.0200 |
| SCP-L | 0.00085 | 0.00004 | **0.00086** | **0.00004** | **0.00083** | **0.00004** | 0.1024 | **0.0013** |
| CP-R | 0.00075 | 0.00005 | 0.00075 | 0.00005 | 0.00075 | 0.00003 | 0.6102 | 0.8513 |
| CP-L | 0.00078 | 0.00004 | 0.00077 | 0.00004 | 0.00078 | 0.00003 | 0.4647 | 0.3113 |
| ALIC-R | 0.00073 | 0.00005 | 0.00074 | 0.00004 | 0.00072 | 0.00003 | 0.0995 | 0.0826 |
| ALIC-L | 0.00072 | 0.00003 | 0.00072 | 0.00004 | 0.00072 | 0.00003 | 0.1540 | 0.0790 |
| PLIC-R | 0.00071 | 0.00003 | 0.00072 | 0.00004 | 0.00071 | 0.00002 | 0.0736 | 0.0371 |
| PLIC-L | 0.00073 | 0.00002 | 0.00072 | 0.00003 | 0.00073 | 0.00002 | 0.6597 | 0.8886 |
| RLIC-R | 0.00075 | 0.00005 | 0.00075 | 0.00006 | 0.00075 | 0.00004 | 0.6610 | 0.4754 |
| RLIC-L | 0.00079 | 0.00003 | 0.00078 | 0.00003 | 0.00078 | 0.00003 | 0.9354 | 0.6825 |
| ACR-R | 0.00077 | 0.00005 | 0.00078 | 0.00003 | 0.00078 | 0.00004 | 0.2399 | 0.6806 |
| ACR-L | 0.00077 | 0.00003 | 0.00078 | 0.00004 | 0.00077 | 0.00003 | 0.1003 | 0.0490 |
| SCR-R | 0.00074 | 0.00003 | 0.00075 | 0.00003 | 0.00073 | 0.00003 | 0.0046 | 0.0231 |
| SCR-L | 0.00076 | 0.00003 | 0.00076 | 0.00003 | 0.00075 | 0.00003 | 0.0619 | 0.1285 |
| PCR-R | 0.00078 | 0.00005 | 0.00078 | 0.00004 | 0.00078 | 0.00005 | 0.2534 | 0.8058 |
| PCR-L | 0.00081 | 0.00005 | 0.00080 | 0.00003 | 0.00080 | 0.00004 | 0.1520 | 0.8880 |
| PTR-R | 0.00078 | 0.00005 | 0.00078 | 0.00004 | 0.00078 | 0.00004 | 0.8531 | 0.9483 |
| PTR-L | 0.00080 | 0.00005 | 0.00081 | 0.00005 | 0.00080 | 0.00004 | 0.5914 | 0.9020 |
| SS-R | 0.00080 | 0.00005 | 0.00079 | 0.00004 | 0.00080 | 0.00004 | 0.5737 | 0.6220 |
| SS-L | 0.00082 | 0.00004 | 0.00082 | 0.00003 | 0.00081 | 0.00003 | 0.2929 | 0.4381 |
| EC-R | 0.00077 | 0.00005 | 0.00078 | 0.00004 | 0.00077 | 0.00004 | 0.2316 | 0.5125 |
| EC-L | 0.00079 | 0.00004 | 0.00079 | 0.00004 | 0.00079 | 0.00004 | 0.2335 | 0.4479 |
| CGC-R | 0.00070 | 0.00003 | 0.00069 | 0.00003 | 0.00070 | 0.00003 | 0.9353 | 0.1205 |
| CGC-L | 0.00070 | 0.00003 | 0.00069 | 0.00003 | 0.00070 | 0.00003 | 0.7467 | 0.3968 |
| CGH-R | 0.00064 | 0.00006 | 0.00062 | 0.00008 | 0.00065 | 0.00006 | 0.5916 | 0.0532 |
| CGH-L | 0.00068 | 0.00005 | 0.00067 | 0.00006 | 0.00069 | 0.00004 | 0.1878 | 0.0327 |
| FX/ST-R | 0.00075 | 0.00005 | 0.00075 | 0.00005 | 0.00076 | 0.00005 | 0.7002 | 0.9107 |
| FX/ST-L | 0.00077 | 0.00004 | 0.00077 | 0.00004 | 0.00078 | 0.00004 | 0.0253 | 0.2301 |
| SLF-R | 0.00075 | 0.00003 | 0.00075 | 0.00003 | 0.00075 | 0.00003 | 0.2942 | 0.6527 |
| SLF-L | 0.00075 | 0.00003 | 0.00074 | 0.00003 | 0.00075 | 0.00003 | 0.8823 | 0.5886 |
| SFO-R | 0.00072 | 0.00007 | 0.00072 | 0.00004 | 0.00070 | 0.00005 | 0.0183 | 0.2465 |
| SFO-L | 0.00069 | 0.00004 | 0.00071 | 0.00006 | 0.00070 | 0.00005 | 0.4306 | 0.3478 |
| IFO-R | 0.00078 | 0.00005 | 0.00077 | 0.00005 | 0.00075 | 0.00005 | 0.0092 | 0.0143 |
| IFO-L | 0.00078 | 0.00006 | 0.00078 | 0.00005 | 0.00078 | 0.00005 | 0.7147 | 0.8330 |
| UNC-R | 0.00089 | 0.00011 | 0.00091 | 0.00010 | 0.00088 | 0.00008 | 0.0750 | 0.2363 |
| UNC-L | 0.00090 | 0.00012 | 0.00089 | 0.00009 | 0.00091 | 0.00011 | 0.8071 | 0.2121 |

| MO | | | | | | | | |
| --- | --- | --- | --- | --- | --- | --- | --- | --- |
|  | **ALS without CI**  **(n=35)** | | **ALS with CI**  **(n=36)** | | **Normal Controls (NCs)**  **(n=47)** | | **ALS without CI *vs NCs*** | **ALS with CI *vs NCs*** |
|  | **Mean** | **SD** | **Mean** | **SD** | **Mean** | **SD** | ***p*** | ***p*** |
| GCC | 0.88220 | 0.04268 | 0.88043 | 0.05504 | 0.86969 | 0.07485 | 0.5658 | 0.4387 |
| BCC | 0.89039 | 0.02981 | 0.88832 | 0.03182 | 0.89156 | 0.03512 | 0.0746 | 0.9688 |
| SCC | 0.90270 | 0.02547 | 0.90900 | 0.02475 | 0.89652 | 0.03426 | 0.0890 | 0.0424 |
| FX | 0.82772 | 0.10391 | 0.81507 | 0.10505 | 0.80712 | 0.11069 | 0.7081 | 0.3732 |
| CST-R | 0.51522 | 0.07775 | 0.52120 | 0.08739 | 0.55506 | 0.08596 | 0.2764 | 0.0215 |
| CST-L | 0.54466 | 0.08748 | **0.53547** | **0.08509** | **0.59685** | **0.09593** | 0.0415 | **0.0007** |
| ML-R | 0.73632 | 0.06464 | 0.73857 | 0.07412 | 0.70279 | 0.05903 | 0.0265 | 0.0060 |
| ML-L | 0.82790 | 0.04160 | 0.80836 | 0.05942 | 0.79031 | 0.06299 | 0.0033 | 0.0494 |
| ICP-R | **0.52946** | **0.04474** | 0.54000 | 0.06288 | **0.56903** | **0.05575** | **0.0001** | 0.0148 |
| ICP-L | 0.58378 | 0.05378 | 0.58925 | 0.05758 | 0.58793 | 0.04909 | 0.3834 | 0.7670 |
| SCP-R | 0.79847 | 0.03497 | 0.80980 | 0.04337 | 0.80831 | 0.04042 | 0.7363 | 0.6352 |
| SCP-L | 0.79413 | 0.05086 | 0.80582 | 0.04543 | 0.80573 | 0.03981 | 0.6444 | 0.7701 |
| CP-R | 0.79669 | 0.03147 | 0.78088 | 0.03670 | 0.79431 | 0.03957 | 0.3915 | 0.1324 |
| CP-L | 0.75063 | 0.04954 | 0.75477 | 0.03487 | 0.75381 | 0.04454 | 0.6134 | 0.8653 |
| ALIC-R | 0.77578 | 0.06762 | 0.80610 | 0.06169 | 0.75704 | 0.07192 | 0.0177 | 0.0056 |
| ALIC-L | 0.77296 | 0.06901 | 0.80114 | 0.06097 | 0.78970 | 0.07745 | 0.9802 | 0.9128 |
| PLIC-R | 0.80394 | 0.04613 | 0.80352 | 0.05794 | 0.80038 | 0.04817 | 0.0451 | 0.4875 |
| PLIC-L | 0.78508 | 0.06198 | 0.79914 | 0.06028 | 0.79484 | 0.06326 | 0.8186 | 0.6066 |
| RLIC-R | 0.65047 | 0.08008 | 0.66749 | 0.07510 | 0.64866 | 0.09107 | 0.3577 | 0.3342 |
| RLIC-L | 0.66547 | 0.07160 | 0.64801 | 0.05502 | 0.64912 | 0.06192 | 0.2223 | 0.8812 |
| ACR-R | 0.75909 | 0.04828 | 0.75303 | 0.05880 | 0.72661 | 0.06471 | 0.0103 | 0.0738 |
| ACR-L | 0.71542 | 0.04923 | 0.72244 | 0.05157 | 0.70941 | 0.05394 | 0.5082 | 0.4521 |
| SCR-R | 0.63037 | 0.08462 | 0.66168 | 0.08088 | 0.61582 | 0.08591 | 0.0571 | 0.0245 |
| SCR-L | 0.64106 | 0.07534 | 0.67786 | 0.05858 | 0.64521 | 0.07430 | 0.6926 | 0.0914 |
| PCR-R | 0.59503 | 0.11106 | 0.63635 | 0.10204 | 0.59633 | 0.11321 | 0.4788 | 0.1615 |
| PCR-L | 0.59177 | 0.07051 | 0.60760 | 0.07672 | 0.58169 | 0.07336 | 0.3711 | 0.8387 |
| PTR-R | 0.80180 | 0.05122 | 0.79780 | 0.04330 | 0.82062 | 0.05656 | 0.0375 | 0.0513 |
| PTR-L | 0.80667 | 0.04648 | 0.78723 | 0.04631 | 0.81051 | 0.05049 | 0.5366 | 0.0454 |
| SS-R | 0.64192 | 0.07015 | 0.65528 | 0.05615 | 0.63885 | 0.08882 | 0.6521 | 0.5080 |
| SS-L | 0.67001 | 0.07045 | 0.67535 | 0.07569 | 0.68285 | 0.06585 | 0.9424 | 0.8480 |
| EC-R | 0.53746 | 0.05973 | 0.53016 | 0.07516 | 0.53113 | 0.06578 | 0.9580 | 0.8367 |
| EC-L | 0.61997 | 0.06180 | 0.60387 | 0.03932 | 0.61780 | 0.05250 | 0.7139 | 0.2050 |
| CGC-R | 0.67156 | 0.07580 | 0.64334 | 0.08335 | 0.62550 | 0.09169 | 0.0648 | 0.3406 |
| CGC-L | 0.69537 | 0.07620 | 0.66643 | 0.09201 | 0.65533 | 0.08557 | 0.1195 | 0.6223 |
| CGH-R | 0.73439 | 0.08483 | 0.70157 | 0.12115 | 0.70700 | 0.09676 | 0.1576 | 0.8565 |
| CGH-L | 0.76154 | 0.06803 | 0.76051 | 0.06669 | 0.72096 | 0.08770 | 0.0056 | 0.0086 |
| FX/ST-R | 0.58387 | 0.09725 | 0.61273 | 0.08457 | 0.62293 | 0.08249 | 0.0100 | 0.2474 |
| FX/ST-L | 0.59932 | 0.08045 | 0.59167 | 0.07512 | 0.60577 | 0.10078 | 0.2505 | 0.2133 |
| SLF-R | 0.59856 | 0.05709 | 0.58997 | 0.05134 | 0.58485 | 0.03882 | 0.4262 | 0.1660 |
| SLF-L | 0.55434 | 0.05416 | 0.54614 | 0.07003 | 0.52997 | 0.05443 | 0.0049 | 0.0448 |
| SFO-R | 0.72187 | 0.09204 | 0.74858 | 0.08801 | 0.69646 | 0.11675 | 0.0958 | 0.0740 |
| SFO-L | 0.79159 | 0.09995 | 0.81766 | 0.07725 | 0.77982 | 0.11256 | 0.2207 | 0.1916 |
| IFO-R | 0.68387 | 0.11333 | 0.65875 | 0.13223 | 0.69187 | 0.12569 | 0.3340 | 0.2106 |
| IFO-L | 0.73016 | 0.14447 | 0.74832 | 0.09594 | 0.74375 | 0.11907 | 0.2020 | 0.8996 |
| UNC-R | 0.84979 | 0.07041 | 0.85771 | 0.07891 | 0.84470 | 0.08795 | 0.4466 | 0.5186 |
| UNC-L | 0.81777 | 0.10884 | 0.83835 | 0.13195 | 0.84339 | 0.09035 | 0.1718 | 0.7065 |

**Supplementary Table 7. Subgroup analysis according to the cognitive profiles**

| FA | | | | | | | | | | | |
| --- | --- | --- | --- | --- | --- | --- | --- | --- | --- | --- | --- |
|  | **Group A**  **(n=27)** | | **Group B**  **(n=35)** | | **Group C**  **(n=16)** | | **Normal Controls (NCs)**  **(n=47)** | | ***Group A vs NCs*** | ***Group B vs NCs*** | ***Group C vs NCs*** |
|  | **Mean** | **SD** | **Mean** | **SD** | **Mean** | **SD** | **Mean** | **SD** | ***p*** | ***p*** | ***p*** |
| GCC | 0.57683 | 0.03669 | 0.58937 | 0.04703 | 0.59101 | 0.04295 | 0.58878 | 0.05875 | 0.1101 | 0.3115 | 0.5862 |
| BCC | **0.64317** | **0.04284** | **0.64934** | **0.04074** | **0.63668** | **0.03725** | **0.67074** | **0.04151** | **0.0002** | **0.0001** | **0.0002** |
| SCC | 0.75627 | 0.02167 | 0.75423 | 0.02141 | 0.74664 | 0.00870 | 0.75561 | 0.01804 | 0.7456 | 0.4357 | 0.0766 |
| FX | **0.41072** | **0.07389** | **0.41912** | **0.08444** | 0.41285 | 0.07518 | **0.45405** | **0.10842** | **0.0070** | **0.0023** | 0.0188 |
| CST-R | **0.52007** | **0.03900** | **0.51081** | **0.03835** | **0.49889** | **0.03870** | **0.54660** | **0.02990** | **0.0008** | **0.0000** | **0.0000** |
| CST-L | **0.53250** | **0.03848** | **0.52654** | **0.04495** | **0.50506** | **0.03383** | **0.56130** | **0.03874** | **0.0004** | **0.0000** | **0.0000** |
| ML-R | 0.57307 | 0.03443 | 0.57151 | 0.02772 | 0.56606 | 0.02015 | 0.57630 | 0.02893 | 0.3679 | 0.3367 | 0.2348 |
| ML-L | 0.58167 | 0.03818 | 0.58019 | 0.02805 | 0.57226 | 0.01855 | 0.58692 | 0.03123 | 0.3425 | 0.4655 | 0.2929 |
| ICP-R | 0.49789 | 0.02982 | 0.49645 | 0.02799 | **0.48807** | **0.02340** | **0.50961** | **0.02513** | 0.0394 | 0.0163 | **0.0064** |
| ICP-L | 0.50633 | 0.02370 | **0.49888** | **0.02795** | **0.49653** | **0.02469** | **0.51494** | **0.02589** | 0.1640 | **0.0051** | **0.0116** |
| SCP-R | 0.65618 | 0.03341 | 0.65068 | 0.03303 | **0.62537** | **0.04484** | **0.66294** | **0.02355** | 0.4408 | 0.1077 | **0.0001** |
| SCP-L | 0.65031 | 0.02602 | **0.64158** | **0.03054** | **0.61714** | **0.02968** | **0.66074** | **0.02611** | 0.1983 | **0.0024** | **0.0000** |
| CP-R | **0.63156** | **0.03552** | **0.62751** | **0.04189** | **0.61089** | **0.02981** | **0.65745** | **0.02912** | **0.0019** | **0.0001** | **0.0000** |
| CP-L | 0.57874 | 0.02601 | **0.57399** | **0.03152** | **0.55929** | **0.02413** | **0.59318** | **0.02125** | 0.0245 | **0.0003** | **0.0001** |
| ALIC-R | 0.57011 | 0.03934 | 0.58927 | 0.02848 | 0.57018 | 0.03418 | 0.58414 | 0.02829 | 0.0232 | 0.9818 | 0.1084 |
| ALIC-L | 0.58230 | 0.03103 | 0.58879 | 0.03259 | 0.58824 | 0.01826 | 0.59180 | 0.02210 | 0.0700 | 0.2087 | 0.2986 |
| PLIC-R | 0.64699 | 0.03611 | 0.65310 | 0.03144 | **0.62832** | **0.02871** | **0.65790** | **0.02091** | 0.0761 | 0.3667 | **0.0027** |
| PLIC-L | 0.64944 | 0.02804 | 0.65603 | 0.02879 | 0.63696 | 0.01973 | 0.65428 | 0.02668 | 0.4696 | 0.7063 | 0.0443 |
| RLIC-R | 0.55857 | 0.02905 | 0.58187 | 0.04085 | 0.56371 | 0.03166 | 0.56750 | 0.03798 | 0.3193 | 0.1068 | 0.9342 |
| RLIC-L | 0.55178 | 0.02373 | 0.56148 | 0.03094 | 0.54807 | 0.02007 | 0.55694 | 0.02482 | 0.2860 | 0.7686 | 0.1810 |
| ACR-R | 0.45195 | 0.03879 | 0.45921 | 0.03949 | 0.45541 | 0.02862 | 0.45722 | 0.03470 | 0.2654 | 0.5712 | 0.6075 |
| ACR-L | 0.44953 | 0.03423 | 0.45965 | 0.03684 | 0.45754 | 0.02371 | 0.45751 | 0.02658 | 0.1334 | 0.5744 | 0.8833 |
| SCR-R | 0.49402 | 0.03108 | 0.49457 | 0.02724 | 0.48629 | 0.02237 | 0.50194 | 0.02668 | 0.0746 | 0.0726 | 0.0672 |
| SCR-L | **0.49504** | **0.03025** | 0.50106 | 0.03117 | 0.49125 | 0.02019 | **0.51152** | **0.02479** | **0.0047** | 0.0176 | 0.0041 |
| PCR-R | 0.51433 | 0.03505 | 0.52227 | 0.03646 | 0.50944 | 0.02492 | 0.51739 | 0.02802 | 0.4072 | 0.7143 | 0.5011 |
| PCR-L | 0.44367 | 0.03162 | 0.45367 | 0.02978 | 0.44692 | 0.02383 | 0.45754 | 0.02887 | 0.0120 | 0.2414 | 0.2264 |
| PTR-R | 0.61959 | 0.03750 | 0.63245 | 0.04176 | 0.60850 | 0.03098 | 0.62826 | 0.03830 | 0.2279 | 0.8640 | 0.0616 |
| PTR-L | 0.60260 | 0.04063 | 0.61216 | 0.04749 | 0.58908 | 0.03245 | 0.60201 | 0.03372 | 0.7736 | 0.3685 | 0.2447 |
| SS-R | 0.52990 | 0.02323 | 0.53713 | 0.03275 | 0.52798 | 0.02997 | 0.53823 | 0.03169 | 0.1361 | 0.6879 | 0.4174 |
| SS-L | 0.53456 | 0.03233 | 0.53601 | 0.03539 | 0.52800 | 0.03413 | 0.53290 | 0.03131 | 0.9434 | 0.7727 | 0.7946 |
| EC-R | 0.42373 | 0.02484 | 0.42635 | 0.02660 | 0.41762 | 0.01755 | 0.42763 | 0.02639 | 0.1662 | 0.1868 | 0.0767 |
| EC-L | 0.44714 | 0.02855 | 0.46023 | 0.02743 | 0.44681 | 0.02015 | 0.45438 | 0.02196 | 0.0935 | 0.6150 | 0.1880 |
| CGC-R | 0.56236 | 0.04329 | 0.57652 | 0.04781 | 0.56268 | 0.02812 | 0.57046 | 0.03584 | 0.3572 | 0.8123 | 0.4787 |
| CGC-L | 0.60496 | 0.03718 | 0.62965 | 0.04495 | 0.61183 | 0.03606 | 0.61472 | 0.03137 | 0.1308 | 0.2397 | 0.4299 |
| CGH-R | 0.55096 | 0.06274 | 0.56853 | 0.05722 | 0.55030 | 0.06179 | 0.57432 | 0.05156 | 0.1179 | 0.4901 | 0.2026 |
| CGH-L | 0.55189 | 0.05493 | 0.55190 | 0.05063 | 0.53642 | 0.04226 | 0.52971 | 0.04491 | 0.0745 | 0.0702 | 0.6206 |
| FX/ST-R | 0.54492 | 0.02989 | 0.55546 | 0.04864 | 0.55025 | 0.03085 | 0.56728 | 0.04621 | 0.0134 | 0.0370 | 0.0831 |
| FX/ST-L | 0.55331 | 0.02639 | 0.54839 | 0.03656 | 0.54100 | 0.03441 | 0.55499 | 0.04019 | 0.4615 | 0.0578 | 0.0386 |
| SLF-R | 0.47322 | 0.02493 | 0.47904 | 0.03249 | 0.45469 | 0.02505 | 0.46794 | 0.02112 | 0.4350 | 0.0930 | 0.1928 |
| SLF-L | 0.45650 | 0.02987 | 0.46358 | 0.03419 | 0.43555 | 0.01852 | 0.45957 | 0.02270 | 0.6430 | 0.6827 | 0.0206 |
| SFO-R | 0.49494 | 0.04617 | 0.51254 | 0.03809 | 0.49354 | 0.04046 | 0.50558 | 0.03297 | 0.0629 | 0.8960 | 0.2925 |
| SFO-L | 0.46793 | 0.05679 | 0.47950 | 0.03824 | 0.48369 | 0.03261 | 0.47782 | 0.02978 | 0.0925 | 0.5828 | 0.9931 |
| IFO-R | 0.48057 | 0.04774 | 0.48697 | 0.04249 | 0.45436 | 0.05226 | 0.48753 | 0.04263 | 0.4442 | 0.6047 | 0.0372 |
| IFO-L | 0.45198 | 0.05231 | 0.46343 | 0.04955 | 0.44473 | 0.05059 | 0.45949 | 0.05094 | 0.3984 | 0.9611 | 0.5411 |
| UNC-R | 0.56951 | 0.06805 | 0.57762 | 0.06577 | 0.57922 | 0.05958 | 0.58159 | 0.06690 | 0.2621 | 0.5861 | 0.7627 |
| UNC-L | 0.57635 | 0.07429 | 0.58277 | 0.05825 | 0.58631 | 0.06440 | 0.59191 | 0.06005 | 0.2772 | 0.3117 | 0.7626 |

| AD | | | | | | | | | | | |
| --- | --- | --- | --- | --- | --- | --- | --- | --- | --- | --- | --- |
|  | **Group A**  **(n=27)** | | **Group B**  **(n=35)** | | **Group C**  **(n=16)** | | **Normal Controls (NCs)**  **(n=47)** | | ***Group A vs NCs*** | ***Group B vs NCs*** | ***Group C vs NCs*** |
|  | **Mean** | **SD** | **Mean** | **SD** | **Mean** | **SD** | **Mean** | **SD** | ***p*** | ***p*** | ***p*** |
| GCC | 0.00146 | 0.00006 | 0.00148 | 0.00007 | 0.00149 | 0.00007 | 0.00146 | 0.00006 | 0.5017 | 0.0127 | 0.1344 |
| BCC | 0.00156 | 0.00004 | 0.00158 | 0.00004 | 0.00159 | 0.00005 | 0.00159 | 0.00004 | 0.0103 | 0.2656 | 0.3817 |
| SCC | 0.00164 | 0.00004 | 0.00165 | 0.00005 | 0.00165 | 0.00006 | 0.00164 | 0.00007 | 0.8834 | 0.5104 | 0.7221 |
| FX | 0.00241 | 0.00016 | **0.00242** | **0.00022** | 0.00239 | 0.00026 | **0.00232** | **0.00026** | 0.0418 | **0.0030** | 0.1527 |
| CST-R | **0.00113** | **0.00007** | **0.00113** | **0.00006** | **0.00113** | **0.00004** | **0.00121** | **0.00006** | **0.0000** | **0.0000** | **0.0000** |
| CST-L | **0.00116** | **0.00008** | **0.00117** | **0.00007** | **0.00114** | **0.00006** | **0.00124** | **0.00007** | **0.0000** | **0.0000** | **0.0000** |
| ML-R | 0.00128 | 0.00006 | 0.00129 | 0.00004 | 0.00128 | 0.00005 | 0.00127 | 0.00005 | 0.4996 | 0.3270 | 0.9808 |
| ML-L | 0.00131 | 0.00006 | 0.00131 | 0.00005 | 0.00130 | 0.00005 | 0.00129 | 0.00004 | 0.1035 | 0.2347 | 0.4292 |
| ICP-R | 0.00118 | 0.00003 | 0.00120 | 0.00004 | 0.00121 | 0.00003 | 0.00119 | 0.00004 | 0.1091 | 0.6889 | 0.2155 |
| ICP-L | 0.00121 | 0.00003 | 0.00122 | 0.00004 | 0.00123 | 0.00004 | 0.00120 | 0.00004 | 0.4475 | 0.0671 | 0.0450 |
| SCP-R | 0.00160 | 0.00007 | 0.00158 | 0.00006 | 0.00156 | 0.00006 | 0.00158 | 0.00007 | 0.1024 | 0.8723 | 0.3513 |
| SCP-L | 0.00157 | 0.00007 | 0.00157 | 0.00007 | 0.00154 | 0.00005 | 0.00157 | 0.00006 | 0.2966 | 0.5684 | 0.2484 |
| CP-R | 0.00138 | 0.00010 | **0.00136** | **0.00007** | 0.00135 | 0.00006 | **0.00141** | **0.00005** | 0.1224 | **0.0016** | 0.0050 |
| CP-L | **0.00134** | **0.00006** | **0.00134** | **0.00006** | 0.00132 | 0.00006 | **0.00138** | **0.00005** | **0.0031** | **0.0015** | 0.0083 |
| ALIC-R | 0.00128 | 0.00006 | 0.00127 | 0.00005 | 0.00127 | 0.00005 | 0.00126 | 0.00005 | 0.0821 | 0.2412 | 0.8978 |
| ALIC-L | 0.00127 | 0.00005 | 0.00127 | 0.00005 | 0.00127 | 0.00004 | 0.00126 | 0.00005 | 0.4114 | 0.2961 | 0.6802 |
| PLIC-R | 0.00135 | 0.00006 | 0.00133 | 0.00005 | 0.00132 | 0.00002 | 0.00133 | 0.00004 | 0.0927 | 0.7419 | 0.2485 |
| PLIC-L | 0.00135 | 0.00004 | 0.00136 | 0.00004 | 0.00134 | 0.00004 | 0.00136 | 0.00005 | 0.1186 | 0.5870 | 0.1997 |
| RLIC-R | 0.00129 | 0.00007 | 0.00127 | 0.00010 | 0.00128 | 0.00004 | 0.00128 | 0.00006 | 0.3366 | 0.7965 | 0.8064 |
| RLIC-L | 0.00130 | 0.00005 | 0.00134 | 0.00005 | 0.00133 | 0.00006 | 0.00133 | 0.00005 | 0.0766 | 0.3674 | 0.5103 |
| ACR-R | 0.00120 | 0.00005 | 0.00120 | 0.00005 | 0.00122 | 0.00003 | 0.00120 | 0.00005 | 0.8744 | 0.5565 | 0.2855 |
| ACR-L | 0.00119 | 0.00004 | 0.00120 | 0.00004 | **0.00122** | **0.00003** | **0.00118** | **0.00004** | 0.6596 | 0.0160 | **0.0007** |
| SCR-R | 0.00119 | 0.00005 | 0.00119 | 0.00005 | 0.00120 | 0.00004 | 0.00117 | 0.00005 | 0.0980 | 0.0227 | 0.1159 |
| SCR-L | 0.00121 | 0.00004 | 0.00123 | 0.00004 | 0.00125 | 0.00004 | 0.00123 | 0.00004 | 0.3579 | 0.6055 | 0.0760 |
| PCR-R | 0.00127 | 0.00007 | 0.00127 | 0.00007 | 0.00128 | 0.00006 | 0.00127 | 0.00007 | 0.6209 | 0.3788 | 0.4646 |
| PCR-L | 0.00121 | 0.00006 | 0.00122 | 0.00004 | 0.00124 | 0.00004 | 0.00123 | 0.00005 | 0.4117 | 0.9012 | 0.3189 |
| PTR-R | 0.00141 | 0.00005 | 0.00140 | 0.00005 | 0.00139 | 0.00005 | 0.00142 | 0.00005 | 0.5388 | 0.0497 | 0.0138 |
| PTR-L | 0.00143 | 0.00006 | 0.00142 | 0.00004 | 0.00142 | 0.00004 | 0.00142 | 0.00005 | 0.7642 | 0.6802 | 0.5662 |
| SS-R | 0.00132 | 0.00007 | 0.00131 | 0.00006 | 0.00129 | 0.00007 | 0.00132 | 0.00006 | 0.9643 | 0.4316 | 0.1838 |
| SS-L | 0.00135 | 0.00006 | 0.00136 | 0.00006 | 0.00136 | 0.00005 | 0.00134 | 0.00005 | 0.9754 | 0.2467 | 0.2066 |
| EC-R | 0.00117 | 0.00006 | 0.00115 | 0.00006 | 0.00114 | 0.00006 | 0.00115 | 0.00004 | 0.2272 | 0.9967 | 0.2840 |
| EC-L | 0.00120 | 0.00006 | 0.00123 | 0.00005 | 0.00123 | 0.00005 | 0.00121 | 0.00005 | 0.2735 | 0.1513 | 0.1247 |
| CGC-R | 0.00119 | 0.00005 | 0.00120 | 0.00008 | 0.00118 | 0.00004 | 0.00120 | 0.00006 | 0.2888 | 0.7946 | 0.1980 |
| CGC-L | 0.00123 | 0.00006 | 0.00126 | 0.00007 | 0.00125 | 0.00008 | 0.00126 | 0.00007 | 0.1415 | 0.9289 | 0.4639 |
| CGH-R | 0.00104 | 0.00009 | 0.00105 | 0.00010 | 0.00104 | 0.00010 | 0.00110 | 0.00009 | 0.0138 | 0.0358 | 0.0338 |
| CGH-L | 0.00109 | 0.00008 | 0.00111 | 0.00009 | 0.00114 | 0.00008 | 0.00113 | 0.00008 | 0.0477 | 0.1261 | 0.9553 |
| FX/ST-R | 0.00126 | 0.00007 | **0.00124** | **0.00008** | 0.00123 | 0.00010 | **0.00129** | **0.00007** | 0.2241 | **0.0033** | 0.0218 |
| FX/ST-L | **0.00128** | **0.00007** | **0.00128** | **0.00006** | 0.00131 | 0.00008 | **0.00132** | **0.00006** | **0.0037** | **0.0009** | 0.7725 |
| SLF-R | 0.00116 | 0.00005 | 0.00117 | 0.00005 | 0.00118 | 0.00005 | 0.00116 | 0.00004 | 0.4663 | 0.0665 | 0.3357 |
| SLF-L | 0.00113 | 0.00005 | 0.00114 | 0.00005 | 0.00113 | 0.00004 | 0.00114 | 0.00005 | 0.5957 | 0.7210 | 0.4263 |
| SFO-R | 0.00116 | 0.00006 | 0.00116 | 0.00006 | 0.00115 | 0.00009 | 0.00114 | 0.00007 | 0.0984 | 0.0881 | 0.7465 |
| SFO-L | 0.00111 | 0.00006 | 0.00111 | 0.00007 | 0.00110 | 0.00006 | 0.00111 | 0.00007 | 0.6448 | 0.3696 | 0.6907 |
| IFO-R | 0.00121 | 0.00006 | 0.00121 | 0.00007 | 0.00122 | 0.00007 | 0.00119 | 0.00006 | 0.1280 | 0.1026 | 0.3268 |
| IFO-L | 0.00118 | 0.00009 | 0.00122 | 0.00008 | 0.00124 | 0.00010 | 0.00121 | 0.00007 | 0.0628 | 0.2877 | 0.1994 |
| UNC-R | 0.00155 | 0.00012 | 0.00154 | 0.00010 | 0.00152 | 0.00009 | 0.00153 | 0.00008 | 0.0789 | 0.2035 | 0.5417 |
| UNC-L | 0.00158 | 0.00016 | 0.00154 | 0.00013 | 0.00150 | 0.00012 | 0.00159 | 0.00013 | 0.7738 | 0.1033 | 0.0169 |

| RD | | | | | | | | | | | | |
| --- | --- | --- | --- | --- | --- | --- | --- | --- | --- | --- | --- | --- |
|  | **Group A**  **(n=27)** | | **Group B**  **(n=35)** | | **Group C**  **(n=16)** | | **Normal Controls (NCs)**  **(n=47)** | | ***Group A vs NCs*** | ***Group B vs NCs*** | ***Group C vs NCs*** |  |
|  | **Mean** | **SD** | **Mean** | **SD** | **Mean** | **SD** | **Mean** | **SD** | ***p*** | ***p*** | ***p*** |  |
| GCC | 0.00052 | 0.00006 | 0.00051 | 0.00007 | 0.00051 | 0.00006 | 0.00050 | 0.00008 | 0.0953 | 0.0580 | 0.2934 |  |
| BCC | 0.00046 | 0.00006 | **0.00046** | **0.00006** | **0.00048** | **0.00005** | **0.00043** | **0.00005** | 0.0032 | **0.0005** | **0.0004** |  |
| SCC | 0.00034 | 0.00003 | 0.00035 | 0.00003 | 0.00036 | 0.00002 | 0.00035 | 0.00003 | 0.8942 | 0.3314 | 0.1230 |  |
| FX | 0.00133 | 0.00024 | **0.00134** | **0.00032** | 0.00132 | 0.00032 | **0.00121** | **0.00038** | 0.0483 | **0.0039** | 0.0925 |  |
| CST-R | 0.00051 | 0.00004 | 0.00051 | 0.00004 | 0.00053 | 0.00004 | 0.00050 | 0.00004 | 0.3298 | 0.0736 | 0.0322 |  |
| CST-L | 0.00049 | 0.00006 | 0.00050 | 0.00005 | 0.00052 | 0.00003 | 0.00049 | 0.00004 | 0.2219 | 0.0244 | 0.0208 |  |
| ML-R | 0.00047 | 0.00003 | 0.00048 | 0.00003 | 0.00048 | 0.00003 | 0.00047 | 0.00003 | 0.2186 | 0.1409 | 0.1440 |  |
| ML-L | 0.00047 | 0.00004 | 0.00047 | 0.00003 | 0.00048 | 0.00003 | 0.00046 | 0.00003 | 0.0885 | 0.1956 | 0.1603 |  |
| ICP-R | 0.00053 | 0.00003 | **0.00053** | **0.00003** | **0.00055** | **0.00002** | **0.00051** | **0.00002** | 0.0059 | **0.0006** | **0.0000** |  |
| ICP-L | 0.00053 | 0.00003 | **0.00054** | **0.00003** | **0.00055** | **0.00003** | **0.00051** | **0.00002** | 0.0170 | **0.0000** | **0.0000** |  |
| SCP-R | 0.00049 | 0.00005 | 0.00049 | 0.00004 | **0.00052** | **0.00005** | **0.00047** | **0.00004** | 0.1533 | 0.1168 | **0.0003** |  |
| SCP-L | 0.00048 | 0.00003 | **0.00050** | **0.00004** | **0.00052** | **0.00004** | **0.00047** | **0.00004** | 0.1167 | **0.0007** | **0.0000** |  |
| CP-R | 0.00045 | 0.00005 | 0.00044 | 0.00005 | **0.00046** | **0.00004** | **0.00042** | **0.00003** | 0.0151 | 0.0092 | **0.0049** |  |
| CP-L | 0.00049 | 0.00003 | 0.00050 | 0.00004 | 0.00051 | 0.00003 | 0.00048 | 0.00003 | 0.4153 | 0.0557 | 0.0164 |  |
| ALIC-R | 0.00047 | 0.00005 | 0.00045 | 0.00004 | 0.00047 | 0.00005 | 0.00045 | 0.00003 | 0.0067 | 0.8653 | 0.1544 |  |
| ALIC-L | 0.00046 | 0.00004 | 0.00045 | 0.00004 | 0.00045 | 0.00002 | 0.00044 | 0.00003 | 0.0602 | 0.0929 | 0.1887 |  |
| PLIC-R | 0.00041 | 0.00004 | 0.00040 | 0.00004 | **0.00042** | **0.00003** | **0.00039** | **0.00002** | 0.0142 | 0.2452 | **0.0071** |  |
| PLIC-L | 0.00041 | 0.00003 | 0.00041 | 0.00003 | 0.00042 | 0.00003 | 0.00041 | 0.00003 | 0.8722 | 0.9887 | 0.0947 |  |
| RLIC-R | 0.00050 | 0.00004 | 0.00047 | 0.00005 | 0.00050 | 0.00004 | 0.00049 | 0.00004 | 0.1667 | 0.1476 | 0.8153 |  |
| RLIC-L | 0.00051 | 0.00003 | 0.00051 | 0.00004 | 0.00053 | 0.00003 | 0.00051 | 0.00003 | 0.9963 | 0.8590 | 0.0873 |  |
| ACR-R | 0.00057 | 0.00006 | 0.00056 | 0.00006 | 0.00057 | 0.00004 | 0.00056 | 0.00005 | 0.3527 | 0.4896 | 0.3655 |  |
| ACR-L | 0.00056 | 0.00004 | 0.00056 | 0.00006 | 0.00057 | 0.00003 | 0.00056 | 0.00004 | 0.2224 | 0.1249 | 0.1461 |  |
| SCR-R | 0.00052 | 0.00004 | 0.00052 | 0.00003 | 0.00053 | 0.00003 | 0.00051 | 0.00003 | 0.0517 | 0.0316 | 0.0276 |  |
| SCR-L | 0.00053 | 0.00004 | 0.00053 | 0.00004 | **0.00055** | **0.00003** | **0.00052** | **0.00003** | 0.0928 | 0.0568 | **0.0029** |  |
| PCR-R | 0.00054 | 0.00005 | 0.00053 | 0.00005 | 0.00055 | 0.00004 | 0.00054 | 0.00004 | 0.4548 | 0.9648 | 0.3185 |  |
| PCR-L | 0.00060 | 0.00006 | 0.00059 | 0.00004 | 0.00060 | 0.00003 | 0.00059 | 0.00004 | 0.1132 | 0.7010 | 0.2319 |  |
| PTR-R | 0.00047 | 0.00005 | 0.00045 | 0.00005 | 0.00048 | 0.00004 | 0.00046 | 0.00005 | 0.2022 | 0.8152 | 0.2031 |  |
| PTR-L | 0.00050 | 0.00006 | 0.00049 | 0.00006 | 0.00051 | 0.00004 | 0.00050 | 0.00004 | 0.6220 | 0.6946 | 0.3791 |  |
| SS-R | 0.00055 | 0.00004 | 0.00054 | 0.00005 | 0.00054 | 0.00004 | 0.00054 | 0.00004 | 0.1987 | 0.9090 | 0.9562 |  |
| SS-L | 0.00055 | 0.00004 | 0.00056 | 0.00004 | 0.00056 | 0.00004 | 0.00055 | 0.00004 | 0.7680 | 0.3718 | 0.2830 |  |
| EC-R | 0.00060 | 0.00005 | 0.00059 | 0.00005 | 0.00059 | 0.00005 | 0.00059 | 0.00004 | 0.0730 | 0.3256 | 0.8156 |  |
| EC-L | 0.00058 | 0.00005 | 0.00058 | 0.00005 | 0.00059 | 0.00003 | 0.00058 | 0.00003 | 0.3004 | 0.3436 | 0.0370 |  |
| CGC-R | 0.00045 | 0.00004 | 0.00044 | 0.00004 | 0.00045 | 0.00002 | 0.00045 | 0.00003 | 0.6541 | 0.5371 | 0.9446 |  |
| CGC-L | 0.00042 | 0.00004 | 0.00040 | 0.00004 | 0.00042 | 0.00002 | 0.00042 | 0.00003 | 0.3916 | 0.1390 | 0.8058 |  |
| CGH-R | 0.00043 | 0.00006 | 0.00042 | 0.00006 | 0.00043 | 0.00007 | 0.00043 | 0.00005 | 0.8916 | 0.6661 | 0.8060 |  |
| CGH-L | 0.00045 | 0.00006 | 0.00045 | 0.00006 | 0.00047 | 0.00005 | 0.00047 | 0.00004 | 0.0326 | 0.0560 | 0.7960 |  |
| FX/ST-R | 0.00051 | 0.00005 | 0.00050 | 0.00006 | 0.00050 | 0.00004 | 0.00050 | 0.00005 | 0.0497 | 0.4154 | 0.5305 |  |
| FX/ST-L | 0.00051 | 0.00004 | 0.00051 | 0.00004 | 0.00053 | 0.00005 | 0.00052 | 0.00005 | 0.2899 | 0.9103 | 0.0990 |  |
| SLF-R | 0.00054 | 0.00003 | 0.00053 | 0.00004 | 0.00056 | 0.00003 | 0.00054 | 0.00003 | 0.5921 | 0.4572 | 0.0600 |  |
| SLF-L | 0.00055 | 0.00004 | 0.00054 | 0.00003 | 0.00057 | 0.00003 | 0.00055 | 0.00003 | 0.5987 | 0.3197 | 0.2684 |  |
| SFO-R | 0.00051 | 0.00006 | 0.00048 | 0.00005 | 0.00051 | 0.00008 | 0.00048 | 0.00004 | 0.0192 | 0.5771 | 0.2266 |  |
| SFO-L | 0.00051 | 0.00007 | 0.00050 | 0.00006 | 0.00048 | 0.00003 | 0.00050 | 0.00004 | 0.0929 | 0.3555 | 0.7820 |  |
| IFO-R | 0.00055 | 0.00006 | 0.00054 | 0.00006 | **0.00058** | **0.00006** | **0.00053** | **0.00005** | 0.0814 | 0.0870 | **0.0052** |  |
| IFO-L | 0.00056 | 0.00006 | 0.00057 | 0.00006 | 0.00059 | 0.00007 | 0.00056 | 0.00006 | 0.6552 | 0.4628 | 0.1530 |  |
| UNC-R | 0.00059 | 0.00013 | 0.00057 | 0.00011 | 0.00056 | 0.00008 | 0.00056 | 0.00010 | 0.0690 | 0.2444 | 0.8048 |  |
| UNC-L | 0.00058 | 0.00013 | 0.00057 | 0.00010 | 0.00055 | 0.00008 | 0.00057 | 0.00011 | 0.5282 | 0.7949 | 0.5513 |  |

| MD | | | | | | | | | | | | |
| --- | --- | --- | --- | --- | --- | --- | --- | --- | --- | --- | --- | --- |
|  | **Group A**  **(n=27)** | | **Group B**  **(n=35)** | | **Group C**  **(n=16)** | | **Normal Controls (NCs)**  **(n=47)** | | ***Group A vs NCs*** | ***Group B vs NCs*** | ***Group C vs NCs*** |  |
|  | **Mean** | **SD** | **Mean** | **SD** | **Mean** | **SD** | **Mean** | **SD** | ***p*** | ***p*** | ***p*** |  |
| GCC | 0.00083 | 0.00005 | 0.00083 | 0.00007 | 0.00084 | 0.00006 | 0.00082 | 0.00006 | 0.1037 | 0.0102 | 0.1398 |  |
| BCC | 0.00083 | 0.00004 | 0.00083 | 0.00005 | 0.00085 | 0.00004 | 0.00082 | 0.00004 | 0.1106 | 0.0084 | 0.0055 |  |
| SCC | 0.00077 | 0.00003 | 0.00078 | 0.00003 | 0.00079 | 0.00003 | 0.00078 | 0.00004 | 0.8725 | 0.3264 | 0.4726 |  |
| FX | 0.00169 | 0.00021 | 0.00170 | 0.00028 | 0.00168 | 0.00030 | 0.00158 | 0.00034 | 0.0436 | 0.0033 | 0.1014 |  |
| CST-R | 0.00071 | 0.00004 | 0.00072 | 0.00004 | 0.00073 | 0.00004 | 0.00073 | 0.00004 | 0.0453 | 0.1648 | 0.4968 |  |
| CST-L | 0.00072 | 0.00006 | 0.00073 | 0.00005 | 0.00073 | 0.00004 | 0.00074 | 0.00003 | 0.1065 | 0.5033 | 0.2440 |  |
| ML-R | 0.00074 | 0.00003 | 0.00075 | 0.00002 | 0.00075 | 0.00003 | 0.00073 | 0.00003 | 0.1980 | 0.1015 | 0.2765 |  |
| ML-L | 0.00075 | 0.00004 | 0.00075 | 0.00003 | 0.00076 | 0.00003 | 0.00074 | 0.00003 | 0.0291 | 0.1025 | 0.1387 |  |
| ICP-R | 0.00074 | 0.00003 | 0.00075 | 0.00002 | **0.00077** | **0.00002** | **0.00074** | **0.00002** | 0.1283 | 0.0066 | **0.0000** |  |
| ICP-L | 0.00076 | 0.00003 | **0.00077** | **0.00002** | **0.00078** | **0.00003** | **0.00074** | **0.00002** | 0.0233 | **0.0000** | **0.0000** |  |
| SCP-R | 0.00086 | 0.00004 | 0.00085 | 0.00004 | 0.00087 | 0.00004 | 0.00084 | 0.00004 | 0.0500 | 0.2579 | 0.0167 |  |
| SCP-L | 0.00085 | 0.00004 | 0.00086 | 0.00004 | 0.00086 | 0.00003 | 0.00083 | 0.00004 | 0.0873 | 0.0056 | 0.0171 |  |
| CP-R | 0.00076 | 0.00006 | 0.00075 | 0.00005 | 0.00076 | 0.00004 | 0.00075 | 0.00003 | 0.4434 | 0.9860 | 0.6904 |  |
| CP-L | 0.00077 | 0.00003 | 0.00078 | 0.00004 | 0.00078 | 0.00004 | 0.00078 | 0.00003 | 0.2414 | 0.5666 | 0.9512 |  |
| ALIC-R | 0.00074 | 0.00005 | 0.00072 | 0.00003 | 0.00074 | 0.00005 | 0.00072 | 0.00003 | 0.0056 | 0.5047 | 0.3268 |  |
| ALIC-L | 0.00073 | 0.00004 | 0.00072 | 0.00004 | 0.00072 | 0.00002 | 0.00072 | 0.00003 | 0.0762 | 0.0854 | 0.2430 |  |
| PLIC-R | 0.00072 | 0.00004 | 0.00071 | 0.00004 | 0.00072 | 0.00002 | 0.00071 | 0.00002 | 0.0071 | 0.3217 | 0.1512 |  |
| PLIC-L | 0.00072 | 0.00003 | 0.00073 | 0.00002 | 0.00073 | 0.00003 | 0.00073 | 0.00002 | 0.4113 | 0.7823 | 0.5633 |  |
| RLIC-R | 0.00076 | 0.00005 | 0.00074 | 0.00006 | 0.00076 | 0.00003 | 0.00075 | 0.00004 | 0.1763 | 0.2986 | 0.7821 |  |
| RLIC-L | 0.00078 | 0.00003 | 0.00079 | 0.00003 | 0.00079 | 0.00004 | 0.00078 | 0.00003 | 0.3229 | 0.5380 | 0.1379 |  |
| ACR-R | 0.00078 | 0.00005 | 0.00077 | 0.00005 | 0.00079 | 0.00003 | 0.00078 | 0.00004 | 0.4294 | 0.4404 | 0.2598 |  |
| ACR-L | 0.00077 | 0.00004 | 0.00077 | 0.00005 | 0.00079 | 0.00003 | 0.00077 | 0.00003 | 0.2721 | 0.0414 | 0.0198 |  |
| SCR-R | 0.00074 | 0.00004 | 0.00074 | 0.00003 | 0.00075 | 0.00003 | 0.00073 | 0.00003 | 0.0284 | 0.0084 | 0.0195 |  |
| SCR-L | 0.00076 | 0.00004 | 0.00076 | 0.00003 | **0.00078** | **0.00002** | **0.00075** | **0.00003** | 0.4021 | 0.0993 | **0.0027** |  |
| PCR-R | 0.00078 | 0.00005 | 0.00078 | 0.00004 | 0.00080 | 0.00004 | 0.00078 | 0.00005 | 0.4514 | 0.6598 | 0.2994 |  |
| PCR-L | 0.00080 | 0.00005 | 0.00080 | 0.00003 | 0.00082 | 0.00003 | 0.00080 | 0.00004 | 0.4145 | 0.8198 | 0.1997 |  |
| PTR-R | 0.00079 | 0.00004 | 0.00077 | 0.00005 | 0.00078 | 0.00004 | 0.00078 | 0.00004 | 0.4768 | 0.3060 | 0.9763 |  |
| PTR-L | 0.00081 | 0.00005 | 0.00080 | 0.00005 | 0.00081 | 0.00003 | 0.00080 | 0.00004 | 0.6214 | 0.6415 | 0.6354 |  |
| SS-R | 0.00081 | 0.00005 | 0.00080 | 0.00004 | 0.00079 | 0.00004 | 0.00080 | 0.00004 | 0.4083 | 0.7681 | 0.5042 |  |
| SS-L | 0.00082 | 0.00004 | 0.00082 | 0.00004 | 0.00083 | 0.00003 | 0.00081 | 0.00003 | 0.8379 | 0.2152 | 0.1516 |  |
| EC-R | 0.00079 | 0.00005 | 0.00077 | 0.00005 | 0.00078 | 0.00005 | 0.00077 | 0.00004 | 0.0959 | 0.5280 | 0.7547 |  |
| EC-L | 0.00079 | 0.00005 | 0.00079 | 0.00004 | 0.00081 | 0.00004 | 0.00079 | 0.00004 | 0.8423 | 0.2051 | 0.0390 |  |
| CGC-R | 0.00070 | 0.00003 | 0.00069 | 0.00003 | 0.00069 | 0.00002 | 0.00070 | 0.00003 | 0.6556 | 0.4886 | 0.3019 |  |
| CGC-L | 0.00069 | 0.00003 | 0.00069 | 0.00003 | 0.00070 | 0.00003 | 0.00070 | 0.00003 | 0.5598 | 0.3244 | 0.6761 |  |
| CGH-R | 0.00063 | 0.00007 | 0.00063 | 0.00007 | 0.00063 | 0.00008 | 0.00065 | 0.00006 | 0.2405 | 0.2144 | 0.2816 |  |
| CGH-L | 0.00066 | 0.00006 | 0.00067 | 0.00006 | 0.00069 | 0.00005 | 0.00069 | 0.00004 | 0.0145 | 0.0470 | 0.8998 |  |
| FX/ST-R | 0.00076 | 0.00005 | 0.00074 | 0.00006 | 0.00074 | 0.00005 | 0.00076 | 0.00005 | 0.4692 | 0.3025 | 0.4654 |  |
| FX/ST-L | 0.00076 | 0.00004 | 0.00077 | 0.00003 | 0.00079 | 0.00005 | 0.00078 | 0.00004 | 0.0181 | 0.0495 | 0.3195 |  |
| SLF-R | 0.00075 | 0.00003 | 0.00075 | 0.00003 | 0.00077 | 0.00003 | 0.00075 | 0.00003 | 0.9971 | 0.6806 | 0.0770 |  |
| SLF-L | 0.00074 | 0.00004 | 0.00074 | 0.00003 | 0.00075 | 0.00003 | 0.00075 | 0.00003 | 0.5050 | 0.6039 | 0.7193 |  |
| SFO-R | 0.00073 | 0.00005 | 0.00071 | 0.00005 | 0.00072 | 0.00008 | 0.00070 | 0.00005 | 0.0210 | 0.2534 | 0.3388 |  |
| SFO-L | 0.00071 | 0.00006 | 0.00070 | 0.00006 | 0.00069 | 0.00003 | 0.00070 | 0.00005 | 0.1771 | 0.3084 | 0.7182 |  |
| IFO-R | 0.00077 | 0.00005 | 0.00077 | 0.00005 | 0.00079 | 0.00005 | 0.00075 | 0.00005 | 0.0496 | 0.0466 | 0.0126 |  |
| IFO-L | 0.00076 | 0.00006 | 0.00078 | 0.00006 | 0.00081 | 0.00007 | 0.00078 | 0.00005 | 0.2077 | 0.2917 | 0.0986 |  |
| UNC-R | 0.00091 | 0.00012 | 0.00090 | 0.00010 | 0.00088 | 0.00007 | 0.00088 | 0.00008 | 0.0399 | 0.1707 | 0.9794 |  |
| UNC-L | 0.00091 | 0.00012 | 0.00089 | 0.00010 | 0.00087 | 0.00008 | 0.00091 | 0.00011 | 0.7557 | 0.5913 | 0.1430 |  |

| MO | | | | | | | | | | | | |
| --- | --- | --- | --- | --- | --- | --- | --- | --- | --- | --- | --- | --- |
|  | **Group A**  **(n=27)** | | **Group B**  **(n=35)** | | **Group C**  **(n=16)** | | **Normal Controls (NCs)**  **(n=47)** | | ***Group A vs NCs*** | ***Group B vs NCs*** | ***Group C vs NCs*** |  |
|  | **Mean** | **SD** | **Mean** | **SD** | **Mean** | **SD** | **Mean** | **SD** | ***p*** | ***p*** | ***p*** |  |
| GCC | 0.87079 | 0.05504 | 0.88150 | 0.04795 | 0.89692 | 0.04233 | 0.86969 | 0.07485 | 0.7691 | 0.5558 | 0.2243 |  |
| BCC | 0.88256 | 0.03577 | 0.88572 | 0.03926 | 0.89288 | 0.03341 | 0.89156 | 0.03512 | 0.2087 | 0.0652 | 0.7738 |  |
| SCC | 0.91188 | 0.01964 | 0.90581 | 0.02479 | 0.89723 | 0.02897 | 0.89652 | 0.03426 | 0.0092 | 0.0221 | 0.6527 |  |
| FX | 0.83090 | 0.08784 | 0.80902 | 0.11682 | 0.82429 | 0.08539 | 0.80712 | 0.11069 | 0.3654 | 0.5350 | 0.7912 |  |
| CST-R | 0.54150 | 0.09275 | 0.51098 | 0.07702 | 0.48686 | 0.06684 | 0.55506 | 0.08596 | 0.4446 | 0.0351 | 0.0110 |  |
| CST-L | 0.54122 | 0.07506 | 0.53941 | 0.09228 | **0.50200** | **0.10682** | **0.59685** | **0.09593** | 0.0177 | 0.0035 | **0.0002** |  |
| ML-R | 0.72094 | 0.08148 | 0.74236 | 0.05559 | 0.74076 | 0.06890 | 0.70279 | 0.05903 | 0.2370 | 0.0067 | 0.0516 |  |
| ML-L | 0.81867 | 0.06030 | 0.82198 | 0.03799 | 0.80822 | 0.06321 | 0.79031 | 0.06299 | 0.0252 | 0.0075 | 0.0696 |  |
| ICP-R | **0.52069** | **0.06049** | 0.54354 | 0.05064 | 0.54663 | 0.03753 | **0.56903** | **0.05575** | **0.0002** | 0.0123 | 0.0591 |  |
| ICP-L | 0.58451 | 0.05634 | 0.58787 | 0.05674 | 0.58585 | 0.04695 | 0.58793 | 0.04909 | 0.8102 | 0.7071 | 0.8364 |  |
| SCP-R | 0.81975 | 0.02616 | 0.80287 | 0.03513 | 0.77932 | 0.05450 | 0.80831 | 0.04042 | 0.1154 | 0.7098 | 0.0605 |  |
| SCP-L | 0.81682 | 0.04113 | 0.80393 | 0.04425 | 0.76491 | 0.04795 | 0.80573 | 0.03981 | 0.0990 | 0.8288 | 0.0207 |  |
| CP-R | 0.80451 | 0.03233 | 0.78882 | 0.03401 | 0.78083 | 0.04435 | 0.79431 | 0.03957 | 0.1665 | 0.5429 | 0.7744 |  |
| CP-L | 0.74274 | 0.03933 | 0.75993 | 0.05108 | 0.74695 | 0.04234 | 0.75381 | 0.04454 | 0.4016 | 0.9381 | 0.9415 |  |
| ALIC-R | 0.77827 | 0.07003 | **0.81351** | **0.05911** | 0.78062 | 0.06145 | **0.75704** | **0.07192** | 0.0932 | **0.0000** | 0.1629 |  |
| ALIC-L | 0.80586 | 0.07176 | 0.78942 | 0.06129 | 0.77722 | 0.07045 | 0.78970 | 0.07745 | 0.2601 | 0.6939 | 0.5886 |  |
| PLIC-R | 0.81083 | 0.05361 | 0.81896 | 0.04252 | 0.77914 | 0.06166 | 0.80038 | 0.04817 | 0.0833 | 0.0073 | 0.8332 |  |
| PLIC-L | 0.79854 | 0.07031 | 0.80004 | 0.05259 | 0.77849 | 0.05963 | 0.79484 | 0.06326 | 0.8363 | 0.5134 | 0.7408 |  |
| RLIC-R | 0.64772 | 0.07584 | 0.67291 | 0.08095 | 0.65685 | 0.06131 | 0.64866 | 0.09107 | 0.8858 | 0.0979 | 0.3750 |  |
| RLIC-L | 0.63717 | 0.05474 | 0.68156 | 0.06645 | 0.64592 | 0.06689 | 0.64912 | 0.06192 | 0.4240 | 0.0131 | 0.5468 |  |
| ACR-R | 0.75199 | 0.05690 | 0.75543 | 0.05008 | 0.75741 | 0.06022 | 0.72661 | 0.06471 | 0.0439 | 0.0348 | 0.1752 |  |
| ACR-L | 0.71817 | 0.03989 | 0.71507 | 0.05864 | 0.73354 | 0.05005 | 0.70941 | 0.05394 | 0.3008 | 0.7931 | 0.2714 |  |
| SCR-R | 0.65120 | 0.09583 | 0.65071 | 0.07895 | 0.64809 | 0.07342 | 0.61582 | 0.08591 | 0.0352 | 0.0090 | 0.1023 |  |
| SCR-L | 0.66334 | 0.07479 | 0.65964 | 0.07032 | 0.66908 | 0.07288 | 0.64521 | 0.07430 | 0.2175 | 0.2397 | 0.2283 |  |
| PCR-R | 0.62510 | 0.09413 | 0.61699 | 0.11787 | 0.60492 | 0.09056 | 0.59633 | 0.11321 | 0.1735 | 0.1748 | 0.7745 |  |
| PCR-L | 0.58530 | 0.07778 | 0.60157 | 0.07744 | 0.61175 | 0.06306 | 0.58169 | 0.07336 | 0.7490 | 0.2606 | 0.2826 |  |
| PTR-R | 0.80027 | 0.04130 | 0.80495 | 0.04272 | 0.78221 | 0.06215 | 0.82062 | 0.05656 | 0.0448 | 0.1178 | 0.0027 |  |
| PTR-L | 0.80564 | 0.05020 | 0.78818 | 0.05557 | 0.78650 | 0.04404 | 0.81051 | 0.05049 | 0.5836 | 0.0428 | 0.0498 |  |
| SS-R | 0.63402 | 0.06703 | 0.66149 | 0.06570 | 0.63790 | 0.06054 | 0.63885 | 0.08882 | 0.5101 | 0.1506 | 0.9096 |  |
| SS-L | 0.67625 | 0.06612 | 0.67875 | 0.06267 | 0.65798 | 0.09441 | 0.68285 | 0.06585 | 0.9346 | 0.6600 | 0.4182 |  |
| EC-R | 0.52142 | 0.07715 | 0.53053 | 0.05518 | 0.56085 | 0.06823 | 0.53113 | 0.06578 | 0.4487 | 0.7172 | 0.1627 |  |
| EC-L | 0.60654 | 0.04178 | 0.61952 | 0.05247 | 0.60885 | 0.06242 | 0.61780 | 0.05250 | 0.2949 | 0.7439 | 0.5631 |  |
| CGC-R | 0.63799 | 0.08830 | 0.65592 | 0.08706 | 0.67148 | 0.06541 | 0.62550 | 0.09169 | 0.5900 | 0.2184 | 0.1098 |  |
| CGC-L | 0.64693 | 0.08922 | 0.69191 | 0.08655 | 0.67308 | 0.10438 | 0.65533 | 0.08557 | 0.5803 | 0.1362 | 0.9155 |  |
| CGH-R | 0.70211 | 0.09276 | 0.71818 | 0.11915 | 0.72385 | 0.09083 | 0.70700 | 0.09676 | 0.9847 | 0.6155 | 0.6232 |  |
| CGH-L | **0.76798** | **0.08386** | 0.75236 | 0.06131 | 0.75838 | 0.04429 | **0.72096** | **0.08770** | **0.0016** | 0.0354 | 0.0489 |  |
| FX/ST-R | 0.59230 | 0.09188 | 0.59928 | 0.09019 | 0.60289 | 0.08750 | 0.62293 | 0.08249 | 0.0847 | 0.0498 | 0.3366 |  |
| FX/ST-L | 0.58700 | 0.07915 | 0.59199 | 0.08002 | 0.60192 | 0.08764 | 0.60577 | 0.10078 | 0.2131 | 0.1140 | 0.5842 |  |
| SLF-R | 0.58070 | 0.05383 | 0.60563 | 0.05464 | 0.58031 | 0.04652 | 0.58485 | 0.03882 | 0.8555 | 0.0504 | 0.7532 |  |
| SLF-L | 0.55056 | 0.05515 | 0.55468 | 0.07003 | 0.52790 | 0.04668 | 0.52997 | 0.05443 | 0.0481 | 0.0139 | 0.9018 |  |
| SFO-R | 0.72372 | 0.10392 | **0.76824** | **0.07878** | 0.70790 | 0.08512 | **0.69646** | **0.11675** | 0.2321 | **0.0010** | 0.3929 |  |
| SFO-L | 0.81837 | 0.06403 | 0.80589 | 0.10085 | 0.79587 | 0.09457 | 0.77982 | 0.11256 | 0.0642 | 0.1592 | 0.4470 |  |
| IFO-R | 0.65649 | 0.15001 | 0.67436 | 0.11652 | 0.67307 | 0.10616 | 0.69187 | 0.12569 | 0.1702 | 0.3498 | 0.2936 |  |
| IFO-L | 0.72939 | 0.13978 | 0.75420 | 0.11291 | 0.73907 | 0.12071 | 0.74375 | 0.11907 | 0.4415 | 0.9975 | 0.7145 |  |
| UNC-R | 0.85479 | 0.07730 | 0.85644 | 0.07265 | 0.83938 | 0.07374 | 0.84470 | 0.08795 | 0.5345 | 0.2496 | 0.6396 |  |
| UNC-L | 0.85354 | 0.08900 | 0.81770 | 0.14450 | 0.79520 | 0.09605 | 0.84339 | 0.09035 | 0.6341 | 0.2107 | 0.0860 |  |
